# Iron-sulphur protein catalysed [4+2] cycloadditions in natural product biosynthesis

**DOI:** 10.1038/s41467-024-50142-1

**Published:** 2024-07-10

**Authors:** Yu Zheng, Katsuyuki Sakai, Kohei Watanabe, Hiroshi Takagi, Yumi Sato-Shiozaki, Yuko Misumi, Yohei Miyanoiri, Genji Kurisu, Toshihiko Nogawa, Ryo Takita, Shunji Takahashi

**Affiliations:** 1 Natural Product Biosynthesis Research Unit, RIKEN Center for Sustainable Resource Science, Saitama, 351-0198 Japan; 2 Graduate School of Pharmaceutical Sciences, The University of Tokyo, Tokyo, 113-0033 Japan; 3 Institute for Protein Research, Osaka University, Osaka, 565-0871 Japan; 4 Molecular Structure Characterization Unit, RIKEN Center for Sustainable Resource Science, Saitama, 351-0198 Japan; 5 Graduate School of Pharmaceutical Sciences, University of Shizuoka, Shizuoka, 422-8526 Japan

**Keywords:** Biocatalysis, Computational chemistry, Biocatalysis

## Abstract

To the best of our knowledge, enzymes that catalyse intramolecular Diels-Alder ([4+2] cycloaddition) reactions are frequently reported in natural product biosynthesis; however, no native enzymes utilising Lewis acid catalysis have been reported. Verticilactam is a representative member of polycyclic macrolactams, presumably produced by spontaneous cycloaddition. We report that the intramolecular [4+2] cycloadditions can be significantly accelerated by ferredoxins (Fds), a class of small iron-sulphur (Fe-S) proteins. Through iron atom substitution by Lewis acidic gallium (Ga) iron and computational calculations, we confirm that the ubiquitous Fe-S cluster efficiently functions as Lewis acid to accelerate the tandem [4+2] cycloaddition and Michael addition reactions by lowering free energy barriers. Our work highlights Nature’s ingenious strategy to generate complex molecule structures using the ubiquitous Fe-S protein. Furthermore, our study sheds light on the future design of Fd as a versatile Lewis acid catalyst for [4+2] cycloaddition reactions.

## Abstract (web summary)

Native enzymes using Lewis acid catalysis in intramolecular [4+2] cycloaddition remain unreported. Herein, the authors report the ferredoxins catalyzed [4+2] cycloaddition in polycyclic macrolactam verticilactam biosynthesis using a transition-metal center.

## Introduction

The Diels–Alder reaction is the [4 + 2] cycloaddition of a conjugated diene and a substituted dienophile to form a substituted cyclohexene derivative and up to four contiguous stereogenic centres^1^. Since its discovery in 1928 by the German chemists Otto Diels and Kurt Alder, this Nobel Prize-winning reaction has been widely used as a powerful transformation in synthetic organic chemistry to significantly increase the complexity of molecular structures^2^. Accordingly, numerous inorganic and organic catalysts have been developed to enhance the reactivity and selectivity of the reaction. One convenient method is to utilise Lewis acid catalysis, which can significantly accelerate the reaction by complexing to the dienophile to reduce the HOMO_diene_–LUMO_dienophile_ energy gap^3^. This rate enhancement can be up to a million-fold in water in the presence of transition metal ions^4^. Currently, the Diels–Alder reaction is frequently considered as a synchronous concerted [4 + 2] cycloaddition reaction according to the Woodward–Hoffmann rules^5^, although stepwise diradical manner, stepwise zwitterionic manner, and dynamically concerted and stepwise pathways have also been proposed^6,7^.

Natural products presumably biosynthesised by the Diels–Alder reaction are frequently reported in the literature^8^. In 2011, Kim et al. reported SpnF, the first stand-alone Diels–Alderase ([4 + 2] cyclase) that exclusively catalyses the Diels–Alder reaction in the biosynthesis of spinosyn A^9^. Natural Diels–Alderases ([4 + 2] cyclases) of diverse origin and protein structure have been reported and are represented by the *S*-adenosyl-L-methionine (SAM)-dependent SpnF^9^, flavin adenine dinucleotide (FAD)-dependent PyrE3 and PyrI4-type cyclases in the biosynthesis of spirotetronates and spirotetramates^10^, Fsa2-family cyclases catalysing decalin formation in the biosynthesis of pyrrolin-2-one and pyrrolidine-2,4-dione^11^, and IdmH-type cyclases with Snoal-like *α* + *β* barrel scaffold^12^ (Supplementary Fig. 1). Most [4 + 2] cyclases, such as SpnF^13^, are characterised by a substrate-trapping catalytic mechanism in the hydrophobic active site cavity to achieve the stereospecific [4 + 2] cycloadditions by overcoming the higher transition state (TS) activation energy barrier (Supplementary Fig. 2). In contrast, natural enzymes using Lewis acid catalysis for [4 + 2] cycloaddition have not been reported.

**Fig. 1:**
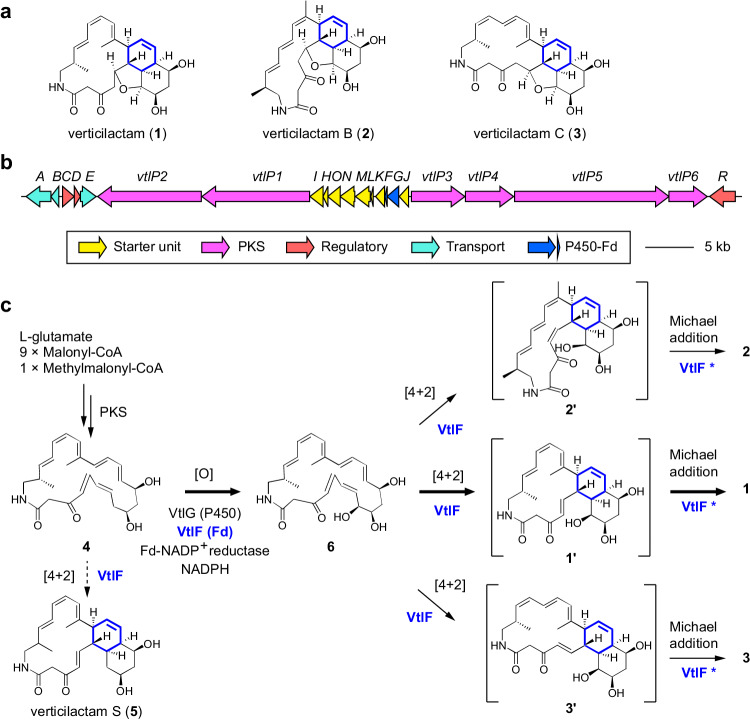
Biosynthesis of verticilactams. **a** Chemical structures of verticilactam (**1**) and verticilactams B (**2**) and C (**3**). **b** Biosynthetic gene cluster of **1** from *S. spiroverticillatus* JC-8444^15^. **c** The proposed biosynthetic pathway for verticilactams. Solid arrows indicate a physiological pathway, whereas the dashed arrow shows an abiological reaction only observed by in vitro conversion. *VtlF possibly accelerates Michael addition reaction based on DFT calculations.

Verticilactam (**1**, Fig. 1a) is a tetracyclic macrolactam metabolite isolated from *Streptomyces spiroverticillatus* (*S. spiroverticillatus*) JC-8444 and shows moderate activity against the malaria parasite *Plasmodium falciparum* 3D7^14,15^. Recently, heterologous expression of the *vtl* gene cluster (Fig. 1b) in *Streptomyces avermitilis* (*S. avermitilis*) SUKA17 strain led to two isolated geometric isomers, verticilactams B (**2**) and C (**3**)^15^. Verticilactams **1**–**3** have the same octalin moiety and are proposed to be generated by intramolecular [4 + 2] cycloaddition of a monocyclic precursor in type I polyketide synthase (PKS) pathway, like other polycyclic macrolactams (Supplementary Fig. 3). Currently, [4 + 2] cycloadditions in the biosynthesis of polycyclic macrolactams are thought to be spontaneous because homologues of known [4 + 2] cyclases or candidate enzymes in these gene clusters are lacking^15–19^ (Supplementary Fig. 4a). A proven example is the nonenzymatic conversion of sceliphrolactam into tripartilactam (niizalactam C) after incubation at room temperature^19^ (Supplementary Fig. 3). Another example is the formation of stereoisomeric cyclamenols B and C from the parent cyclamenol A^20^, suggesting the absence of an enzyme controlling the stereochemistry of the [4 + 2] cycloaddition.

Ferredoxins (Fds) are widely distributed small iron–sulfur (Fe–S) proteins adopting the *α* + *β* fold, first purified from anaerobic *Clostridium pasteurianum* and spinach chloroplast in 1962, that act as versatile electron carriers in various biological processes including photosynthesis, carbon assimilation, nitrogen fixation, among others^21–23^. In nature, Fds contain three main types of Fe–S clusters, coordinated predominantly by the conserved cysteine thiolate ligands of the protein scaffold^24^ (Supplementary Fig. 5). The plant-type Fds comprise a rhombic [2Fe–2S] cluster bound to the Cys-X_4_-Cys-X_2_-Cys-X_*n*_-Cys motif. In contrast, the bacterial-type Fds comprise a cubic cluster that is coordinated by the Cys-X_2_-(Cys or non-Cys)-X_2_-Cys-X_*n*_-Cys-Pro motif (Supplementary Fig. 4b). Given that the inorganic Fe^2+^/Fe^3+^ and S^2−^ are abundant elements in Earth’s early marine environment and they can spontaneously assemble into redox-active clusters, the Fe–S clusters are among the most versatile and ancient bioinorganic cofactors^25^. Their functions can be exemplified as oxygen sensing in the [2Fe–2S]/[4Fe–4S] cluster fumarate nitrate reduction regulator, sulfur donation in the [4Fe–4S] cluster lipoyl synthase LipA, structural stability in the [4Fe–4S] cluster DNA polymerase ε, reductive cleavage of SAM in the [4Fe–4S] cluster radical SAM enzyme, and Lewis acid catalysis of citrate isomerisation in the [4Fe–4S] cluster aconitase^24,26^.

In this study, we addressed the cryptical mechanistic mystery of post-PKS modification in verticilactams biosynthesis through gene disruption, in vitro enzyme characterisation, and computational calculations (Fig. 1c). Here, we find the Fds-catalysed intramolecular [4 + 2] cycloadditions, initially discovered using spinach Fd and verified using actinobacterial Fd (VtlF). Based on catalytic activity evaluation using apo-Fds, gallium-substituted Fd, and density functional theory (DFT) calculations, we concluded that Fds utilise the Fe–S cluster as an efficient Lewis acid to accelerate the tandem [4 + 2] cycloaddition and Michael addition reactions, in contrast with SpnF and other natural [4 + 2] cyclases that use substrate-trapping manner in the active site cavity. Our findings highlight Nature’s ingenious strategy to create complex molecular structures using the small, ubiquitous Fe–S cluster protein on the 60th anniversary of its discovery.

## Results and discussion

### Characterisation of VtlG (P450) as a monooxygenase in verticilactam biosynthesis

**Fig. 2:**
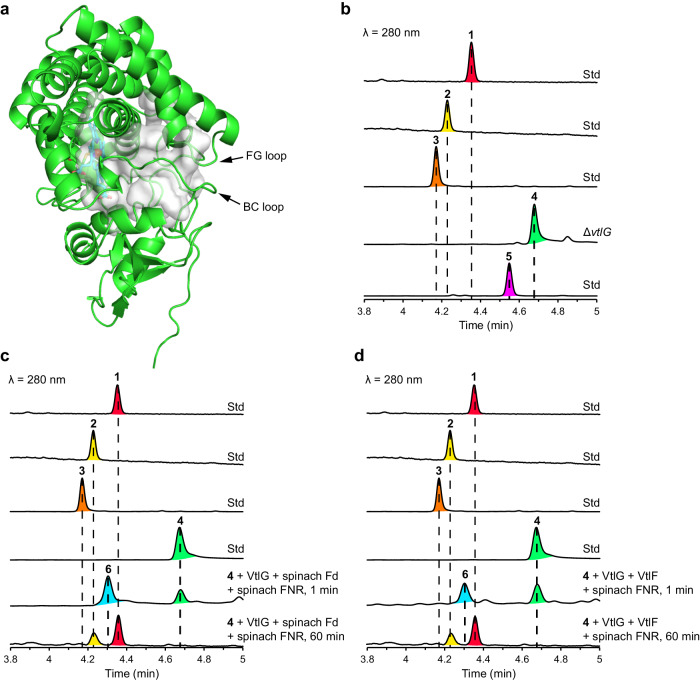
Functional characterisation of VtlG (P450). **a** AlphaFold2^67^ predicted the structure of VtlG. The haem cofactor shown in cyan was modelled from PDB 6J85 as a template. **b** Compound **4** accumulated in the *vtlG* disruptant, and compound **5** nonenzymatically converted from **4** during isolation. Time-dependent conversion of 1 μM **4** into **1** in vitro in the presence of 0.5 μM VtlG, 0.1 mg/mL spinach Fd (**c**) or 0.1 mg/mL VtlF (**d**), 1 unit/mL spinach FNR, and 100 μM NADPH. The reactions were performed in 50 mM Tris-HCl (pH 7.5) at 30 °C, and the reaction products were monitored by UPLC–MS.

As previously described^15^, the *vtl* gene cluster consists of 22 genes, including 8 responsible for the incorporation of a β-amino acid starter unit for the polyketide assembly line, 6 type I PKS genes responsible for polyketide chain elongation, 3 regulatory genes, 3 transporters, and a P450–Fd combination presumably accountable for hydroxylation of the polyketide skeleton (Fig. 1b). A similar gene composition is also observed in available gene clusters for polycyclic macrolactams, such as mirilactams^18^, tripartilactam^19^, and macrotermycins^16^ (Supplementary Fig. 4a). Among these gene clusters, P450s are the common enzymes putatively involved in post-PKS modification in the macrolactam biosynthetic pathways^27^. Furthermore, P450s adopt a closed-to-open motion in catalysis^28^ (Fig. 2a), resembling the lid-like trapping manner of previously reported [4 + 2] cyclases.

To elucidate the biosynthetic pathway of **1**, we first disrupted the *vtlG* (P450) gene in the bacterial artificial chromosome vector^15^ (Supplementary Fig. 6), transformed the plasmid into *S. avermitilis* SUKA17 strain, and analysed its metabolites by liquid chromatography–mass spectrometry (LC–MS). We observed that the *vtlG* disruptant abolished the production of **1**, but accumulated compound **4** (Fig. 2b). Based on the biosynthetic logic^15,27^, HR-ESI-TOF-MS analysis (*m/z* 412.2484 [M + H]^+^) (Supplementary Fig. 7), and its absorption spectrum (Supplementary Fig. 8), we speculated compound **4** as a pentaene macrolactam and the biosynthetic precursor of **1** (Fig. 1c). Like other unstable polyene macrolactams that spontaneously decompose into unknown products^27^, **4** was also unstable upon NMR analysis. In turn, we isolated a stable compound **5**, which was converted from **4** during purification from the culture broth of *vtlG* disruptant (Fig. 2b) and determined its structure as a tricyclic macrolactam, verticilactam S (Fig. 1c, Supplementary Method, Supplementary Figs. 7–18, Supplementary Table 1). The isolation of the polyene macrolactam **4** and polycyclic macrolactam **5** from the *vtlG* disruptant suggested the involvement of the VtlG reaction in [4 + 2] cycloaddition. However, we could not rule out spontaneous cyclisation.

To examine the function of VtlG, we purified the recombinant protein from *Escherichia coli* (*E. coli*) (Supplementary Fig. 19a, b). We reconstructed the in vitro reaction system using the commonly used commercial spinach Fd and spinach Fd-NADP^+^ reductase (FNR) as surrogate redox partners in bacterial class I P450s^29^. Initiated by adding NADPH as an electron donor, VtlG rapidly converted **4** into **6** (Fig. 2c). HR-ESI-TOF-MS analysis indicated compound **6** (*m/z* 428.2440 [M + H^+^]) (Supplementary Fig. 7) as a mono-hydroxylation reaction product of **4**. In addition, **6** retained a similar pentaene absorption spectrum to **4** (Supplementary Fig. 8). The *K*_M_ and *k*_cat_ values of VtlG-catalysed hydroxylation against **4** were determined as 0.84 ± 0.26 µM and 6.47 ± 0.82 min^−1^ (*k*_cat_/*K*_M_ = 7.70 min^−1^ µM^−1^), respectively (Supplementary Fig. 19c). In contrast with **4**, polycyclic **5** was not recognised as a VtlG substrate. These results strongly suggest that **4** is the physiological substrate for VtlG, and mono-hydroxylation occurs before [4 + 2] cycloaddition (Fig. 1c).

### Spinach Fd-catalysed [4 + 2] cycloadditions

**Fig. 3:**
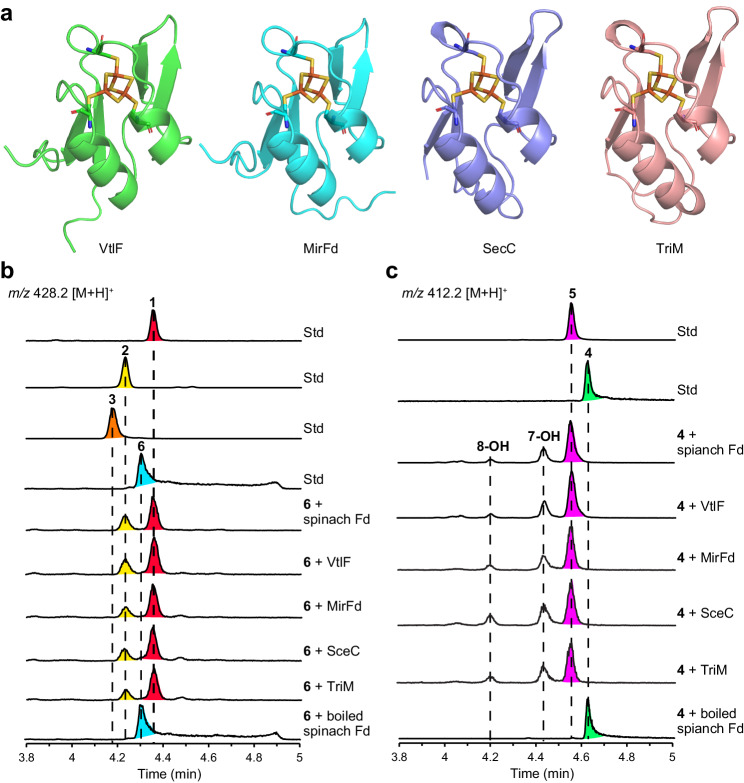
Fds-catalysed [4 + 2] cycloadditions. **a** AlphaFold2^67^ predicted VtlF, MirFd, SceC, and TriM structures. The [3Fe–4S] cluster was modelled from PDB 1SJ1 and coordinated by three cysteine residues in each Fd. In vitro conversion of 1 μM **6** into **1** (**b**) and 1 μM **4** into **5** (**c**) in the presence of 50 μM spinach Fd, VtlF, MirFd, SceC, and TriM, respectively. The reactions were performed in 50 mM Tris-HCl (pH 7.5) at 30 °C for 30 min. The reaction products were monitored by UPLC–MS. Boiled spinach Fd was the negative control.

Compound **6** is proposed to undergo cascade intramolecular [4 + 2] cycloaddition and Michael addition reactions to yield the final product **1**. Indeed, **6** was converted into **1** in the prolonged VtlG reaction (Fig. 2c), therefore unambiguously confirming **4** and **6** as the on-pathway biosynthetic intermediates of **1**. Similar to the nonenzymatic conversion of sceliphrolactam into tripartilactam^19^, spontaneous conversion of **6** into **1** could also occur but takes nearly 2 weeks (Supplementary Fig. 20). This tremendous gap in reaction rate strongly suggests an enzyme in the VtlG reaction system that may significantly accelerate the [4 + 2] cycloaddition reaction^30^. Next, considering that [4 + 2] cycloaddition is a non-redox reaction and Fd may play a structural role in P450 substrate binding^28^, we performed enzyme reactions with compound **6** in the presence or absence of VtlG, spinach Fd, spinach FNR, and NADPH, respectively. Surprisingly, commercial spinach Fd alone rapidly converted **6** into **1**. In contrast, VtlG alone showed no detectable activity (Supplementary Fig. 21). We then expressed and purified recombinant spinach Fd (Fd I) from *E. coli*. Using **6** and **4** as substrates, the recombinant spinach Fd rapidly converted **6** into **1** and **4** into **5** in the enzyme reaction in 30 min, respectively (Fig. 3). We also determined the steady-state kinetic parameters (*K*_M_ = 0.35 ± 0.08 μM, *k*_cat_ = 0.0039 ± 0.0002 min^−1^, and *k*_cat_/*K*_M_ = 0.011 min^−1^ μM^−1^) for compound **4** using commercial spinach Fd (Supplementary Fig. 22). In the absence of spinach Fd, conversion of **4** into **5** is negligible even after incubation at 30 °C for 1 month (Supplementary Fig. 20), indicating that cycloaddition of **4** is an abiological reaction. We could not obtain the exact nonenzymatic reaction rate because of the slow conversion; however, there is no doubt that spinach Fd could significantly accelerate the [4 + 2] cycloadditions of **6** and **4**. Nonetheless, these results were surprising because the spinach Fd containing a [2Fe–2S](Cys)_4_ cluster is widely recognised as a versatile electron carrier^22,23^. In addition, although protein–protein interactions between Fd and Fd-dependent enzymes have been extensively studied^21–23,29^, the interaction between Fd and organic molecules remains rare.

### VtlF (Fd) has a bifunctional activity of electron donation and [4 + 2] cycloadditions

**Fig. 4:**
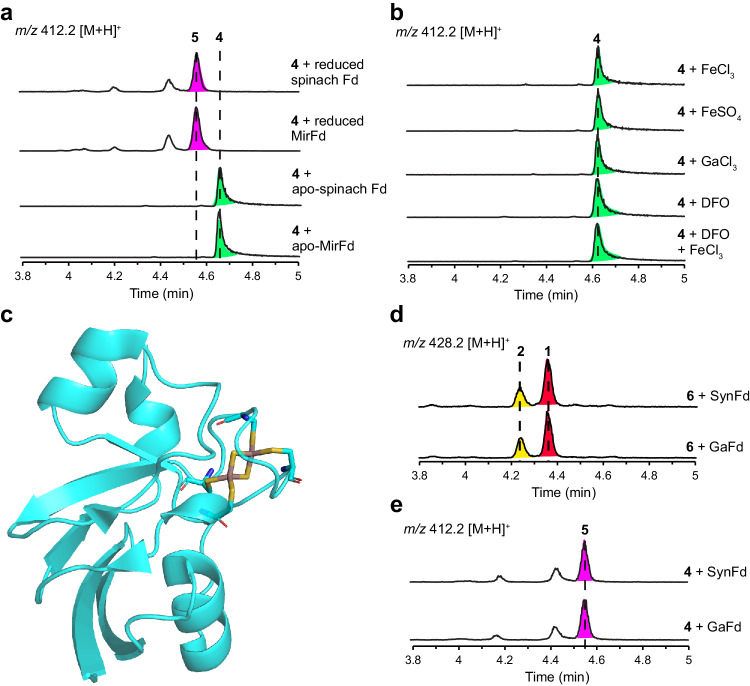
Characterisation of Fe–S cluster as efficient Lewis acid. **a** 1 μM **4** was incubated with 50 μM of apo-spinach Fd mutant, apo-MirFd mutant, reduced spinach Fd with [2Fe–2S](Cys)_4_^3−^ cluster, and reduced MirFd with [3Fe–4S](Cys)_3_^3−^ cluster, respectively. Fds were reduced with 1 mM sodium dithionite. **b** 1 μM **4** was incubated with 1 mM FeCl_3_, 1 mM FeSO_4_, 1 mM GaCl_3_, 1 mM desferrioxamine (DFO), and 1 mM DFO with 1 mM FeCl_3_. The in vitro reactions were conducted at 30 °C in 50 mM Tris-HCl (pH 7.5) for 30 min. **c** The crystal structure of the gallium-substituted *Synechocystis* sp. PCC 6803 Fd (SynFd), GaFd (PDB 5AUK). In vitro conversion of 1 μM **6** into **1** in 15 min (**d**) and 1 μM **4** into **5** in 30 min (**e**) in the presence of 200 μM SynFd and GaFd, respectively.

Based on the findings of spinach Fd, we were motivated to evaluate the activity of VtlF, the endogenous Fd that is located adjacent to *vtlG* in the *vtl* gene cluster (Fig. 1b). Like other *Streptomyces* Fds^31,32^, VtlF is a [3Fe–4S] Fd containing a Cys-X_2_-Ala-X_2_-Cys-X_34_-Cys-Pro motif (Fig. 3a, Supplementary Fig. 4b). We purified His-tagged VtlF from *E. coli* (Supplementary Fig. 23) and reconstructed the in vitro reaction system using VtlG, spinach FNR, and NADPH. As expected, **4** was rapidly converted into **6** and then gradually converted into **1** (Fig. 2d) like that observed using spinach Fd (Fig. 2c). When reacted with the substrates **6** and **4** alone, VtlF also rapidly converted **6** into **1** (Fig. 3b) and **4** into **5** (Fig. 3c), following a similar manner to spinach Fd. Collectively, these results confirmed the physiological roles of VtlF in verticilactams biosynthesis to transfer electrons for the P450 monooxygenase activity of VtlG to produce **6** from **4** and catalyse [4 + 2] cycloadditions after VtlG-catalysed mono-hydroxylation reaction to produce **1** from **6**. Notably, although **5** can be produced from **4** in the presence of Fds by the in vitro enzyme reactions, this is not the main physiological pathway given the significant difference in catalytic efficiency between the VtlG-catalysed mono-hydroxylation (Supplementary Fig. 19) and Fd-catalysed [4 + 2] cycloaddition (Supplementary Fig. 22). Accordingly, we completed the proposed biosynthetic pathway of verticilactams (**1**–**3**) in Fig. 1c.

Next, to examine the ubiquity of the above observations, we purified MirFd, SceC, and TriM (Supplementary Fig. 23), the counterparts of VtlF found in mirilactams^18^, sceliphrolactam^17^, and tripartilactam^19^ biosynthetic gene clusters (Supplementary Fig. 4), respectively. As expected, all the actinobacterial Fds readily converted **6** into **1** (Fig. 3b) and **4** into **5** (Fig. 3c) by the in vitro enzyme reactions, suggesting that MirFd, SceC, and TriM may have equivalent functions in mirilactams and tripartilactam pathways. We also determined the steady-state kinetic parameters (*K*_M_ = 0.66 ± 0.15 μM, *k*_cat_ = 0.0430 ± 0.0029 min^−1^, and *k*_cat_/*K*_M_ = 0.065 min^−1^ μM^−1^) for compound **4** using MirFd (Supplementary Fig. 22). To date, intramolecular [4 + 2] cycloadditions in the biosynthesis of polycyclic macrolactams are thought to be spontaneous due to the polyene nature of the monocyclic presursors^15–19^. Our findings suggest that cycloadditions are significantly accelerated with an endogenous Fd in the biosynthetic gene cluster. If an endogenous Fd is absent, taking the *mte* gene cluster, for instance, intramolecular [4 + 2] cycloadditions can still be catalysed by Fds, given the multiple copies of genes in each living organism^29^. Together, our results unambiguously confirmed the Fds-catalysed [4 + 2] cycloadditions in natural product biosynthesis.

### [4 + 2] cycloadditions are Fe–S cluster dependent

The crystal structure of spinach [2Fe–2S](Cys)_4_ Fd (Supplementary Fig. 5b) is quite different from the predicted structures of actinobacterial [3Fe–4S](Cys)_3_ Fds (Fig. 3a). Furthermore, unlike the previously reported [4 + 2] cyclases which utilise the substrate-trapping catalytic mechanism (Supplementary Figs. 1, 2), Fds are small Fe–S proteins lacking an obvious substrate cavity (Fig. 3a, Supplementary Fig. 5b), suggesting that they may have a different catalytic mechanism. We focused on the Fe–S cluster cofactor to gain further mechanistic insight into the Fds-catalysed [4 + 2] cycloadditions. First, we investigated the effect of the oxidation state of the Fe–S cluster by reducing with sodium dithionite^21,22^. As a result, the catalytic efficiency was not affected after reducing the oxidised [2Fe–2S](Cys)_4_^2−^ cluster into [2Fe–2S](Cys)_4_^3−^ in the spinach Fd or reducing the oxidised [3Fe–4S](Cys)_3_^2−^ cluster into [3Fe–4S](Cys)_3_^3−^ in the actinobacterial MirFd^24^ (Fig. 4a). Next, we prepared apo-forms of spinach [2Fe–2S] Fd and actinobacterial [3Fe–4S] MirFd by ethylenediaminetetraacetic acid (EDTA) treatment^33^. We also performed site-directed mutagenesis to replace the Fe–S cluster binding cysteine residue into alanine and purified the apo-form mutants of spinach Fd and MirFd from *E. coli* (Supplementary Fig. 24). Using **4** as a model substrate, we confirmed that the apo-Fd mutants completely abolished the [4 + 2] cyclase activity in the enzyme reactions (Fig. 4a), suggesting that Fds-catalysed [4 + 2] cycloaddition is Fe–S cluster dependent. Interestingly, the inorganic transition metal ions such as FeCl_3_, FeSO_4_, and GaCl_3_, and iron-chelating compounds such as desferrioxamine (DFO) showed no catalytic activity against substrate **4** (Fig. 4b), suggesting that the protein scaffolds of Fds are also essential for the activity.

### Fe–S clusters function as efficient Lewis acid for [4 + 2] cycloaddition reaction

The [4Fe–4S](Cys)_3_ cluster in aconitase is known to function as an efficient Lewis acid to catalyse the isomerisation of citrate to isocitrate^24,26^. In enzymes where the Fe–S cluster acts solely as a Lewis acid, gallium substitution with a Lewis acidic but redox-independent Ga–S cluster represents an ideal functional analogue in catalysis^34^. To investigate whether the Fe–S cluster functions as Lewis acid in the Fds-catalysed [4 + 2] cycloadditions^3^, we prepared recombinant Fd from *Synechocystis* sp. PCC 6803 (SynFd) containing a [2Fe–2S](Cys)_4_ cluster by *E. coli* expression (Supplementary Fig. 25) and Ga-substituted *Synechocystis* sp. PCC 6803 Fd (GaFd)^35^ (Fig. 4c). When reacted with compounds **4** and **6**, respectively, both the [2Fe–2S] SynFd and the [2Ga–2S] GaFd converted **6** into **1** (Fig. 4d) and **4** into **5** (Fig. 4e), like the spinach [2Fe–2S] Fd and the actinobacterial [3Fe–4S] Fds (Fig. 3). We have also performed NMR analysis of the [^15^N]-labelled GaFd with substrate **4** (Supplementary Fig. 26), indicating again the absence of any catalytic amino acid residues^12,36^. These results confirmed the ubiquity of Fds-catalysed [4 + 2] cycloadditions and the Lewis acidity of the Fe–S cluster cofactor in the catalytic activity of Fds.

This finding is significant because it may represent the experimentally confirmed natural enzyme to use efficient Lewis acid catalysis in [4 + 2] cycloaddition in natural product biosynthesis. A previously proposed example is the multi-functional macrophomate synthase, which may utilise the Lewis acidity of the magnesium ion in the active site to promote decarboxylation and [4 + 2] cycloaddition reactions in macrophomate biosynthesis^37^. However, this enzyme is a competent aldolase employing a Michael–aldol mechanism^38,39^. Since discovering the 100-year-old Diels–Alder reaction, efforts have been made to design and engineer inorganic or protein scaffold ligands to achieve efficient Lewis acidity by transition metal ions^40–42^. For instance, Braconi and Cramer developed a chiral *bis*-dihydroisoquinoline scaffold ligand to promote asymmetric Fe-catalysed [4 + 2] cycloaddition of inactivated dienes^42^. Fujiwara et al. developed a bioinspired cationic iron (III) porphyrin catalyst to afford pyrans under relatively mild conditions^40^. Basler et al. developed an artificial zinc-binding peptide for an abiological hetero-Diels–Alder reaction^41^. However, these de novo designing processes can be time-consuming and may require extensive computational, evolutionary, and trial-and-error validation efforts. In contrast, the naturally designed Fds containing the ancient and versatile Fe–S cluster cofactors are ubiquitous in most living organisms and can be easily obtained^21,22,43^.

### DFT calculations for the verticilactams biosynthetic pathway

**Fig. 5:**
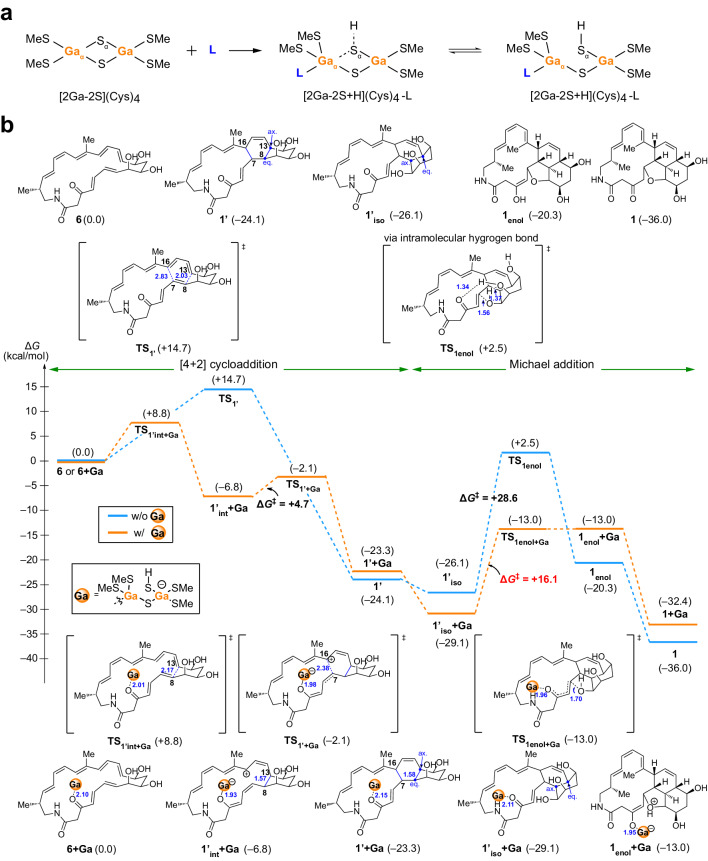
Proposed cluster–ligand interaction mode and the energy diagram based on DFT calculations. **a** In the [2Ga–2S + H](Cys)_4_-L model, the reactant molecule L binds to the Ga_*α*_ atom through a fifth coordination bond and the Ga–S cluster is stabilised by protonation. The cysteine residues were simplified to methanethiolate groups (SMe). **b** DFT calculations for the conversion of **6** to **1**. Activation-free energies (Δ*G*^‡^) calculated at the M06-2X/SDD and 6-311 + G** (SCRF = CPCM, water) level of theory are given in kcal mol^−1^ and distances in Å. The Diels–Alder [4 + 2] cycloaddition moves from left to right, followed by the Michael addition to the right. Energies and geometric structures of transition states (TS) and intermediates (int) with or without [2Ga–2S + H](Cys)_4_-L model are given below and above the relative energy diagram.

To gain energetic insights into the proposed **1** biosynthetic pathway (Fig. 1c) and understand how Fds accelerate the [4 + 2] cycloaddition and Michael addition reactions, we first performed DFT calculations using compound **6** without enzyme at the M06-2X/SDD&6-311 + G**/(SCRF = CPCM, water) level of theory. As illustrated in Fig. 5 and Supplementary Fig. 27a, the nonenzymatic [4 + 2] cycloaddition of **6** favours an *exo*-pathway to form **1′** among the four TSs, leading to the final formation of **1**. The calculated activation barriers for the other cycloaddition pathways (**TS**_**7**_, **TS**_**8**_, and **TS**_**9**_) forming stereoisomers **7**–**9** (more than 17.2 kcal mol^−1^) are much higher than the *exo*-pathway **TS**_**1′**_ (14.7 kcal mol^−1^), therefore are unlikely to occur. Furthermore, the *exo*-pathway **TS**_**1′**_ proceeds in an asynchronous concerted reaction with the forming C_7_–C_16_ bond and C_8_–C_13_ bond at 2.83 and 2.03 Å lengths, respectively. A similar result is also observed for compound **4**, which favours the *exo*-TS reaction leading to the formation of **5** (Supplementary Fig. 27b). Regarding Michael addition of **1′**, we observed relatively high activation barriers either via an intramolecular hydrogen bonding (**TS**_**1enol**_, 28.6 kcal mol^−1^), the aid of an additional water molecule (**TS**_**1enol+H2O**_, 28.9 kcal mol^−1^) (Fig. 5, Supplementary Fig. 28). These results are consistent with the slow in vitro nonenzymatic conversion of **6** into **1** (Supplementary Fig. 20a).

Next, it is known that the surface of Fds is overall negatively charged, allowing electrostatic interactions with redox partners such as P450s, FNR, and other proteins^44,45^, and that Fds have considerable solvent access to the Fe–S cluster cofactors^46,47^. It is therefore reasonable that the reactant molecules **4** and **6** can closely approach the Fe–S cluster cofactors via the more open and solvated surface region of the Fds^46,47^. Given the difficulties in handling 3d transition metals in DFT calculations, we included a [2Ga–2S](Cys)_4_ cluster from *Synechocystis* GaFd (PDB ID: 5AUK) to function as a Lewis acid in the calculations. Moreover, the [^15^N] NMR analysis at 277 and 298 K revealed that the Cys residues coordinating the [2Ga–2S] cluster exhibited no discernible chemical shifts although the [4 + 2] cycloaddition reaction proceeded during NMR measurement (Supplementary Fig. 26), suggesting the absence of detectable cleavage of coordination bonds between the cluster and Cys residues^35^. These observations were further verified by 5,5′-dithiobis-(2-nitrobenzoic acid) assay in the presence of reactant molecule **4** (Supplementary Fig. 29), again suggesting that there is no detectable cleavage of the cluster-coordinating Cys residues to release free cysteine thiol groups^48^. Based on these experimental results, we assumed the [2Ga–2S + H](Cys)_4_-L model for the [2Ga–2S] cluster–reactant molecule L interaction (Fig. 5a), in which the reactant molecule L (compounds **4** or **6**) forms a fifth coordination bond with the Ga_*α*_ atom^46,49–52^. Although this reactant molecule interaction may result in the cleavage of the Ga_*α*_–S_*α*_ bond, we presume that the S_*α*_ atom can be stabilised by protonation^52,53^.

We observed that the [4 + 2] cycloaddition of **6** shifted from a slow asynchronous concerted reaction to a fast stepwise reaction after Ga–S cluster coordination (Fig. 5b). The free energy barrier dramatically dropped from 14.7 kcal mol^−1^ in the nonenzymatic reaction to 8.8 kcal mol^−1^ in the rate-limiting reaction step producing **1′**_**int+Ga**_. The distance of the forming C_8_–C_13_ bond increased slightly from 2.03 Å in **TS**_**1′**_ to 2.17 Å in **TS**_**1′int+Ga**_. In the second step, the distance of the forming C_7_–C_16_ bond decreased from 2.83 Å in **TS**_**1′**_ to 2.38 Å in **TS**_**1′+Ga,**_ and the free energy barrier is only 4.7 kcal mol^−1^. Compared to the other cycloaddition pathways forming the stereoisomers **7** + **Ga,** **8** + **Ga**, and **9** + **Ga**, Fds with the Lewis acidic Ga(Fe)–S cluster accelerated the TS-pathway with the lowest activation energy barrier and exclusively produced **1′** + **Ga** as in the nonenzymatic reaction (Supplementary Figs. 27a, 30). This significant rate acceleration has also recently been reported in other Lewis acid-catalysed stepwise [4 + 2] cycloadditions, such as the cationic iron (III) porphyrin-catalysed reaction between inactivated aldehydes with simple dienes^54^ and artificial zinc metalloenzyme-catalysed abiological reaction between azachalcone with 3-vinylindole^41^. Regarding the tandem Michael addition of **1′** + **Ga**, a significant decrease in the free energy barrier from 28.6 to 16.1 kcal mol^−1^ occurred with the coordination of the Ga–S cluster, followed by spontaneous tautomerisation to give the final product **1** (Fig. 5b).

Taken together, our experimental results from both native Fds and GaFd and DFT calculations using the Ga-substituted [2Ga–2S + H](Cys)_4_-L model indicate a significant lowering of the relevant TS barriers for the [4 + 2] cycloaddition and Michael addition reactions by the Lewis acidic Fe(Ga)–S clusters. Notwithstanding, it remains an open question how the bridging S_*α*_ atom can be modified allowing an open site for the binding of reactant molecule to the Ga atom^53,55^. In addition, a more detailed comparison of the Ga-substituted and native [2Fe–2S](Cys)_4_, [3Fe–4S](Cys)_3_, and [4Fe–4S](Cys)_4_ Fds and the mechanistic study including the effects of redox states and DFT modelling will be our future work.

### Substrate scope screening for the Fds-catalysed [4 + 2] cycloaddition

Taking into account the ubiquity of Fds in almost all living organisms, the absence of an activity site cavity for substrate selection, and the Lewis acidity of the Fe–S cluster cofactor to lower free energy barriers, we sought to investigate the possible application of Fds as versatile biocatalysts. Previously, our group has reported the Fsa2-family enzymes controlling the stereospecific [4 + 2] cycloadditions of the linear polyenoyltetramic acid **10** to form decalins **11** by Phm7 and **12** by Fsa2 in the active site cavity through two β-barrel domains (Supplementary Fig. 2), and the nonenzymatic conversion of **10** into **13** through a TS-pathway that has the lowest activation energy barrier^56,57^ (Supplementary Fig. 31a). We propose that the Fe–S cluster may form a complex with the carbonyl oxygen atom adjacent to the dienophile to function as efficient Lewis acid^3^. This hypothesis was verified by an in vitro reaction in which the [3Fe–4S] VtlF dose-dependently accelerated the cycloaddition of **10** into **13** (Supplementary Fig. 31b), confirming again the potential of Lewis acidic Fds as versatile biocatalysts for [4 + 2] cycloadditions.

In summary, we have established the mysterious biosynthetic strategy for polycyclic verticilactams, confirmed VtlG as a cytochrome P450 monooxygenase in post-PKS modification, and identified the bifunctional role of [3Fe–4S] VtlF as an Fd in electron transfer and an efficient [4 + 2] cyclase. This Fds-catalysed intramolecular [4 + 2] cycloaddition was initially discovered using spinach [2Fe–2S] Fd but later verified using [3Fe–4S] VtlF and its homologues from mirilactams, sceliphrolactam, and tripartilactam (niizalactam C) gene clusters, suggesting that these actinobacterial Fds may have equivalent roles. We have also experimentally confirmed the Lewis acid activity of Fe–S clusters by Ga-substitution of a cyanobacterial *Synechocystis* [2Fe–2S] Fd with the Lewis acidic but redox-independent [2Ga–2S] cluster. This study is the representative report of a natural enzyme utilising efficient Lewis acid to catalyse [4 + 2] cycloaddition in natural product biosynthesis and stand in contrast with the known enzymes which use the substrate-trapping manner in active site cavity. Furthermore, the DFT calculations reveal that the [4 + 2] cycloaddition shifts from a slow concerted reaction to a fast stepwise reaction. The tandem Michael addition significantly decreases the free energy barrier after Ga–S cluster coordination. Therefore, we propose that the 60-year-old Fds could represent prospective starting scaffolds for the design of versatile organic Lewis acid catalysts for [4 + 2] cycloadditions.

## Methods

### General

Strains, plasmids, and oligonucleotides used in this study are listed in Supplementary Tables 2, 3. Molecular biology experiments were conducted following the manufacturer’s instructions. Oligonucleotides for the polymerase chain reaction (PCR) were purchased from Eurofins Genomics (Tokyo, Japan). PCR amplification was performed using PrimeSTAR^TM^ HS DNA polymerase (Takara Bio Inc., Shiga, Japan) or KOD FX DNA polymerase (TOYOBO CO., LTD., Osaka, Japan). PCR products were purified using a QIAquick PCR Purification Kit (QIAGEN GmbH, Hilden, Germany). Restriction enzymes were purchased from New England Biolabs (Ipswich, MA, USA). DNA ligation was performed using DNA Ligation Kit Ver. 2.1 (Takara Bio Inc., Shiga, Japan). Commercial spinach Fd, spinach FNR, and NADPH (tetrasodium salt) were purchased from Sigma-Aldrich Co. (St. Louis, MO, USA). Synthetic DNAs (Supplementary Table 4) for protein expression of Fds were obtained from Eurofins Genomics (Tokyo, Japan). The Q5 Site-Directed Mutagenesis Kit was purchased from New England Biolabs (Ipswich, MA, USA). Lysozyme from chicken egg white was purchased from Sigma-Aldrich Co. (St. Louis, MO, USA). TurboNuclease was purchased from Accelagen Inc. (San Diego, CA, USA). Precision Plus Protein standards were purchased from Bio-Rad Laboratories (Hercules, CA, USA).

All chemicals were commercially obtained and used without further purification. Unless specifically mentioned, the LC–MS analysis was performed on a Waters ACQUITY UPLC H-Class System equipped with ACQUITY QDa Detector (Waters, Milford, MA, USA) and an AB Sciex API3200 system using ESI probe (AB Sciex, Framingham, MA, USA), under control of Empower 3 or Empower 2 for UPLC and Analyst 1.5.1 for API3200, on a Waters ACQUITY UPLC BEH C_18_ Column (2.1 mm i.d. × 50 mm, 1.7 µm). Medium-pressure liquid chromatography (MPLC) was performed on a CombiFlash companion personal flash chromatography system (Teledyne ISCO, Lincoln, NE, USA) equipped with a RediSep C_18_ column (80 g). Preparative HPLC analysis was performed on a Waters 600E pump system using a SenshuPak PEGASIL ODS column (20 mm i.d. × 250 mm or 10 mm i.d. × 250 mm, 5 µm) (Senshu Scientific Co., Ltd, Tokyo, Japan). UV–vis spectrum, optical rotations, and IR spectrum were recorded using a JASCO V-630 BIO spectrophotometer (JASCO International, Tokyo, Japan), a HORIBA SEPA-300 high-sensitive polarimeter (HORIBA, Kyoto, Japan), and a HORIBA FT-720 spectrometer with a DuraSampl IR II ATR instrument under control of FT-IR for Windows version 4.07, respectively. HR-ESI-TOF-MS analysis was performed using a SYNAPT G2 Mass Spectrometer under control of MassLynx V4.1. ^1^H-NMR (at 500 MHz) and ^13^C-NMR (at 125 MHz) data were obtained on a JEOL ECA-500 FT-NMR spectrometer under control of Delta ver. 5.0.4 (JEOL, Tokyo, Japan). Chemical shifts were reported in ppm, referencing corresponding solvent signals (*δ*_H_ 1.94 and *δ*_C_ 1.39 for acetonitrile-*d*_3_).

### In-frame deletion of *vtlG* gene, transformation, and metabolites profiling in *S. avermitilis* SUKA17

To perform in-frame deletion of the *vtlG* gene in pKU503*vtl*, a PCR targeting and λ-red recombination-based gene replacement approach was used. Plasmid pKD13::*aac(3)IV* was a template to PCR-amplify the FRT-flanked *aac(3)IV* gene cassette, which contained upstream and downstream homologous arms of the *vtlG* gene, using primer set pKD13-Apr-Fwd and pKD13-Apr-Rev. The PCR products and the plasmid pKU503*vtl* were transformed into *E. coli* BW25113/pKD46 to replace the *vtlG* gene with *aac(3)IV* gene. The resultant recombinant plasmid was further transformed into *E. coli* XL1-Blue MRF’ strain by electroporation to eliminate the *aac(3)IV* gene, giving the pKU503*vtl*::Δ*vtlG*. Successful deletion of the *vtlG* gene was confirmed by PCR. After transformation into *E. coli* GM2929 *hsdS*::Tn10 by electroporation, the unmethylated form of pKU503*vtl*::Δ*vtlG* plasmid was isolated and subsequently introduced into the *S. avermitilis* SUKA17 host via polyethene glycol-assisted protoplast transformation^58^. The pKU492*aac(3)IV*-*sav2794p*-*vtlR* plasmid containing a LuxR-family transcriptional regulator *vtlR* gene under the control of *sav2794* promoter^15^ was also introduced into the same strain. The generated *S. avermitilis* SUKA17/pKU503*vtl*::Δ*vtlG*/pKU492*aac(3)IV*-*sav2794p*-*vtlR* (designated *vtlG* disruptant) was subjected to metabolites profiling.

Metabolites of the *vtlG* disruptant were profiled following a similar method to our previous report^15^. In brief, the *vtlG* disruptant was pre-cultured in 10 mL of SK2 medium with neomycin (final 0.2 µg/mL) and apramycin (final 0.2 µg/mL) at 28 °C with rotary shaking at 250 rpm for 3 days. Subsequently, 1 mL of the pre-culture was inoculated into a 500 mL cylindrical flask containing 70 mL of 0.3× BPS medium and cultured at 28 °C with rotary shaking at 150 rpm for 5 days. The culture broth was mixed with an equivalent volume of acetone, filtered under reduced pressure to remove mycelia, and evaporated *in vacuo* to remove acetone. The remaining aqueous layer was extracted twice with an equivalent ethyl acetate (EtOAc) volume, dried in vacuo, and re-dissolved in methanol (MeOH) for UPLC analysis.

### Isolation of compound 4

Large-scale fermentation (7 L) of the *vtlG* disruptant was conducted to isolate compound **4** for enzymatic reactions. The *vtlG* disruptant was pre-cultured in 9 test tubes, each containing 10 mL SK2 medium with neomycin (final 0.2 µg/mL) and apramycin (final 0.2 µg/mL) at 28 °C with rotary shaking at 250 rpm for 3 days. Subsequently, 0.7 mL of the pre-culture was inoculated into 100 cylindrical flasks (500 mL) containing 70 mL of 0.3× BPS medium and cultured at 28 °C with rotary shaking at 150 rpm for 8 days. The culture broth was treated with acetone following a similar procedure described above. The remaining aqueous layer was extracted twice with an equivalent volume of EtOAc. The EtOAc extract was evaporated with a small amount of silica gel resin. The extract–resin mixture was then fractionated with silica gel column chromatography (65 i.d. × 110 mm) using a stepwise solvent system of CHCl_3_:MeOH (100:0, 98:2, 95:5, 90:10, 0:100). The target fraction (CHCl_3_:MeOH; 95:5) was subsequently fractionated by ODS chromatography (40 i.d. × 60 mm) using a stepwise solvent system of H_2_O:MeOH (50:50, 40:60, 30:70, 20:80, 0:100). The 30:70 fraction was purified again under the same condition. Finally, the target fractions (H_2_O:MeOH; 20:80 and 0:100) were purified with preparative HPLC using an isocratic solvent system of 40% acetonitrile (CH_3_CN) with 0.015% formic acid at 6 mL/min. The target peak was collected to give 0.6 mg of **4** as a yellow powder.

### Isolation of compound 5

Large-scale fermentation (36 L) of the *vtlG* disruptant was conducted to isolate compound **5** for structure determination. The *vtlG* disruptant was pre-cultured in a test tube containing 10 mL SK2 medium with neomycin (final 0.2 µg/mL) and apramycin (final 0.2 µg/mL) at 28 °C with rotary shaking at 250 rpm for 2 days. Next, 1 mL pre-culture was inoculated into four cylindrical flasks (500 mL) containing 70 mL of 0.3× BPS medium and cultured at 28 °C with rotary shaking at 150 rpm for 2 days to make the seed culture. Subsequently, 3 mL of the seed culture was inoculated into 500 mL cylindrical flasks containing 70 mL of 0.3× BPS medium and cultured at 28 °C with rotary shaking at 150 rpm for 4 days. The culture broth was centrifuged at 3500 × *g* for 10 min to separate the supernatant and mycelia. The supernatant was extracted thrice with a half volume of EtOAc. The mycelia were resuspended in 1 L of distilled water, mixed with 2 L of acetone, and stirred for 15 h at room temperature. The mycelia–acetone mixture was filtered to remove mycelia, evaporated to remove acetone, and then extracted thrice with a half volume of EtOAc. After that, all the EtOAc layers were combined and added with sodium sulfate anhydrous to remove water traces. This EtOAc layer was evaporated to yield ~3 g of brownish oily crude extract. Next, the crude extract was re-dissolved with a small amount of MeOH, applied to a Sephadex LH-20 column (40 mm i. d. × 250 mm), and eluted with MeOH to give 20 fractions. The 9th (1 g) and 10th (317 mg) fractions were further separated with MPLC using a linear gradient of 20–100% CH_3_CN in 100 column volume at 60 mL/min to give 120 fractions. The MPLC fractions 22–27 were purified with preparative HPLC using an isocratic solvent system of 72% CH_3_CN at a 5 mL/min flow rate. The target peak was collected to give 1 mg of **5** as colourless amorphous.

### In vitro P450 VtlG assay using 4 and 5

Time-dependent conversion of compound **4** as a substrate was conducted at 30 °C in a reaction mixture (600 μL) containing 50 mM Tris-HCl (pH 7.5), 1 μM **4** (dissolved in DMSO), 0.5 μM purified VtlG, 0.1 mg/mL commercial spinach Fd, and 1 unit/mL spinach FNR. Compound **4** was quantified according to a standard curve generated based on the peak area at 350 nm using UPLC. After pre-incubation at 30 °C for 2 min, the reaction was initiated by rapidly adding 100 μM NADPH. At each desired time point (1, 30, 60 min), a 200 μL aliquot of the reaction mixture was taken, quenched with 5 μL acetic acid, and extracted twice with 400 μL EtOAc. An appropriate DMSO volume (20 μL) was added before evaporating EtOAc with nitrogen gas. The remaining DMSO fraction (15 μL) was used for UPLC analysis with a linear gradient of 10–100% CH_3_CN with 0.1% formic acid in 3 min at 0.5 mL/min.

The conversion of compound **5** was also examined at 30 °C in a 200 μL reaction mixture containing 50 mM Tris-HCl (pH 7.5), 1 μM **5** (dissolved in DMSO), 0.5 μM purified VtlG, 0.1 mg/mL commercial spinach Fd, and 1 unit/mL spinach FNR. After pre-incubation at 30 °C for 2 min, the reaction was initiated by adding 1 mM NADPH rapidly. After 30 min incubation, the reaction mixture was quenched with 5 μL acetic acid and extracted twice using 400 μL EtOAc. An appropriate DMSO volume (20 μL) was added before evaporating EtOAc with nitrogen gas. The remaining DMSO fraction (15 μL) was used for UPLC analysis with a linear gradient of 10–100% CH_3_CN with 0.1% formic acid in 3 min at 0.5 mL/min.

### Kinetic analysis of P450 VtlG against 4

To determine the kinetic parameters of the VtlG-catalysed mono-hydroxylation reaction against **4**, the reaction mixture (200 μL) contained 50 mM Tris-HCl (pH 7.5), 0.25–2.5 μM **4** (dissolved in DMSO), 5 nM purified VtlG, 0.1 mg/mL commercial spinach Fd, and 1 unit/mL spinach FNR. After pre-incubation at 30 °C for 2 min, the reaction was started by rapidly adding 1 mM NADPH and incubating for another 2 min. The peak area at 350 nm by UPLC was used to quantify the reaction product of **4**. The *K*_m_ and *k*_cat_ values were obtained by nonlinear curve fitting using the Michaelis–Menten equation in SigmaPlot 12 software (Systat Software, Inc., San Jose, CA, USA).

### Preparation of 6 by VtlG reaction

Large-scale VtlG reaction to prepare **6** was conducted at 30 °C in a 1 mL reaction mixture containing 50 mM Tris-HCl (pH 7.5), 5 μM **4** (dissolved in DMSO), 0.5 μM purified VtlG, 0.1 mg/mL spinach Fd, and 1 unit/mL spinach FNR. After pre-incubation at 30 °C for 2 min, the reaction was initiated by adding 10 mM NADPH to an excess amount to inhibit [4 + 2] cycloaddition reaction. After incubation for 5 min, the reaction mixture was extracted twice with 1 mL EtOAc. An appropriate DMSO volume (100 μL) was added before evaporating EtOAc with nitrogen gas. The remaining DMSO fraction (80 μL) containing the hydroxylated compound **6** was prepared as a substrate for the subsequent [4 + 2] cycloaddition assays. Compound **6** was quantified according to a standard curve generated based on the peak area at 350 nm using UPLC.

### In vitro conversion of 4 and 6 by commercial spinach Fd

Enzyme assays of commercial spinach Fd were conducted at 30 °C in a 200 μL reaction mixture containing 50 mM Tris-HCl (pH 7.5), 1 μM **4** or **6** as reactant (dissolved in DMSO), and 0.5 mg/mL (47 μM) commercial spinach Fd. After pre-incubation at 30 °C for 2 min, the reaction was initiated by adding the reactant. The negative control was the boiled (95 °C, 15 min) spinach Fd. After the reaction, the mixture was extracted twice with 400 μL EtOAc. Then, an appropriate volume (20 μL) of DMSO was added before evaporating EtOAc with nitrogen gas. The remaining DMSO fraction (15 μL) was used for UPLC analysis with a linear gradient of 10–100% CH_3_CN with 0.1% formic acid at 0.5 mL/min in 3 min for the reaction product of **6** and 12 min for the reaction product of **4**.

### Kinetic analysis of spinach Fd against 4

To determine the kinetic parameters of the spinach Fd-catalysed [4 + 2] cycloaddition reaction against **4**, the reaction mixture (200 μL) contained 50 mM Tris-HCl (pH 7.5), 0.25–3 μM **4** (dissolved in DMSO), 24 μM commercial spinach Fd. After pre-incubation at 30 °C for 2 min, the reaction was started by rapid addition of **4** and incubated for another 4 min. Compound **5** was quantified according to a standard curve generated based on the peak area at 280 nm using UPLC. The *K*_m_ and *k*_cat_ values were obtained by nonlinear curve fitting using the Michaelis–Menten equation in SigmaPlot 12 software.

### In vitro conversion of 4 and 6 by recombinant Fds

Enzyme assays of each recombinant Fds were conducted at 30 °C in a 200 μL reaction mixture containing 50 mM Tris-HCl (pH 7.5), 1 μM **4** or **6** as reactant (dissolved in DMSO), and 50 μM recombinant Fd. After pre-incubation at 30 °C for 2 min, the reaction was initiated by adding the reactant. After the reaction, the mixture was extracted twice with 400 μL EtOAc. An appropriate DMSO volume (20 μL) was added before evaporating EtOAc with nitrogen gas. The remaining DMSO fraction (15 μL) was used for UPLC analysis with a linear gradient of 10–100% CH_3_CN with 0.1% formic acid at 0.5 mL/min in 3 min for the reaction product of **6** and 12 min for the reaction product of **4**.

### Kinetic analysis of MirFd against 4

To determine the kinetic parameters of the MirFd-catalysed [4 + 2] cycloaddition reaction against **4**, the reaction mixture (200 μL) contained 50 mM Tris-HCl (pH 7.5), 0.5–3 μM **4** (dissolved in DMSO), and 10 μM recombinant MirFd. After pre-incubation at 30 °C for 2 min, the reaction was initiated by rapidly adding **4** and incubating for another 2 min. Compound **5** was quantified according to a standard curve generated based on peak area at 280 nm using UPLC. The *K*_m_ and *k*_cat_ values were obtained by nonlinear curve fitting using the Michaelis–Menten equation in SigmaPlot 12 software.

### Preparation of apo-Fds

EDTA treatment of spinach Fd and MirFd was performed following a minorly modified method^33^. Briefly, the purified recombinant Fds (1 mg) were boiled at 95 °C in 100 mM EDTA and 50 mM DTT in 1.5 mL Eppendorf tubes. The tubes were inverted until the proteins became colourless. After centrifugation (4 °C; 8000 × *g*; 10 min), the supernatant was further centrifuged with Amicon® Ultra-15 (10 kDa cutoff) centrifugal filter to remove iron atoms, sulfide, and excess EDTA and DTT. Absorption spectra were analysed. The decrease of absorption maxima at 463, 420, and 325 mm for spinach Fd and 418 mm for MirFd indicated the Fe–S cluster removal. The concentration of spinach Fd and MirFd apo-forms was determined using Bradford assay.

PCR-based site-directed mutagenesis was performed following the Q5 Site-Directed Mutagenesis Kit protocol to obtain apo-form spinach Fd and MirFd mutants, which cannot bind the Fe–S cluster. For spinach Fd, plasmid pET-28b(+)::*spiFd* was a template while primer set SpiFd_C40A_Fwd and SpiFd_C40A_Rev was used for C40A mutation. For MirFd, pET-28b(+)::*mirFd* was a template while primer set MirFd_C19A_Fwd and MirFd_C19A_Rev was used for C19A. The resultant mutant plasmids were confirmed by Sanger sequencing and transformed into *E. coli* BL21(DE3) for protein expression.

### Preparation of reduced Fds by sodium dithionite

The aerobically purified recombinant Fds were reduced by adding freshly prepared sodium dithionite in 1.5 mL Eppendorf tubes to a final concentration of 1 mM^21,22^. The tubes were carefully inverted severally until the proteins became almost colourless. Then, UV absorption spectra analyses were performed. The decrease of absorption peaks at 463, 420, and 325 mm for spinach Fd and 418 mm for MirFd indicated the Fe–S cluster reduction.

### Preparation of GaFd and [^15^N]-labelled GaFd from *Synechocystis*

Recombinant proteins of the native Fd (SynFd) and [^15^N]-labelled SynFd from *Synechocystis* sp. PCC 6803 were expressed and purified from *E. coli* BL21(DE3) cells cultured with Luria–Bertani medium and M9 minimal medium (containing ^15^NH_4_Cl as the sole nitrogen source), respectively^35^. The detailed protein purification is described in the Supplementary Methods. Ga-substitution of the SynFd and [^15^N]-labelled SynFd were performed^35^. Briefly, 5 mg SynFd and 15 mg [^15^N]-labelled SynFd were denatured with 6 M HCl to a final concentration of 1 M. Subsequently, we rinsed the pellets with Milli-Q water and resuspended them in 100 mM Tris-HCl (pH 8.0) buffer, respectively. The above steps were repeated thrice to remove iron atoms completely. The final denatured Fd was resuspended in 100 mM Tris-HCl (pH 8.0) buffer containing 10 mM DTT and 6 M guanidine hydrochloride. Next, the apo-forms of SynFd and [^15^N]-labelled SynFd were refolded at 4 °C by dilution into 20 mM Tris-HCl (pH 8.0), 2 mM DTT, 2 mM GaCl_3_, and 2 mM Na_2_S. After overnight incubation, the GaFd and [^15^N]-labelled GaFd were loaded onto a HiTrap Q HP anion exchange column, eluted with a linear NaCl gradient (0–1 M NaCl), and concentrated by ultrafiltration. The purity of the GaFd and [^15^N]-labelled GaFd were confirmed using SDS–PAGE analysis, and the concentration was calculated with a molar extinction coefficient (*ε*_280_ = 170.2 mM^−1^ cm^−1^).

### NMR analysis of [^15^N]-labelled GaFd with ligand 4

The ^1^H–^15^N HSQC spectra were acquired on Avance III 950 US^2^ spectrometer equipped with a TCI cryogenic probe (Bruker Biospin, Germany) at 950 MHz following a minorly modified method^35^. The NMR measurement was performed with 10 μM [^15^N]-labelled GaFd and 10 μM ligand **4** (dissolved in DMSO) in 20 mM sodium phosphate buffer (pH 6.5) containing 50 mM NaCl at 277 and 298 K. The peaks were assigned with the chemical shifts of BMRB entry 16024 by NMRFAM-SPARKY. After measurement, [4 + 2] cycloaddition of compound **4** in the NMR tube was analysed using UPLC as described above.

### Computational methods

All the calculations were carried out with the Gaussian 16 (revision B.01) program package^59^. The molecular structures and harmonic vibrational frequencies were obtained using the hybrid density functional method based on the M06-2X functional^60^. We used the SDD^61^ and 6-311 + G** ^62^ basis set. The self-consistent reaction field (SCRF) method based on the conductor-like polarisable continuum model (CPCM)^63,64^ was employed to evaluate the solvent reaction field (water; *ε* = 78.39). Geometry optimisation and vibrational analysis were performed at the same level. All stationary points were optimised without symmetry assumptions and characterised by normal coordinate analysis at the same level of theory (number of imaginary frequencies, 0 for minima and 1 for TSs). The intrinsic reaction coordinate method^65,66^ was used to track minimum energy paths from transition structures to the corresponding local minima.

### In vitro conversion of linear polyenoyltetramic acid 10 by VtlF

The linear polyenoyltetramic acid **10** and authentic standards of compounds **11**–**13** were prepared^56,57^. The Δ*phm7* mutant of *Pyrenochaetopsis* sp. RK10-F058 was cultured in CYA medium at 28 °C with rotary shaking at 150 rpm for 3 days. The mycelia were collected, washed, and frozen at −80 °C. An in vitro enzyme assay of **10** was performed following a minorly modified method^56,57^. The mycelial powder was resuspended in DMSO, centrifuged at 4 °C; 20,000 × *g*; 10 min, and the **10**-saturated supernatant was used as a substrate for enzyme assay. Dose-dependent assay of VtlF was conducted in a 100 μL reaction mixture containing 50 mM Tris-HCl (pH 7.5), 10 μL **10**-saturated supernatant, and 0, 0.2, 0.4, 0.8 mg/mL recombinant VtlF. After incubation on ice for 5 min, the reaction was quenched with 5 μl ice-cold acetic acid and centrifuged.

The UPLC analysis was performed on a Waters ACQUITY UPLC BEH C_18_ Column (2.1 mm i.d. × 100 mm, 1.7 µm) at 0.25 mL/min flow rate^56,57^. After the injection of 3 μL reaction product into the column equilibrated with 5% CH_3_CN with 0.05% formic acid, the column was developed with a linear gradient of 5–60% CH_3_CN with 0.05% formic acid over 1 min and 60–100% CH_3_CN with 0.05% formic acid over 4 min. Finally, the column was isocratically eluted with 100% CH_3_CN with 0.05% formic acid for 6 min.

### Reporting summary

Further information on research design is available in the Nature Portfolio Reporting Summary linked to this article.

### Supplementary information

Supplementary Information

Peer Review File

Reporting Summary

### Source data

Source Data

## Data Availability

The nucleotide sequence of the vtl cluster has been deposited in the NCBI database by previous study under the accession number LC523631. The data that support the findings of this study are presented in the article and Supplementary Information. Other relevant data are available from the corresponding authors upon request. Source data are provided with this paper.
