# Supplementary material for: Iron-sulphur protein catalysed [4+2] cycloadditions in natural product biosynthesis
Source: Nat Commun. 2024 Jul 10;15:5779. doi: 10.1038/s41467-024-50142-1 (PMC11236979; doi:10.1038/s41467-024-50142-1)
Supplement: Supplementary file 1 — Supplementary Information [file 41467_2024_50142_MOESM1_ESM.pdf]

Supplementary Information

Iron-sulphur protein catalysed [4+2] cycloadditions in natural product biosynthesis

Yu Zheng[1,†], Katsuyuki Sakai[1,†], Kohei Watanabe[2,†], Hiroshi Takagi[1], Yumi Sato-Shiozaki[1], Yuko Misumi[3], Yohei Miyanoiri[3], Genji Kurisu[3], Toshihiko Nogawa[4], Ryo Takita[2,5], Shunji Takahashi[1,*]

[1] Natural Product Biosynthesis Research Unit, RIKEN Center for Sustainable Resource Science, Saitama, 351-0198, Japan

[2] Graduate School of Pharmaceutical Sciences, The University of Tokyo, Tokyo, 113-0033, Japan

[3] Institute for Protein Research, Osaka University, Osaka, 565-0871, Japan

[4] Molecular Structure Characterization Unit, RIKEN Center for Sustainable Resource Science, Saitama, 351-0198, Japan

[5] Graduate School of Pharmaceutical Sciences, University of Shizuoka, Shizuoka, 422-8526 Japan

* Correspondence: shunjitaka@riken.jp

[†] Equal contribution to this work.

Table of Contents

Supplementary Methods ... 4
Construction of pKD13::*aac(3)IV* plasmid ... 4
Structure determination of 5 ... 4
Cloning, expression, and purification of VtlG ... 5
Protein expression and purification of recombinant Fds ... 7
Protein expression and purification of apo-spinach Fd and apo-MirFd mutants ... 8
Protein purification of *Synechocystis* sp. PCC 6803 Fd (SynFd) and [^{15}N]-labelled SynFd ... 8
Supplementary Figures ... 10
Supplementary Fig. 1 ... 10
Supplementary Fig. 2 ... 11
Supplementary Fig. 3 ... 12
Supplementary Fig. 4 ... 13
Supplementary Fig. 5 ... 14
Supplementary Fig. 6 ... 15
Supplementary Fig. 7 ... 16
Supplementary Fig. 8 ... 17
Supplementary Fig. 9 ... 18
Supplementary Fig. 10 ... 19
Supplementary Fig. 11 ... 20
Supplementary Fig. 12 ... 21
Supplementary Fig. 13 ... 22
Supplementary Fig. 14 ... 23
Supplementary Fig. 15 ... 24
Supplementary Fig. 16 ... 25

Supplementary Fig. 17 26
Supplementary Fig. 18 27
Supplementary Fig. 19 28
Supplementary Fig. 20 29
Supplementary Fig. 21 30
Supplementary Fig. 22 31
Supplementary Fig. 23 32
Supplementary Fig. 24 33
Supplementary Fig. 25 34
Supplementary Fig. 26 36
Supplementary Fig. 27 37
Supplementary Fig. 28 38
Supplementary Fig. 29 39
Supplementary Fig. 30 40
Supplementary Fig. 31 41
Supplementary Tables 42
Supplementary Table 1 42
Supplementary Table 2 43
Supplementary Table 3 45
Supplementary Table 4 46
Supplementary References 47

Supplementary Methods

Construction of pKD13::*aac(3)IV* plasmid

To construct pKD13::*aac(3)IV* plasmid, the FRT-flanked *aph(3')II* gene in plasmid pKD13 was replaced with the *aac(3)IV* gene via λ-red recombination. A DNA fragment including the *aac(3)IV* gene and 40 bp homologous arms corresponding to the *aph(3')II* gene was PCR-amplified from pKU492*aac(3)IV* using primer set pKU492-Apr-Fwd and pKU492-Apr-Rev. The PCR products and the plasmid pKD13 were transformed into *Escherichia coli* BW25113/pKD46 for λ-red recombination.

Structure determination of 5

The molecular formula of **5** was determined as $C_{25}H_{33}NO_4$ using HR-ESI-TOF-MS (found: *m*/*z* 412.2491 $[M + H]^+$, calculated for $C_{25}H_{34}NO_5$ 412.2488), requiring ten degrees of unsaturation. The ^{1}H NMR spectrum showed two methyl signals and nine olefin signals (Table S1). The ^{13}C NMR spectrum possessed 25 carbon signals, including two methyls, four methylenes, and 16 methines that bore two oxygenated and nine olefin signals, and three quaternary carbons, including an olefin carbon and two carbonyl carbons at *δ* 165.9 and 197.1, which were verified by the ^{13}C DEPT and HSQC spectra data. These observations suggested that **5** was a tricyclic compound with five double bonds and two ketones. The 2D NMR spectra were measured to determine the structure. The connections between protons and carbons were determined by correlations in the HSQC spectrum. The DQF-COSY and HSQC-TOCSY spectra revealed the connectivities from C-5 (δ_C 134.1) to C-16 (δ_C 52.8), and confirmed the presence of an octalin skeleton with two hydroxyl groups at C-10 and C-12, and verticilactams. The connectivities from C-18 (δ_C 124.9) to NH (δ_H 6.92) with a methyl group (C-26, δ_C 18.2) at the C-23 (δ_C 38.6) position were also revealed through the DQF-COSY and HSQC-TOCSY spectra. A singlet methyl signal at δ_H 1.61 (C-25, δ_C 13.2) was attached at C-17 by the correlations with C-16, C-17, and C-18 in the HMBC spectrum, and it also indicated the connectivities from C-16 to C-18. This was confirmed by a long-range correlation from H-18 (δ_H 6.03) to C-16. The H-5 (δ_H 5.77) signal showed an HMBC

correlation with a ketone signal at δ_C 197.2, which suggested that it was assigned C-4. The H_2-3 (δ_H 3.17 and 3.74) signal correlated to C-4 and another carbonyl signal at δ_c 165.9, which revealed an assignment of the carbonyl signal as C-2 and a dicarbonyl moiety from C-2 to C-4. The connectivities from C-2 to C-5 were also confirmed by NOE correlations between H-3 and H-5 (δ_H 5.77) signals in the NOESY spectrum. An NOE correlation between NH and H_2-3 signals was observed, indicating an amide bond supported by the relatively high-field chemical shift value of C-2. Therefore, the planar structure of **1** was determined, as shown in Supplementary Fig. 18. The geometries at Δ^5 and Δ^{21} were assigned *E*-configuration by the large coupling constants of 16.1 and 14.9 Hz in the ^{1}H NMR spectrum, respectively. On the other hand, the geometry at Δ^{19} was assigned *Z*-configuration by the small coupling constant of 10.9 Hz. The observed C-25 with the typical high-field chemical shift value of δ 13.2 suggested that the Δ^{17} was assigned an *E*-configuration. The relative stereochemistry of the octalin skeleton was assigned by NOESY correlations, as presented in Supplementary Fig. 16. H-8 correlated with H-13 and H-16, suggesting that they were on the same side and the octalin skeleton had a *cis*-configuration. Furthermore, 10-OH correlated with H-7, indicating that they were on the same side, and the hydroxyl group was axial. The 12-OH was assigned the same side as 10-OH by the NOESY correlations of H-12 with H-13 and H-14. It was confirmed by the NOESY correlations of H-12 with both H-11s, which also confirmed that the hydroxyl group was axial. Thus, part of the relative stereochemistry around the octalin skeleton in **5** was assigned, as shown in Supplementary Fig. 18.

Compound **5**: colourless powder; $[\alpha]_{589}^{25.0}$ (*c*=0.036, MeCN) -552.7°; UV (MeCN) λ_{max} (log ε) 238 (4.39), 280 (4.40); IR λ_{max} (ATR) cm^{-1} 3348, 2920, 2854, 1736, 1716, 1653, 1647, 1635, 1558, 1541, 1522, 1508, 1458, 1338, 1095, 987, 944, 849, 823, 762; HR-ESI-TOF-MS found: *m/z* 412.2491 $[M + H]^+$, calculated for $C_{25}H_{34}NO_5$ 412.2488; ^{1}H and ^{13}C NMR chemical shifts are summarized in Supplementary Table 1.

Cloning, expression, and purification of VtlG

For gene cloning, a DNA fragment containing the *vtlG* gene was PCR-amplified from pKU503::*vtl* plasmid using primer sets Nde-vtlG-Fwd and Xho-vtlG-Rev. After purifying

the PCR products, the *vtlG* gene was ligated into *Nde*I and *Xho*I sites of the pET-28b(+) vector. The recombinant plasmids were transformed into *E. coli* DH5α. Positive clones were selected using colony PCR. For protein expression, the resultant pET-28b(+)::*vtlG* was subsequently transformed into *E. coli* BL21 Star^{TM} (DE3). A single colony was pre-cultured with 5 mL of Terrific broth (TB) medium supplemented with kanamycin (final 50 µg/mL) at 37°C, with 150 rpm rotational agitation overnight. Subsequently, pre-culture was inoculated into 2 L of TB medium supplemented with kanamycin (final 50 µg/mL) and cultured at 37°C with an initial optical density of 0.1 at 600 nm (OD_{600}). When the OD_{600} value reached 0.5~0.6, gene expression was induced by adding isopropyl β-D-1-thiogalactopyranoside (IPTG) (final 0.5 mM) and supplemented with 5-aminolevulinic acid (final 0.5 mM). The recombinant VtlG was expressed at 20°C, with 150 rpm rotational agitation overnight.

Protein purification of VtlG was conducted[1]. Briefly, the BL21(DE3) cells were collected by centrifugation (4°C; 5,000 × g; 10 min) and resuspended in buffer A (50 mM Tris-HCl (pH 7.5), 500 mM NaCl, 10 mM 2-mercaptoethanol, and 20% (v/v) glycerol) including 0.5 mg/mL of lysozyme from chicken egg white and 500 units of TurboNuclease. The cells were lysed by sonication for 30 × 30 s with a 30 s interval at 4°C using a Bioruptor (BM Equipment Co., Ltd., Tokyo, Japan). Insoluble cell debris was removed via centrifugation (4°C; 10,000 × g; 20 min). The supernatant was applied onto a Ni-NTA agarose column (10 mm i. d. x 20 mm) (QIAGEN GmbH, Hilden, Germany) and washed with buffer B (50 mM Tris-HCl (pH 7.5), 500 mM NaCl, 10 mM 2-mercaptoethanol, and 10% (v/v) glycerol) containing 5 mM imidazole and 0.2% (v/v) Tween 20. The column was further washed with buffer B containing 5 mM imidazole and 40 mM imidazole, respectively. The His_8-tagged VtlG was eluted with buffer B containing 250 mM imidazole and concentrated with Amicon® Ultra-15 (30 kDa cutoff) centrifugal filter (Millipore Corp., Burlington, MA, USA). The purity of the eluted VtlG was confirmed using SDS-PAGE analysis. The concentration of functional VtlG was determined using carbon monoxide (CO) difference spectroscopy and calculated at the extinction coefficient of 91,000 M^{-1} cm^{-1} at 450 nm[2]. The purified VtlG was dialysed against storage buffer (50 mM Tris-HCl (pH 7.5) and 20% (v/v) glycerol) and frozen at -80°C for later use.

Protein expression and purification of recombinant Fds

DNAs of spinach Fd (codon-optimized), VtlF, MirFd, SceC, and TriM (original sequence) were synthesized by Eurofins Genomics and ligated into *Nde*I and *Xho*I sites of pET-28b(+) vector. The constructed plasmids were transformed into *E. coli* DH5α, respectively. Correct clones were confirmed by restriction enzyme digestion. For protein expression, the resultant pET-28b(+)::*spiFd*, pET-28b(+)::*vtlF*, pET-28b(+)::*mirFd*, pET-28b(+)::*sceC*, and pET-28b(+)::*triM*, respectively, were co-transformed with pRKSUF017 plasmid into *E. coli* C41 OverExpress™ (DE3) via electroporation[3]. A single colony was pre-cultured with 5 mL of TB medium supplemented with kanamycin (final 50 μg/mL) and tetracycline (final 10 μg/mL) at 37°C, with 150 rpm rotational agitation overnight. Subsequently, pre-culture was inoculated into 1 L of auto-inducing TB medium (12 g/L tryptone, 24 g/L yeast extract, 0.5 g/L glucose, 2 g/L lactose, 4 ml/L glycerol, 2.312 g/L KH_2PO_4, and 12.541 g/L K_2HPO_4) supplemented with kanamycin (final 50 μg/mL), tetracycline (final 10 μg/mL), ferric ammonium citrate (final 0.2 g/L), and L-cysteine (final 0.15 g/L). The recombinant Fds were expressed at 30°C, with 225 rpm rotational agitation in 24 h.

Protein purification of Fds was conducted following a modified VtlG method. The C41(DE3) cell pellets were collected via centrifugation (4°C; 5,000 × g; 10 min) and resuspended in buffer C (50 mM Tris-HCl (pH 7.5), 500 mM NaCl, 1 mM dithiothreitol (DTT), and 20% (v/v) glycerol), including 0.5 mg/mL of lysozyme from chicken egg white, 500 units of TurboNuclease, and 2% (v/v) Tween 20. The cells were lysed via sonication for 30 × 30 s with 30 s intervals at 4°C using Bioruptor. Insoluble cell debris was removed using centrifugation (4°C; 8,000 × g; 30 min). The supernatant was applied onto a Ni-NTA agarose column and washed with buffer C containing 2% (v/v) Tween 20 and buffer C. The column was further washed with buffer C containing 5 mM imidazole and 40 mM imidazole, respectively. The His_6-tagged Fds were eluted with buffer C containing 250 mM imidazole and concentrated with Amicon® Ultra-15 (10 kDa cutoff) centrifugal filter. The purities of

the recombinant Fds were confirmed using SDS-PAGE analysis, and each protein concentration was determined using Bradford assay. Absorption spectra were measured to confirm the correct assembly of Fe-S clusters in recombinant spinach Fd (absorption peaks at 463, 420, and 325 mm) and actinobacterial Fds (absorption peaks at 418 mm). The purified recombinant Fds were dialysed against storage buffer and frozen at -80°C for enzymatic assay.

Protein expression and purification of apo-spinach Fd and apo-MirFd mutants

The mutant spinach Fd C40A and mutant MirFd C19A were expressed in 1 L of auto-inducing TB medium supplemented with kanamycin (final 50 μg/mL) at 30°C, with 225 rpm rotational agitation in 24 h. Protein purification was performed as described for recombinant Fds. The mutants' purities were confirmed using SDS-PAGE analysis, and each concentration was determined via Bradford assay. The purified proteins were dialysed against storage buffer and frozen at -80°C.

Protein purification of *Synechocystis* sp. PCC 6803 Fd (SynFd) and [^{15}N]-labelled SynFd

For protein purification, BL21(DE3) cell pellets harbouring recombinant SynFd and [^{15}N]-labelled SynFd, respectively, were collected via centrifugation, resuspended in buffer D (50 mM Tris-HCl (pH 7.5), 50 mM NaCl), and disrupted using sonication[4]. After centrifugation, the resulting supernatant was applied onto a Cellfine A-200 anion exchange cellulose column. The native SynFd and [^{15}N]-labelled SynFd samples were eluted with 50 mM Tris-HCl (pH 7.5) buffer containing 500 mM NaCl and dialysed with buffer D. The dialysates were then applied onto a HiTrap Q HP anion exchange column, eluted with a linear NaCl gradient (0-1 M NaCl), and precipitated with ammonium sulphate (50% saturation). Next, the supernatant was loaded onto a HiLoad 26/10 Phenyl Sepharose HP column and eluted with a linear ammonium sulphate gradient (0%-50% saturation). Finally, red-coloured fractions were concentrated via ultrafiltration and further purified with gel filtration

chromatography using a HiLoad 16/600 Superdex 200 pg column. The purity of the recombinant native SynFd and [^{15}N]-labelled SynFd samples were confirmed using SDS-PAGE analysis, and the concentration was calculated with a molar extinction coefficient (ε_{422} = 9.68 mM^{-1} cm^{-1}).

Supplementary Figures

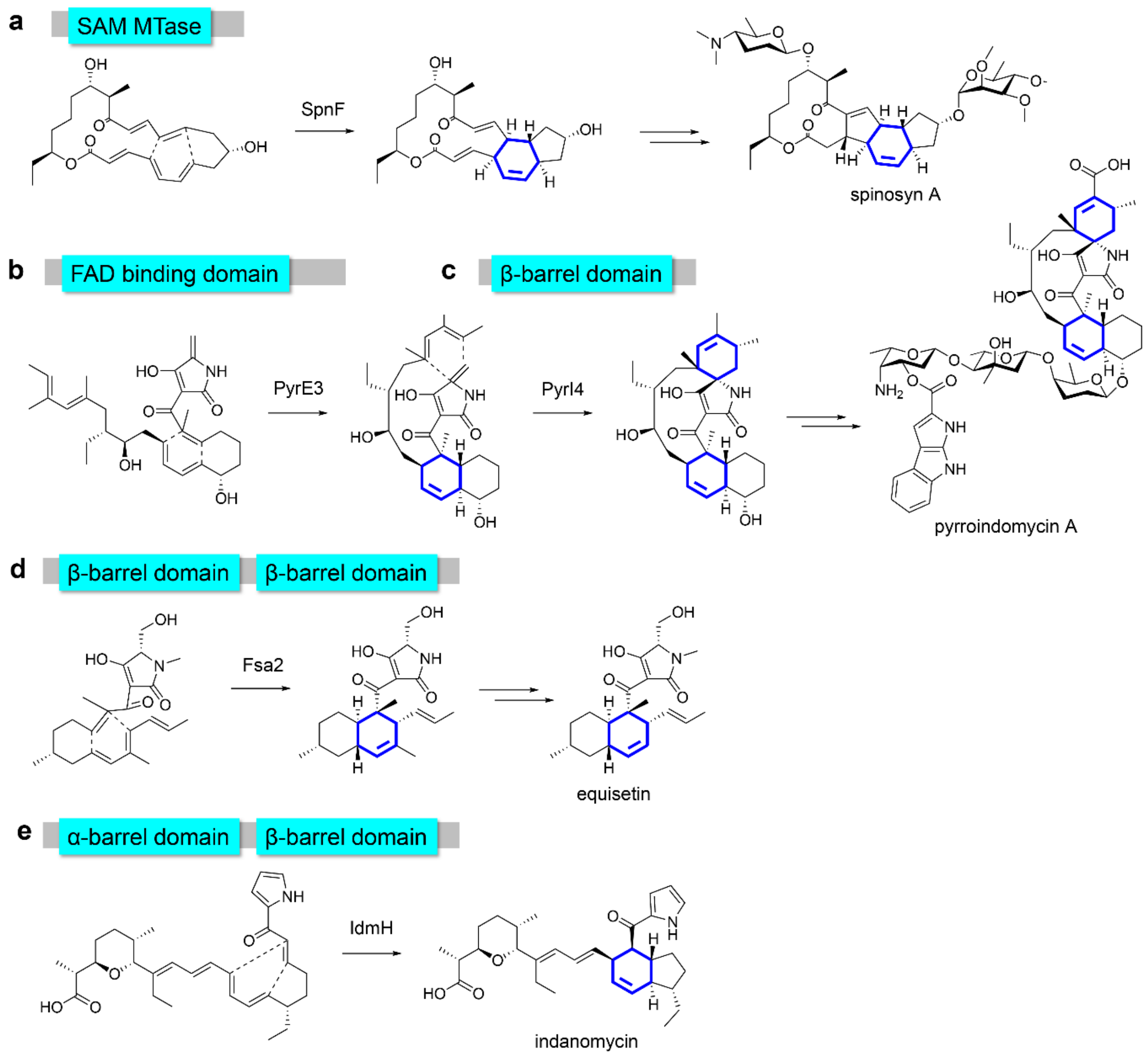

Supplementary Fig. 1 | Representatives of intramolecular [4+2] cycloadditions in natural products biosynthesis. **a**, SpnF catalyses [4+2] cycloaddition in spinosyn A biosynthesis[5]. PyrE3 **(b)** and PyrI4 **(c)** catalyse tandem [4+2] cycloadditions in the biosynthesis of pyrroindomycin A[6]. **d**, Fsa2 catalyses decalin formation in equisetin biosynthesis[7]. **e**, IdmH is involved in indanomycin biosynthesis[8].

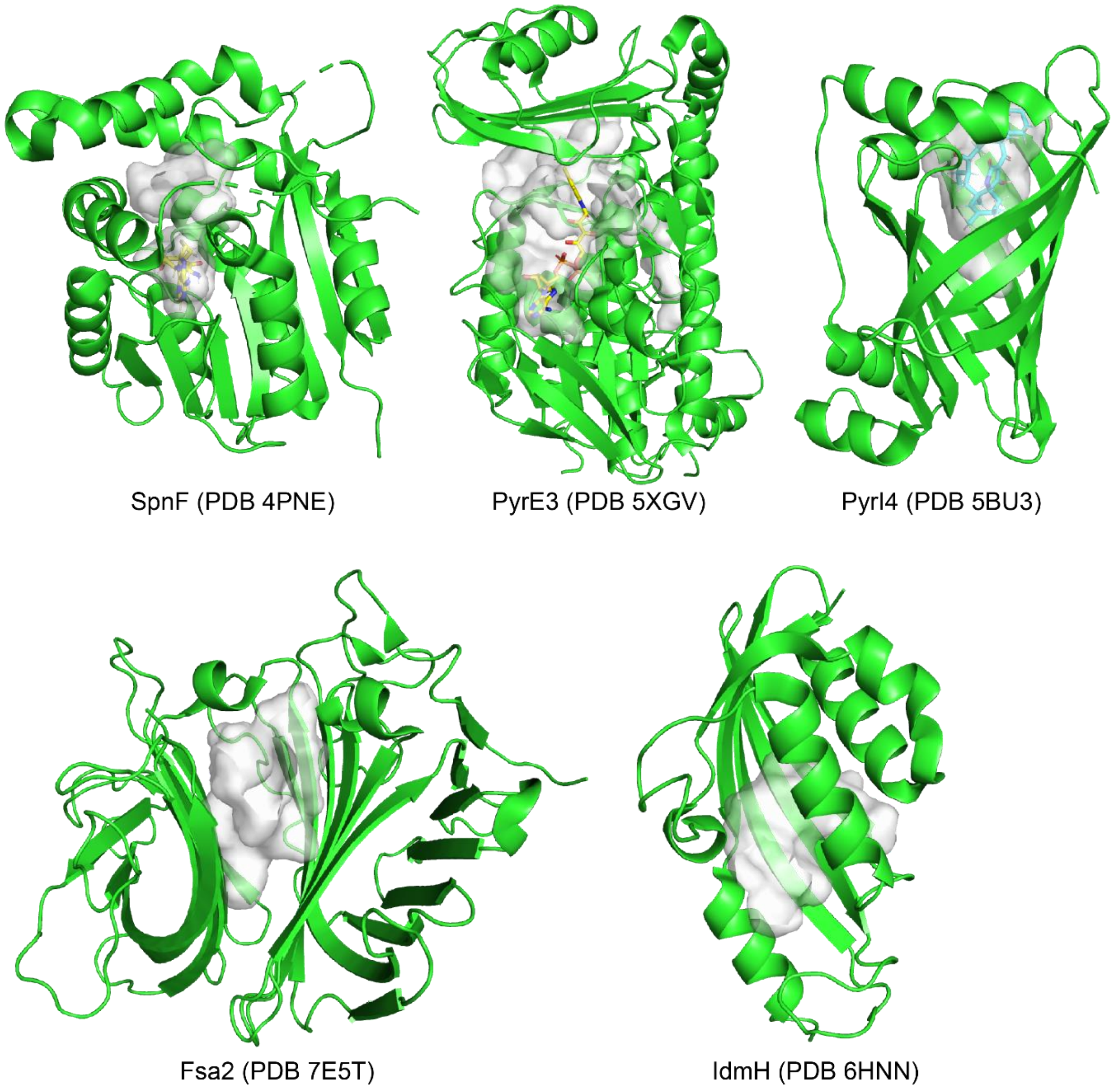

Supplementary Fig. 2 | Crystal structures and active site cavities of microbial-derived [4+2] cyclases. The SAM cofactor in SpnF and FAD cofactor in PyrE3 are shown in sticks in yellow. The [4+2] cycloaddition product of PyrI4 is shown in sticks in cyan.

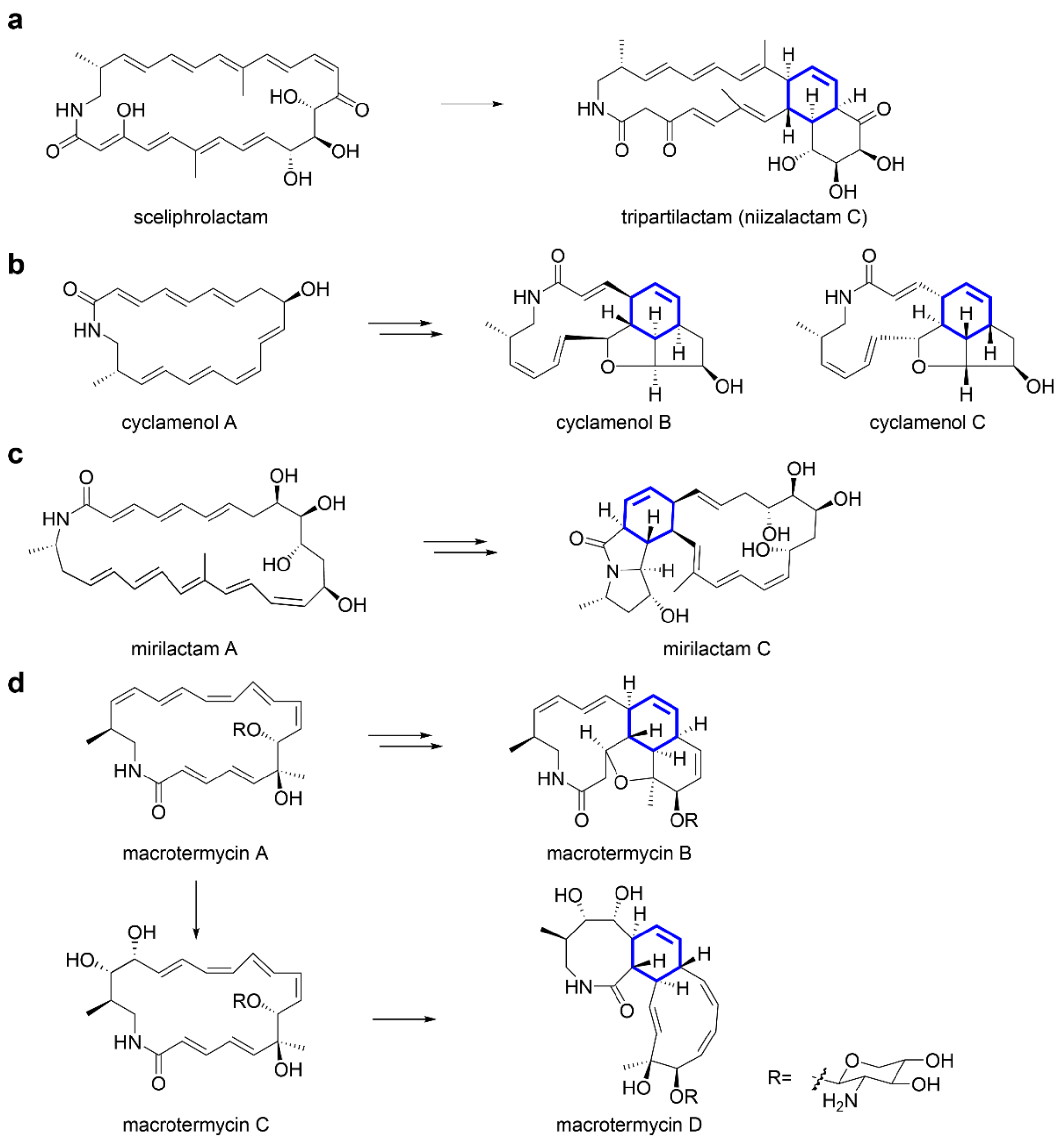

Supplementary Fig. 3 | Polycyclic macrolactams biosynthesised by intramolecular [4+2] cycloadditions. **a**, Sceliphrolactam is converted into tripartilactam after incubation at ambient temperature for 5 days[9]. **b**, Cyclamenols B and C are stereoisomers generated from parent cyclamenol A[10]. **c**, Mirilactam A is speculated to undergo cascade epoxidation and [4+2] cycloaddition to form mirilactam C[11]. **d**, Macrotermycin A is proposed to undergo cascade [4+2] cycloaddition and Micheal addition to generate macrotermycin B[12]. Macrotermycin A can also be hydroxylated to form macrotermycin C, which undergoes [4+2] cycloaddition to form macrotermycin D[12].

a

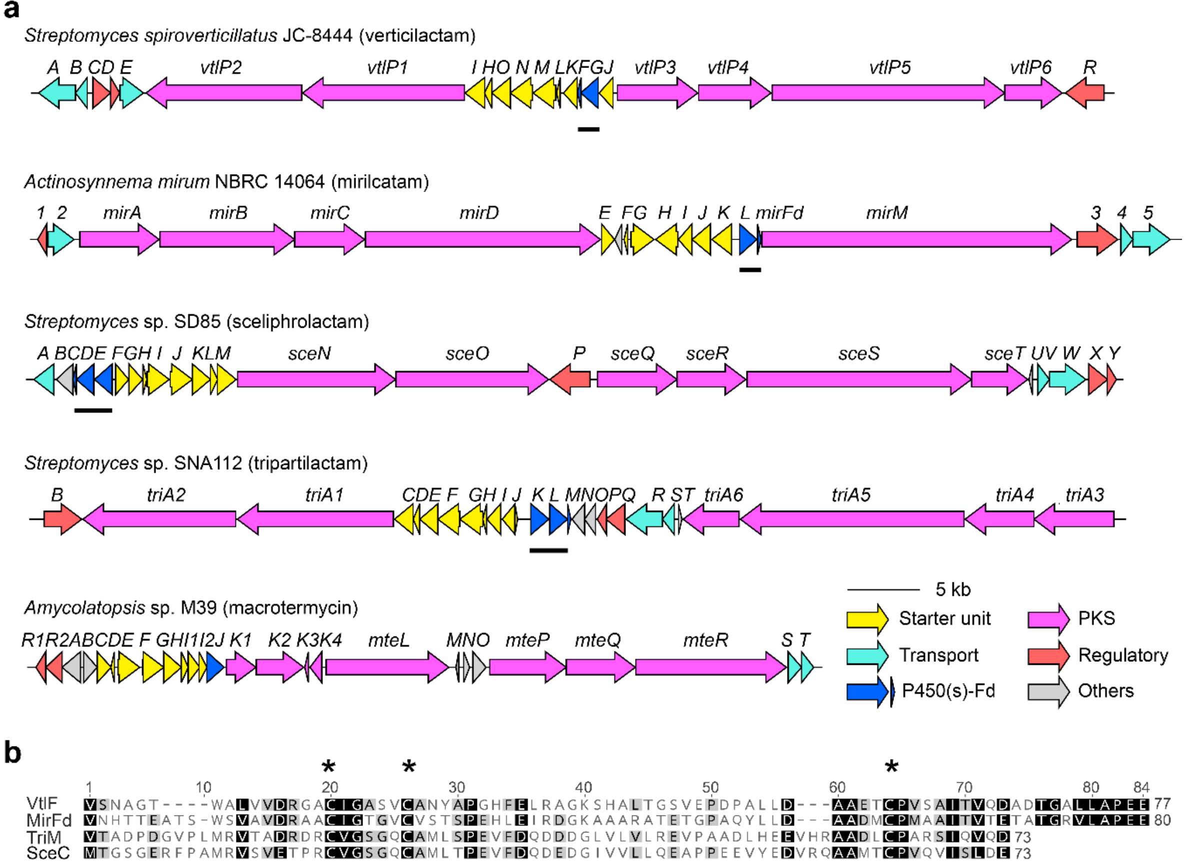

Supplementary Fig. 4 | Comparative analysis of *vtl*, *mir*, *sce*, *tri*, and *mte* gene clusters. **a**, *Mir* gene cluster from *Actinosynnema mirum* NBRC 14064[13], *sce* gene cluster from *Streptomyces* sp. SD85[14], *tri* gene cluster from *Streptomyces* sp. SNA112[9], and *mte* gene cluster from *Amycolatopsis* sp. M39[12]. The presence of the P450-Fd set (*vtl*, *mir*, *sce*, and *tri*) in macrolactam biosynthetic gene clusters was underlined. **b**, Multiple sequence alignment of the actinobacterial Fds. The [3Fe-4S] cluster binding Cys residues in the Cys-X_2-(Y or non-Cys)-X_2-Cys-Xn-Cys-Pro motif are indicated with an asterisk above the sequence.

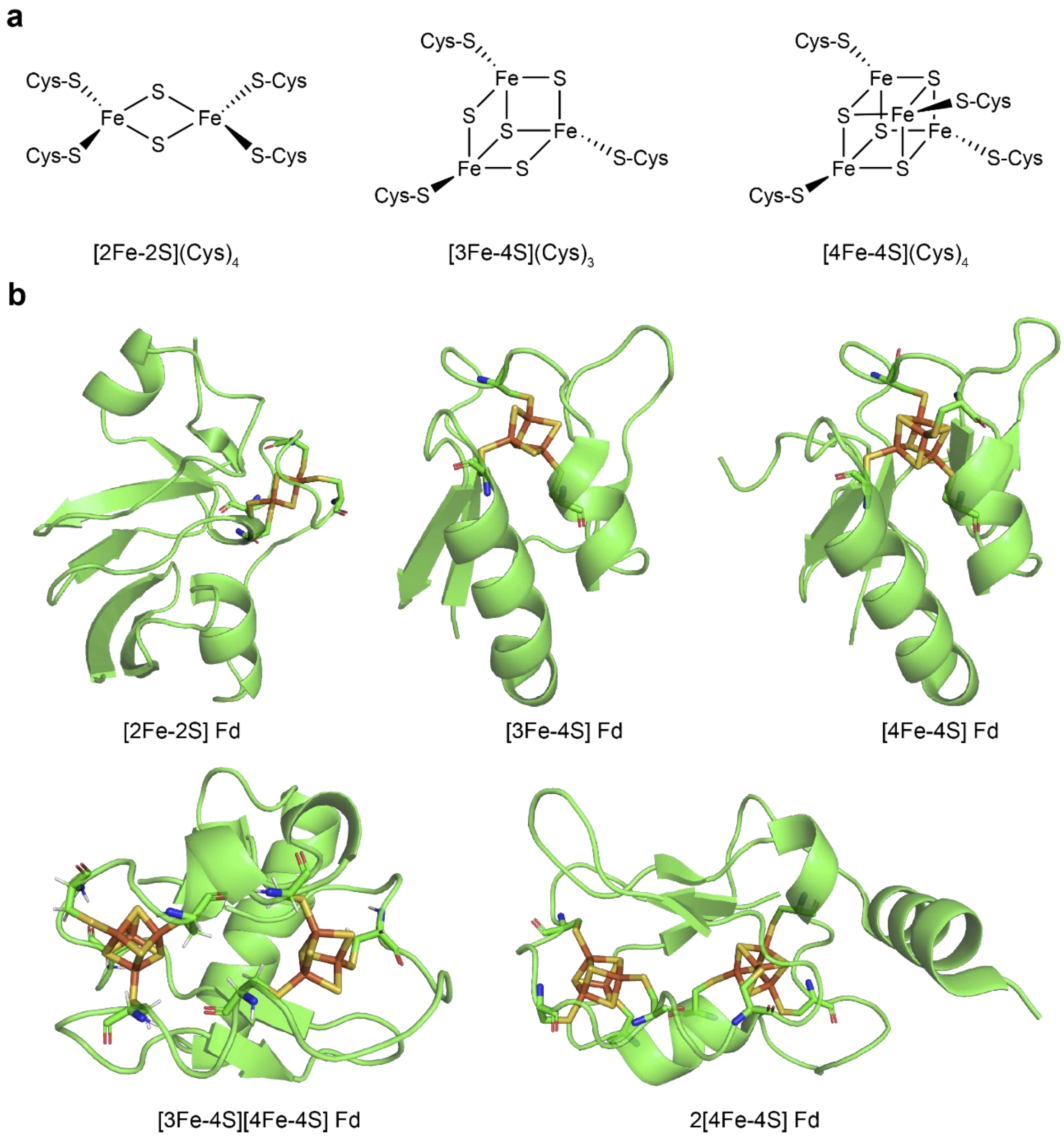

Supplementary Fig. 5 | Natural ferredoxins (Fds) containing Fe-S clusters of various stoichiometries. **a**, Canonical Fe-S clusters in Fds. **b**, Crystal structures of spinach [2Fe-2S] Fd (PDB 1A70), *Mycobacterium tuberculosis* [3Fe-4S] Fd (PDB 8AMP), *Bacillus thermoproteolyticus* [4Fe-4S] Fd form I (PDB 1IQZ), *Bacillus schlegelii* [3Fe-4S][4Fe-4S] Fd (PDB 1BC6), and *E. coli* 2[4Fe-4S] Fd (PDB 2ZVS). The cysteine residue ligands coordinating the Fe-S clusters in each Fd are shown as sticks.

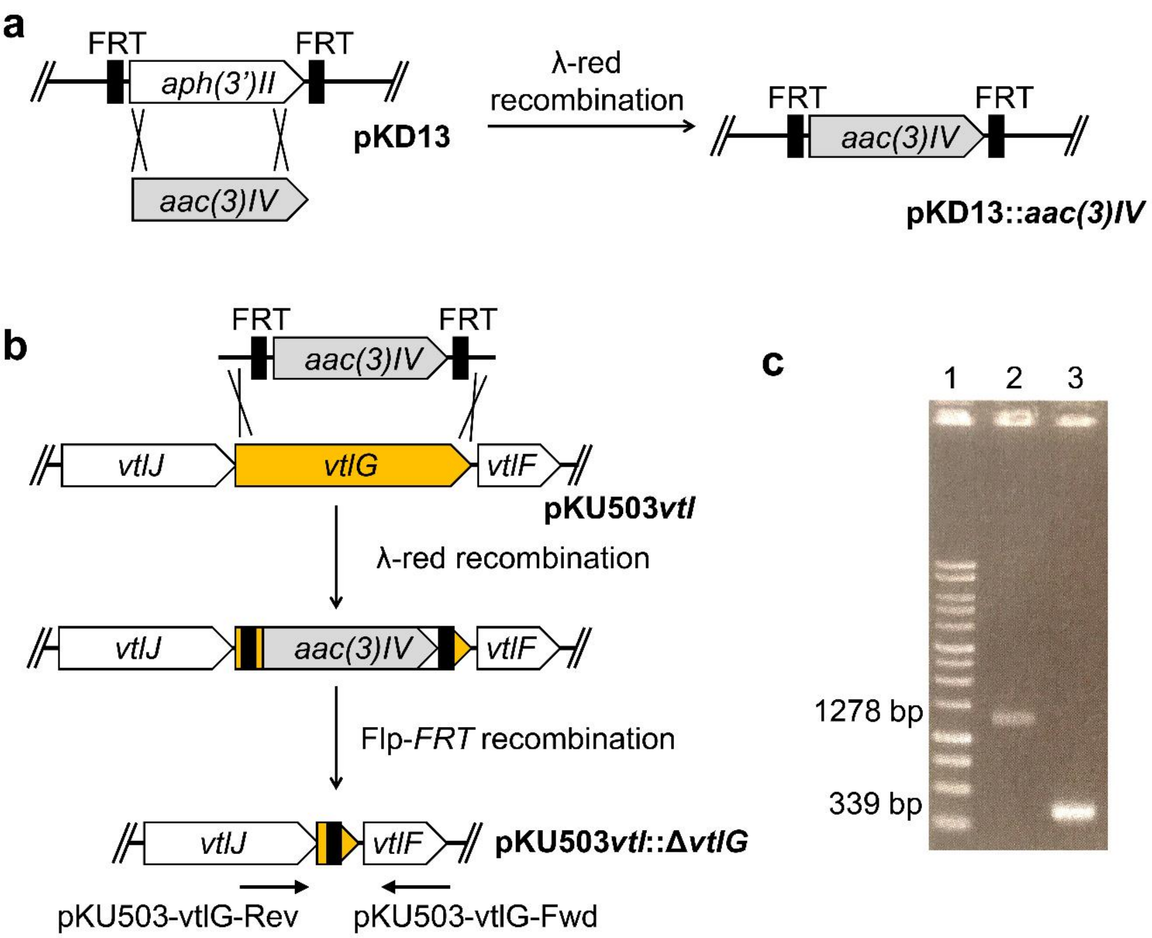

Supplementary Fig. 6 | Disruption of the *vtlG* gene in pKU503*vtl*. **a**, Exchange of antibiotics resistance marker gene in plasmid pKD13 (Supplementary Table 2). **b**, Scheme of in-frame *vtlG* gene deletion in pKU503*vtl*. **c**, PCR products amplified with primer sets of pKU503-vtlG-Fwd and pKU503-vtlG-Rev (Supplementary Table 3) were confirmed by 0.9% agarose gel electrophoresis. Lane 1, 1kb DNA Ladder; lane 2, pKU503*vtl* (1278 bp); lane 3, pKU503*vtl*::Δ*vtlG* (339 bp).

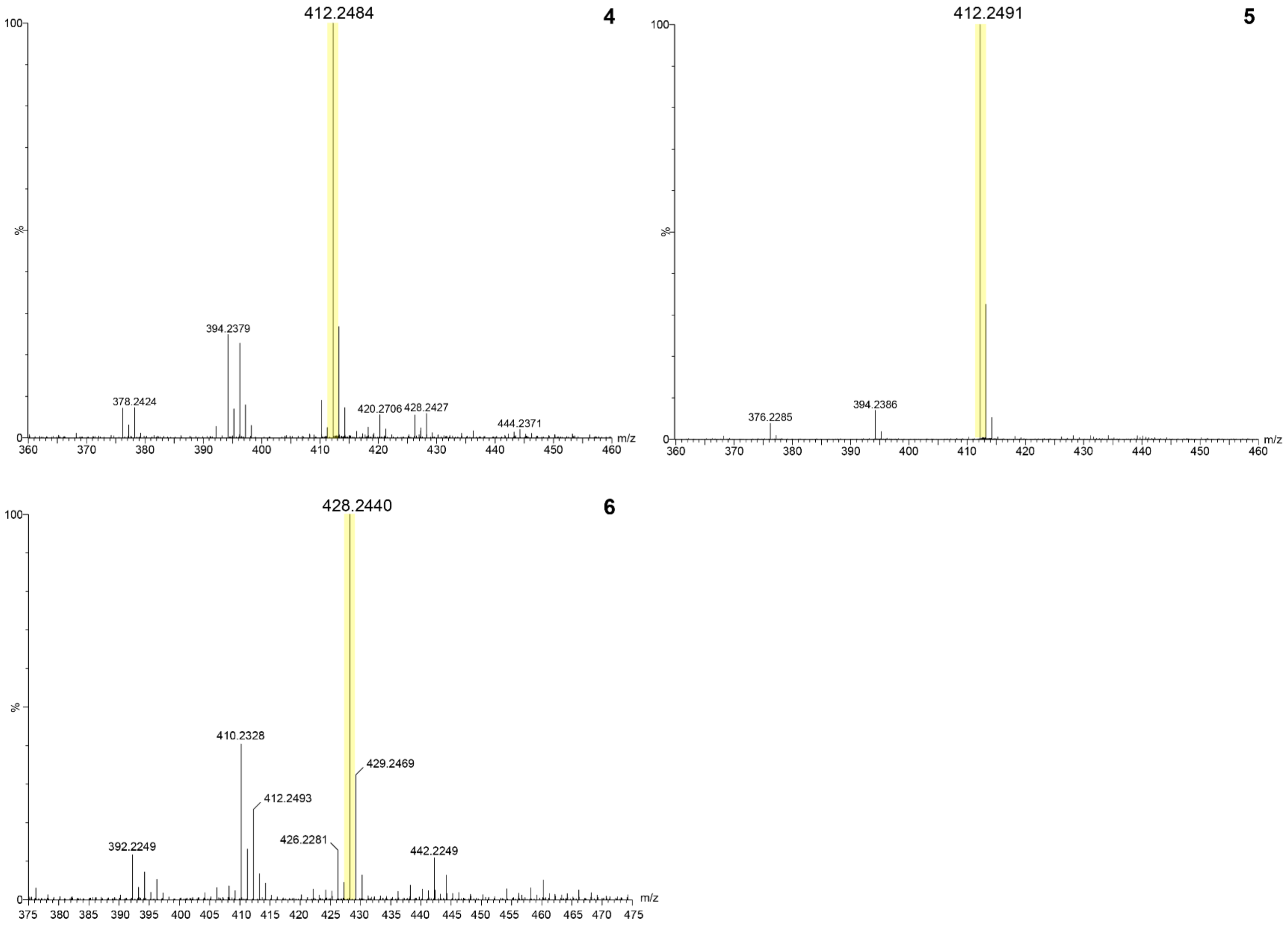

Supplementary Fig. 7 | HR-ESI-TOF-MS spectra of compounds **4**, **5**, and **6**.

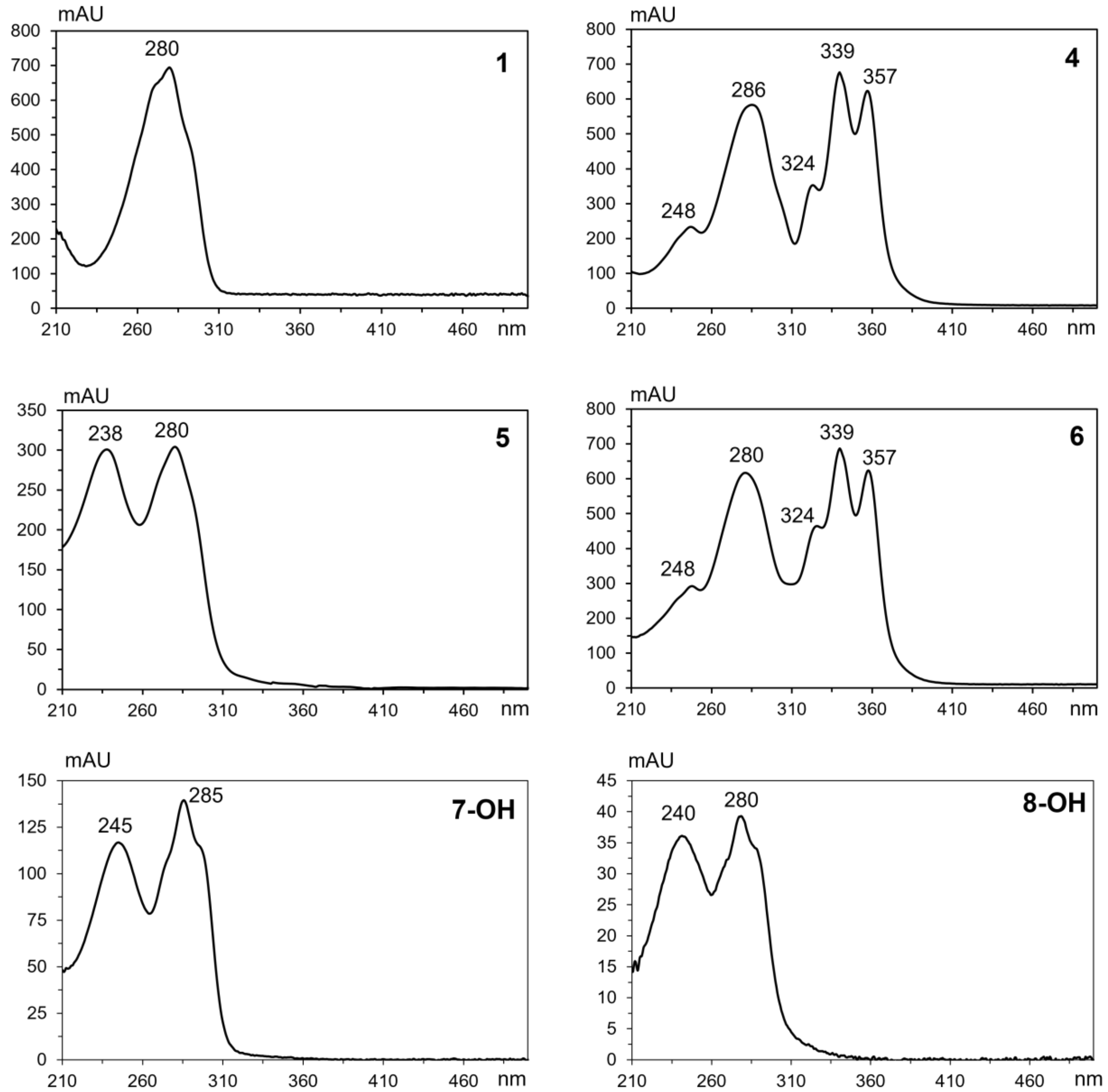

Supplementary Fig. 8 | UV-Vis spectra of compounds **1**, **4**, **5**, **6**, **7-OH**, and **8-OH**.

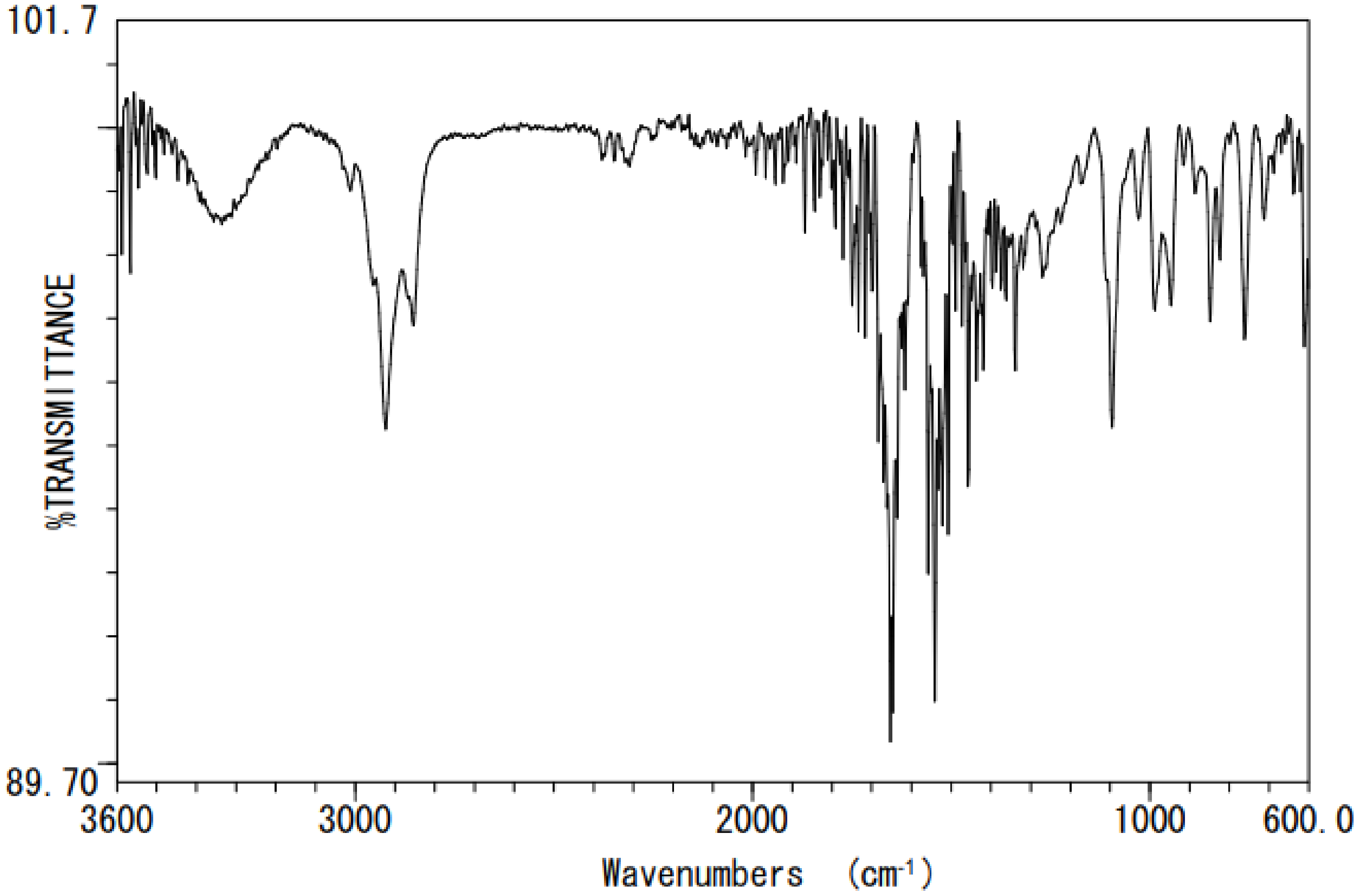

Supplementary Fig. 9 | IR spectrum of compound **5**.

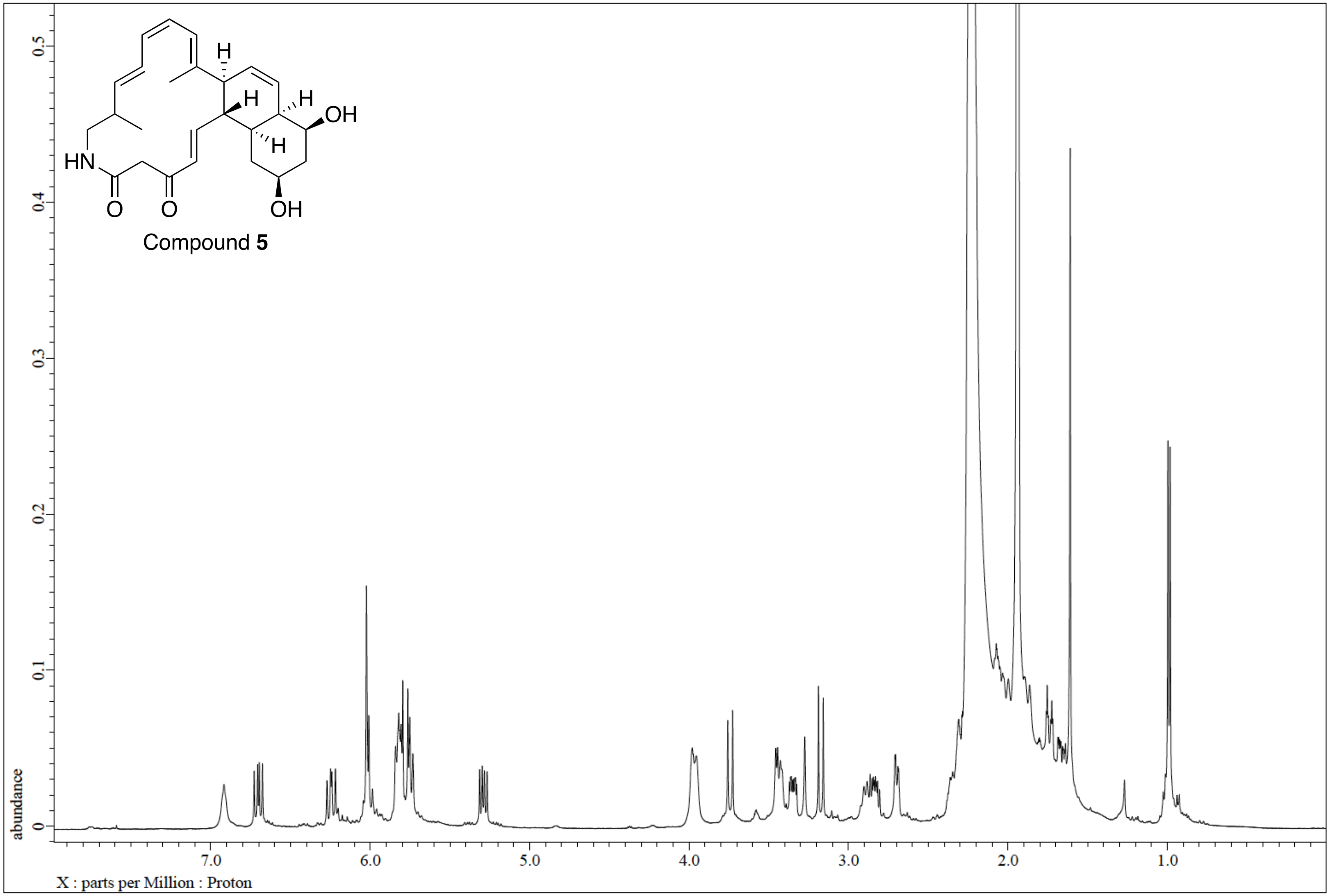

Supplementary Fig. 10 | ^{1}H NMR spectrum of compound **5**.

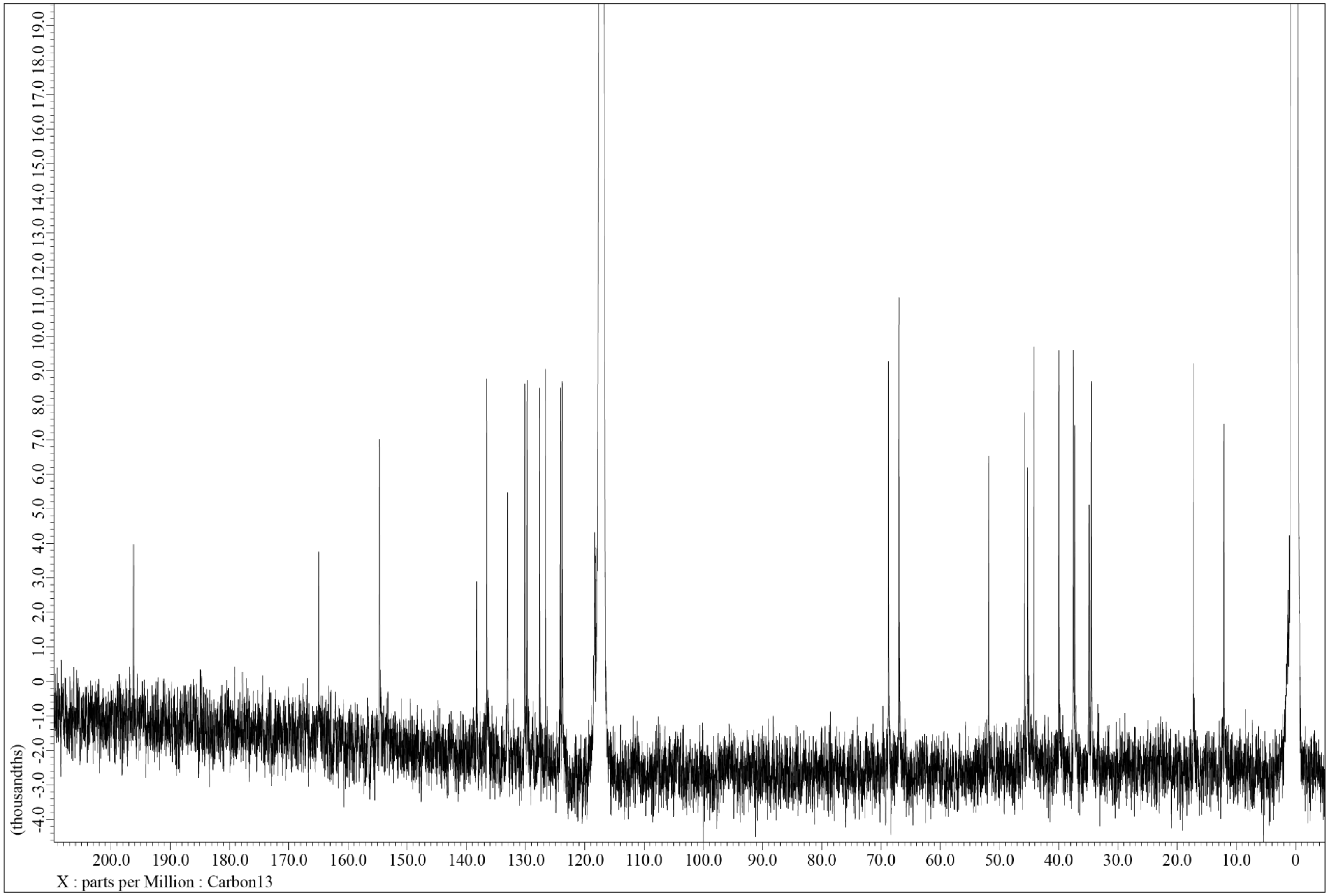

Supplementary Fig. 11 | ^{13}C NMR spectrum of compound **5**.

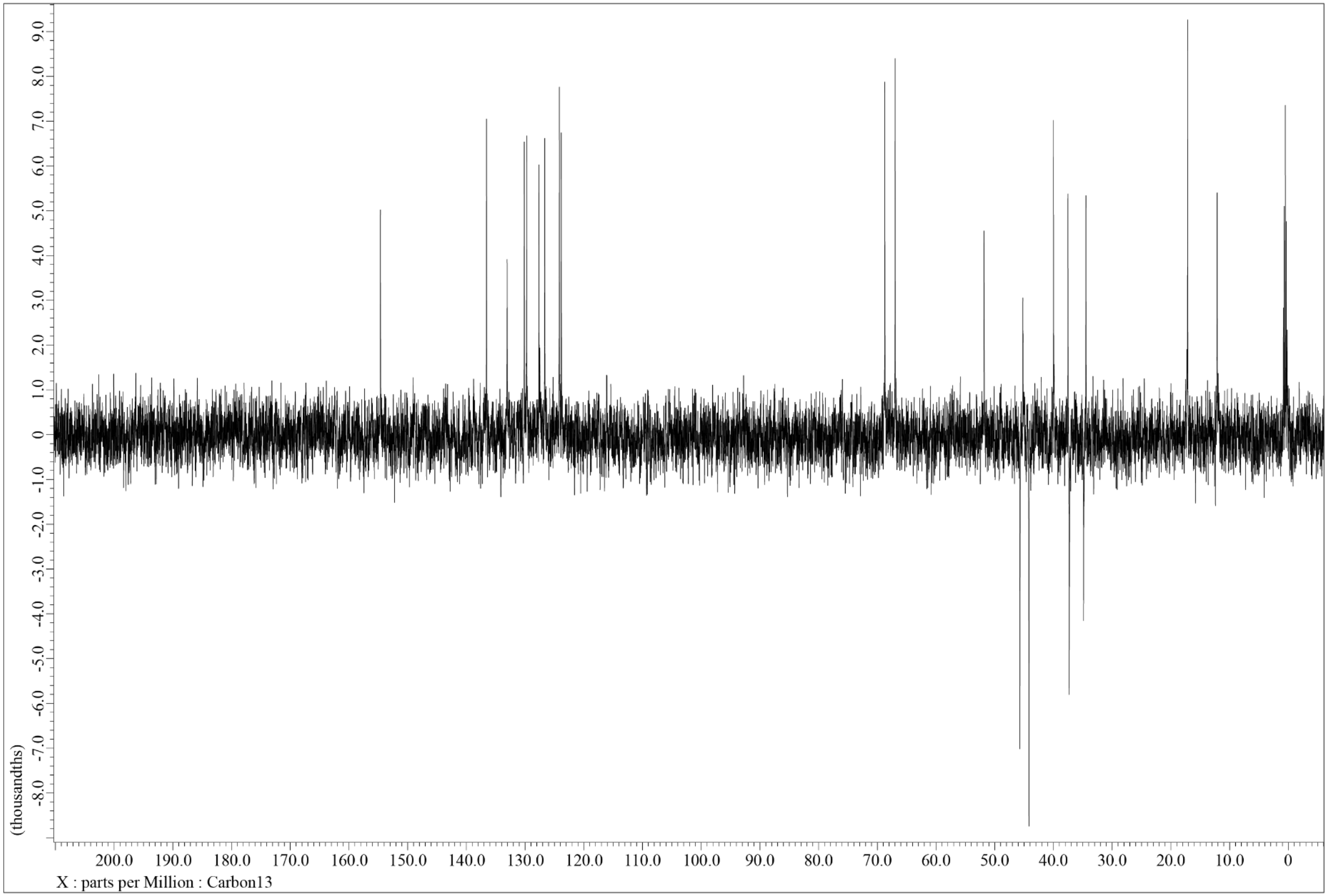

Supplementary Fig. 12 | ^{13}C DEPT spectrum of compound **5**.

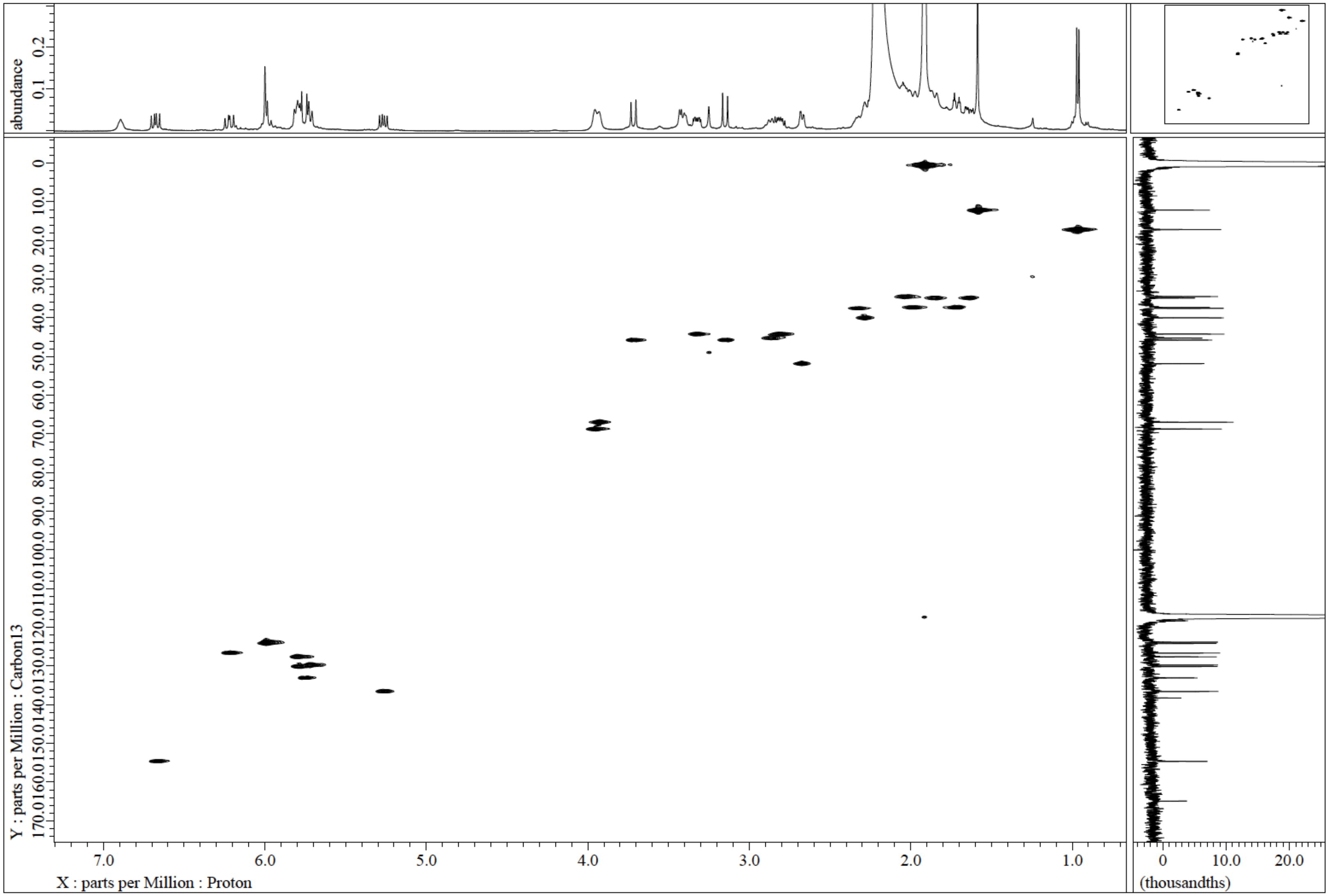

Supplementary Fig. 13 | HSQC spectrum of compound **5**.

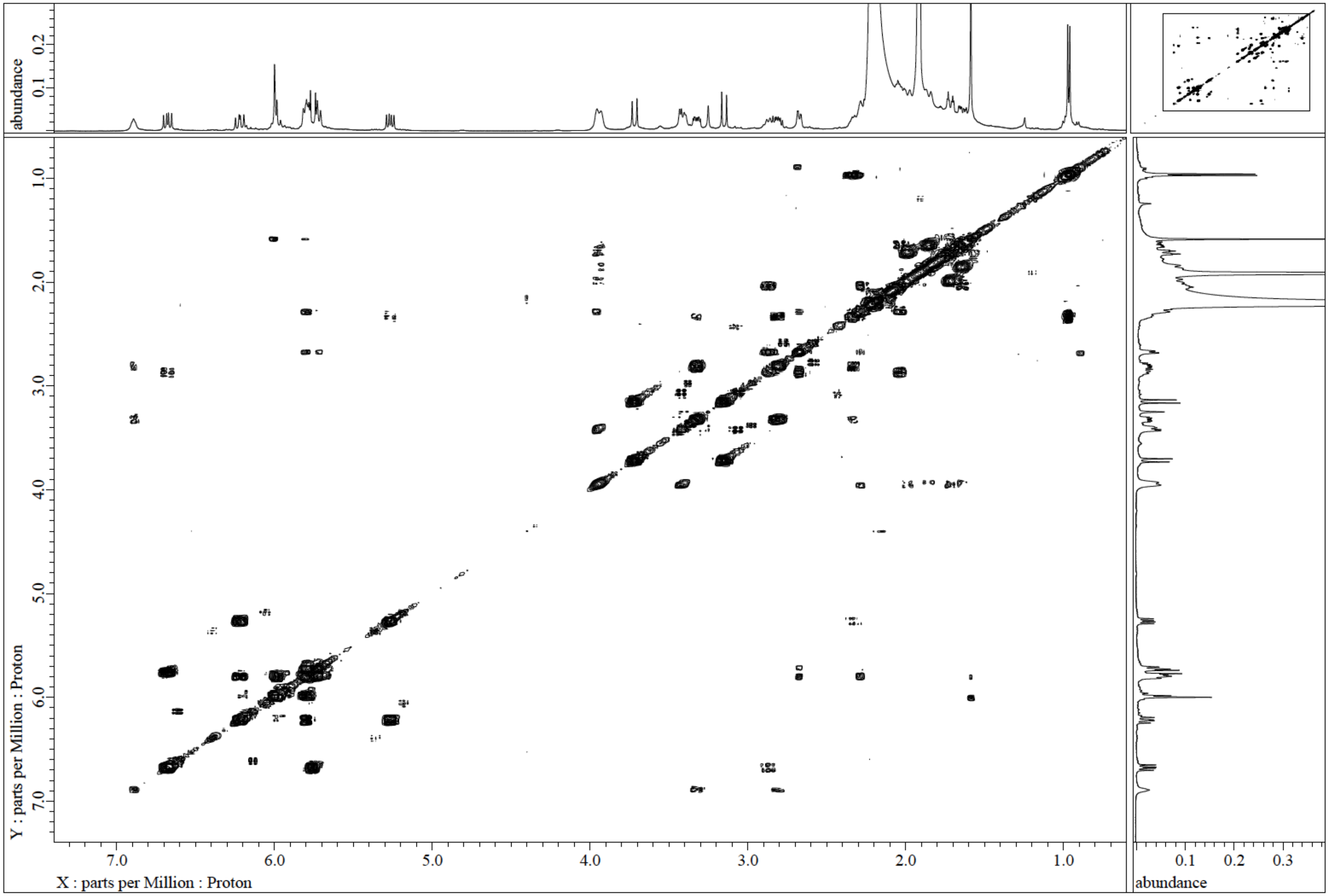

Supplementary Fig. 14 | DQF-COSY spectrum of compound **5**.

273 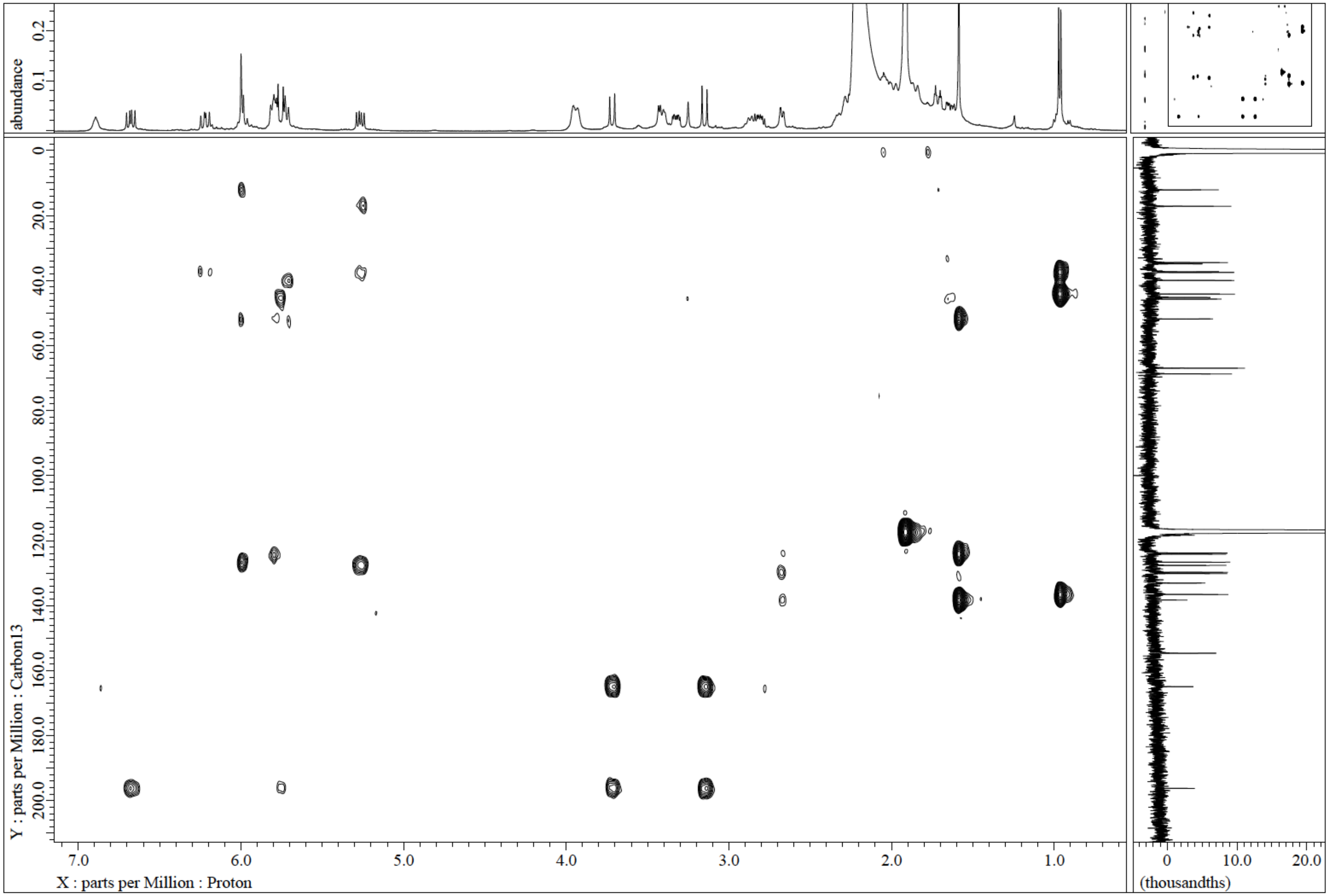

274 **Supplementary Fig. 15 |** HMBC spectrum of compound **5**.

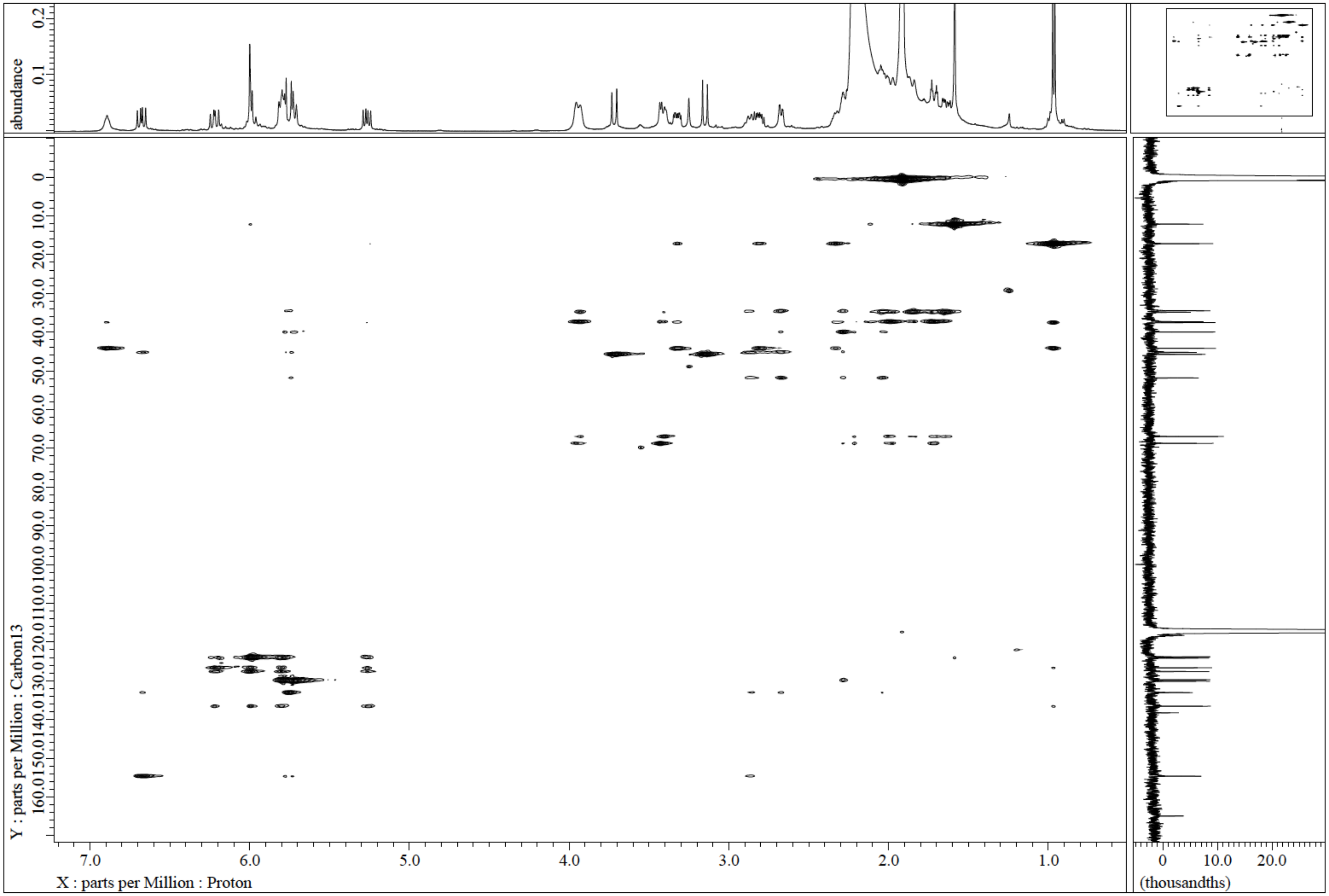

Supplementary Fig. 16 | HSQC-TOCSY spectrum of compound **5**.

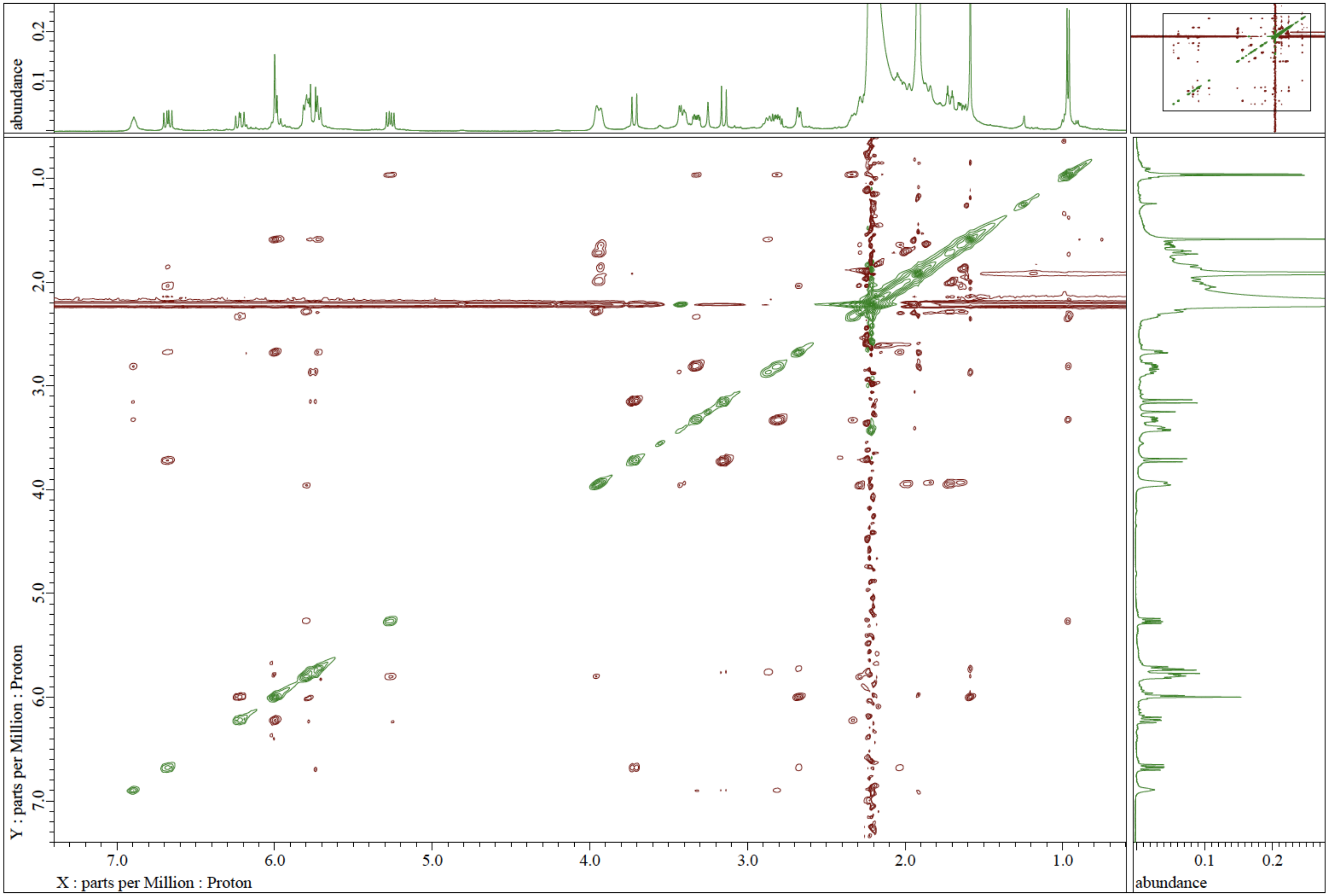

Supplementary Fig. 17 | NOESY spectrum of compound **5**.

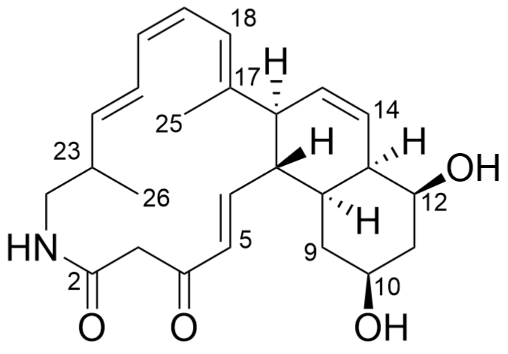

5

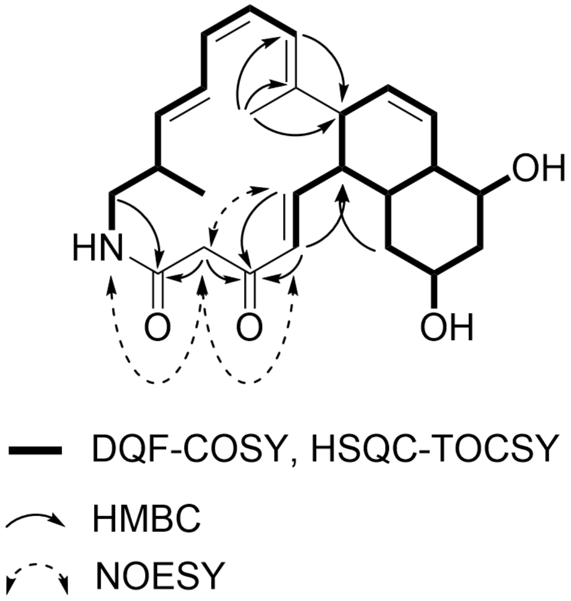

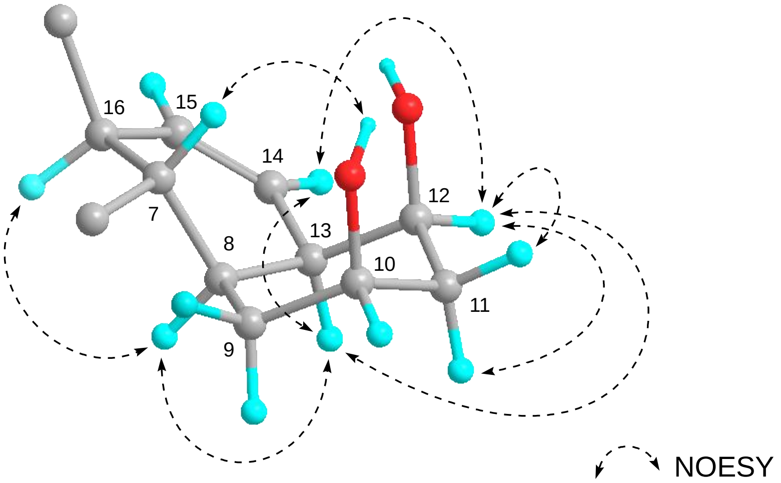

Supplementary Fig. 18 | Key 2D NMR and NOESY correlations of verticilactam S (**5**).

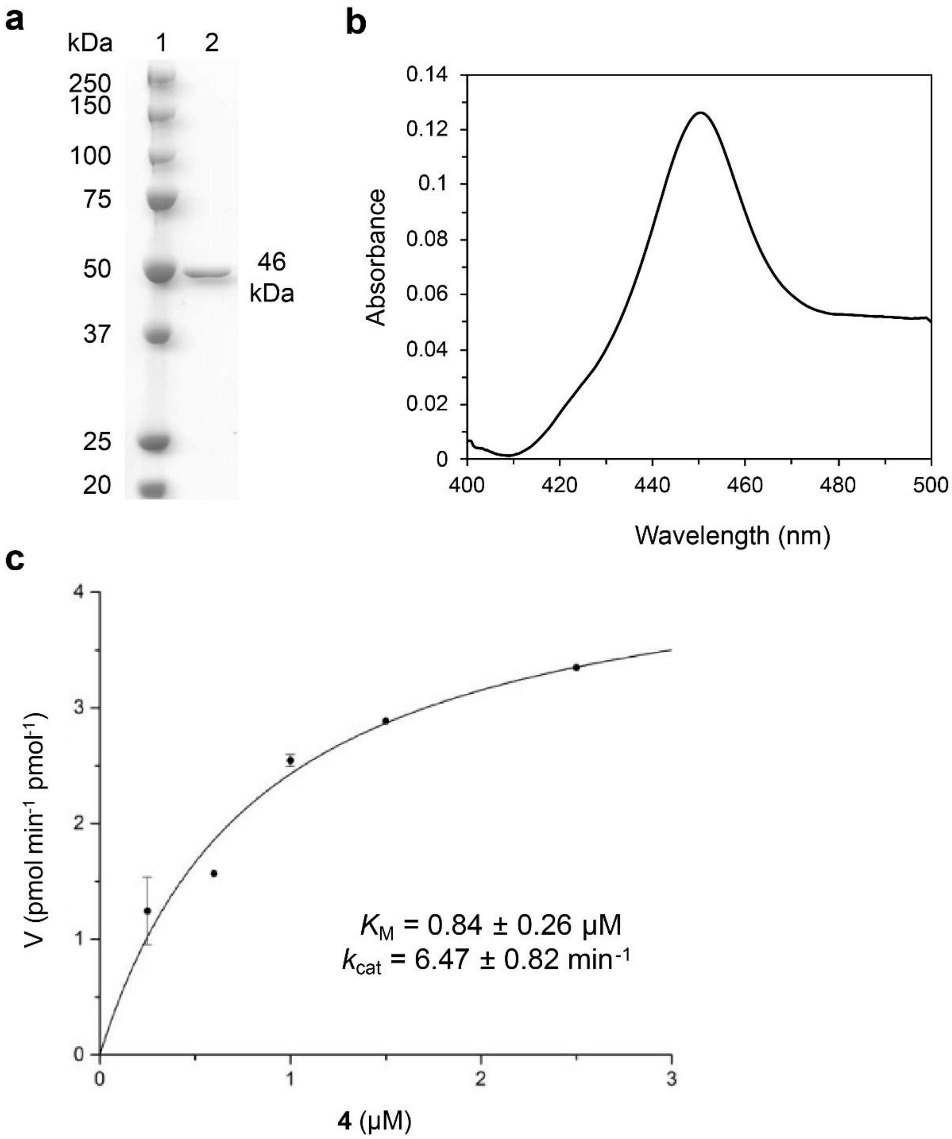

Supplementary Fig. 19 | Characterization of VtlG as a cytochrome P450. **a**, 12.5% SDS-PAGE analysis of recombinant VtlG purified from *E. coli*. Lane 1, protein molecular mass marker; lane 2, His_8-tagged VtlG. **b**, CO difference spectrum. **c**, Kinetic analysis of VtlG-catalysed hydroxylation against **4**. Error bars represent standard deviation (s.d.) obtained from three independent replicates (n=3). The catalytic efficiency (k_{cat}/K_M) was calculated to be 7.7 (min^{-1} μM^{-1}). Source data are provided as a Source Data file.

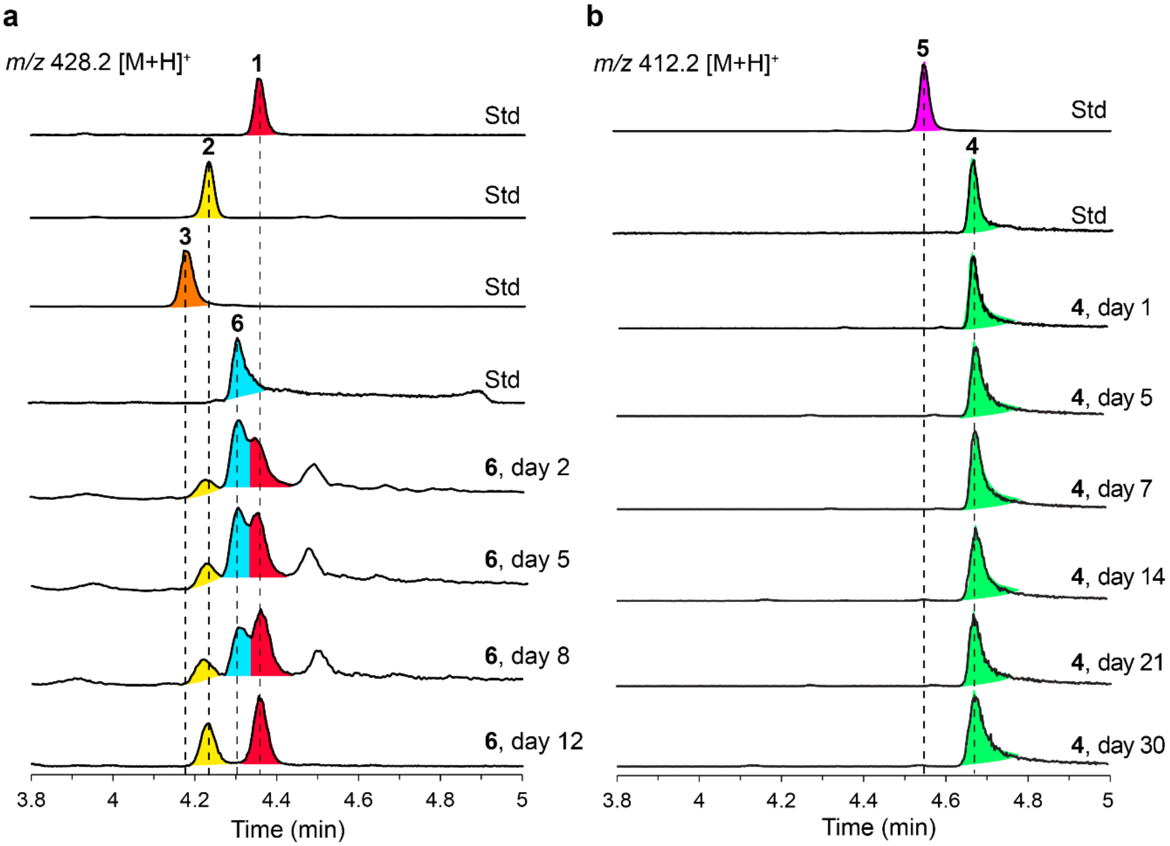

Supplementary Fig. 20 | Nonenzymatic conversion of **6** and **4**. **a**, 1 µM **6** was incubated in 50 mM Tris-HCl (pH 7.5) at 30°C for 12 days. **b**, 10 µM **4** was incubated in 50 mM Tris-HCl (pH 7.5) at 30°C for 30 days. UPLC-MS monitored the nonenzymatic reaction.

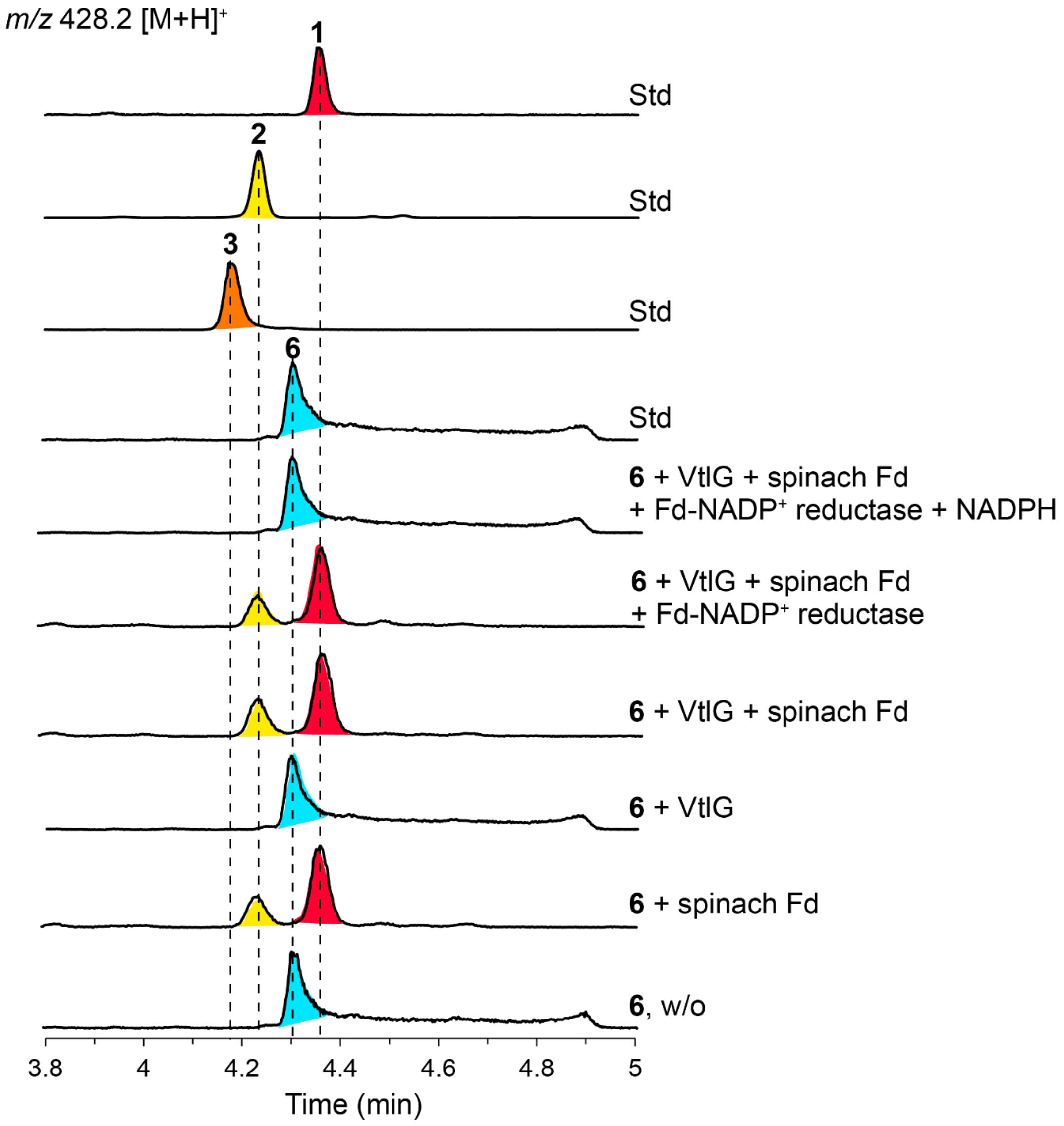

293

294 **Supplementary Fig. 21 |** *In vitro* conversion of purified **6** (1 µM) in the presence or absence
295 of VtlG, spinach Fd, spinach FNR, and NADPH. The reactions were performed in 50 mM
296 Tris-HCl (pH 7.5) at 30°C for 30 min. The reaction product was monitored by UPLC-MS.
297 w/o, without enzyme.

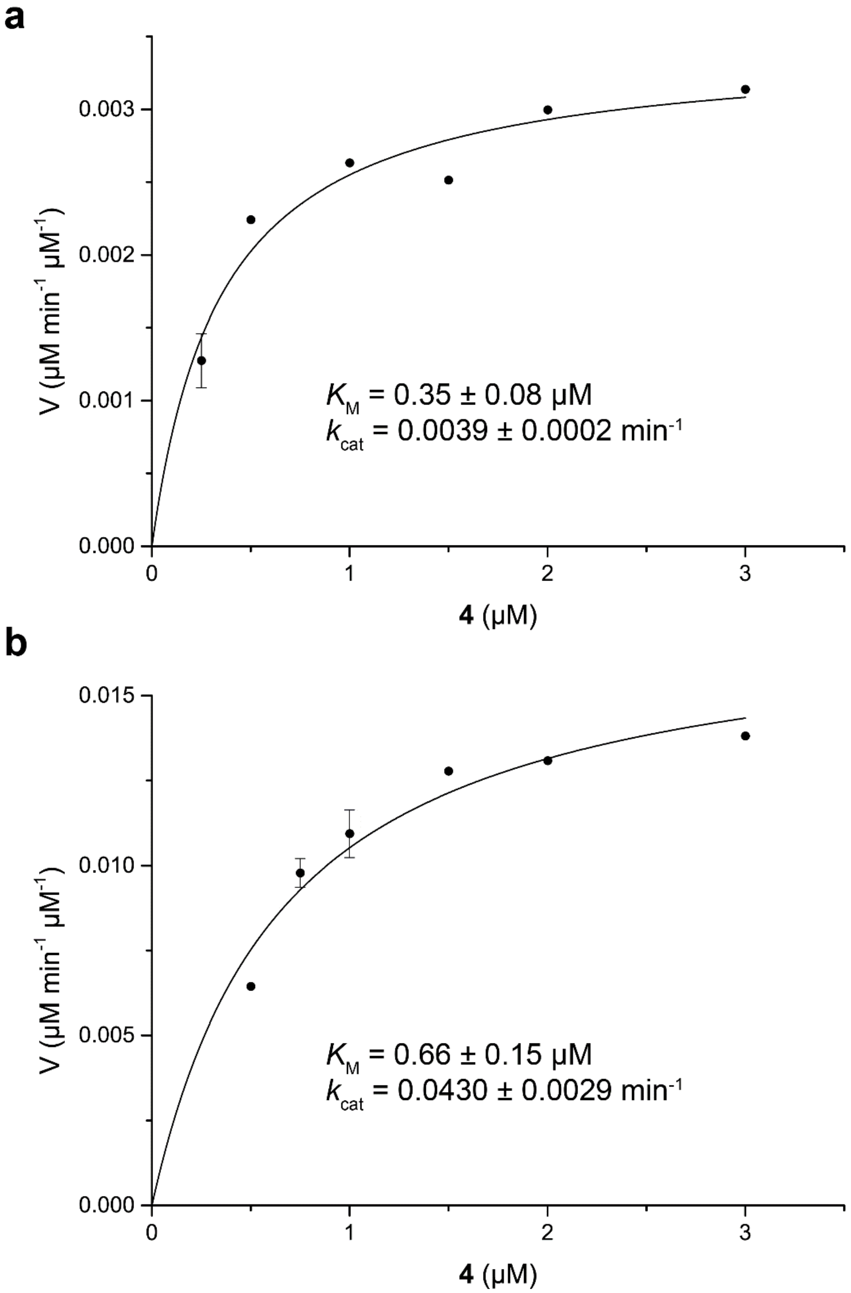

Supplementary Fig. 22 | Kinetic analysis of spinach Fd **(a)** and MirFd **(b)** catalysed [4+2] cycloadditions against **4**. Error bars represent standard deviation (s.d.) obtained from three independent replicates (n=3). The catalytic efficiencies (k_{cat}/K_M) for spinach Fd and MirFd were calculated to be 0.011 and 0.065 (min^{-1} μM^{-1}), respectively.

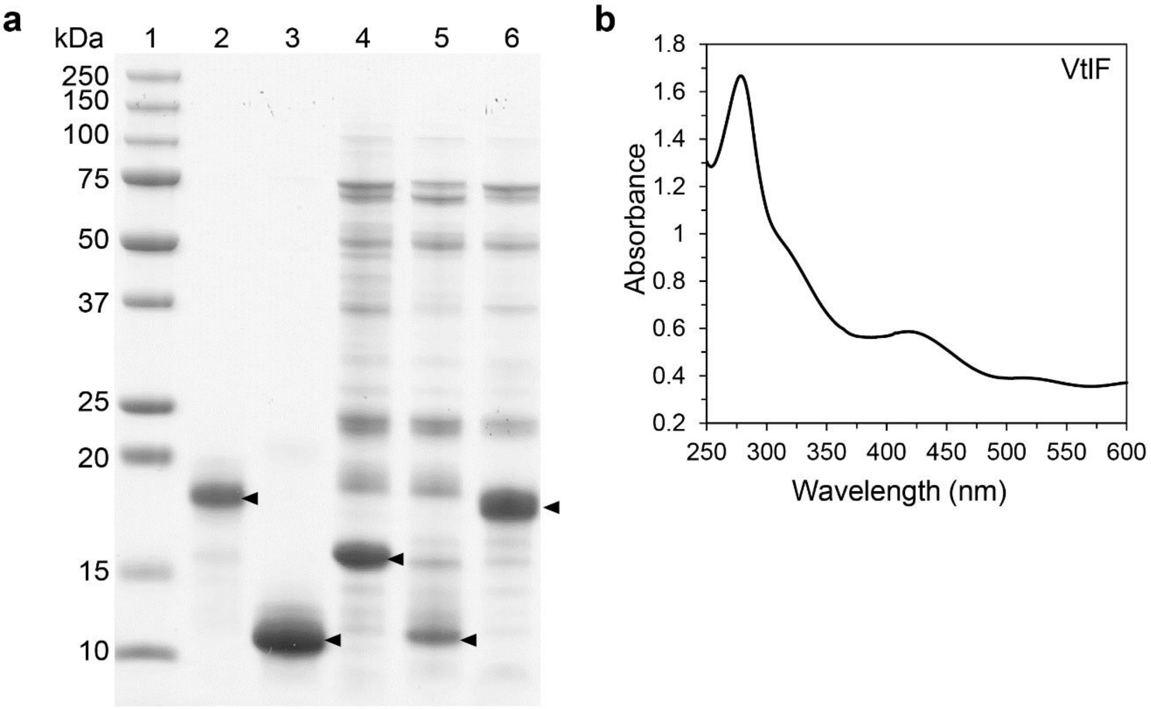

Supplementary Fig. 23 | Characterization of recombinant Fds. **a**, 15% SDS-PAGE analysis of His_6-tagged Fds purified from *E. coli*. Lane 1, protein molecular mass marker; lane 2, recombinant spinach Fd; lane 3, MirFd; lane 4, VtlF; lane 5, SceC; lane 6, TriM. **b**, Absorption spectrum of purified VtlF. Source data are provided as a Source Data file.

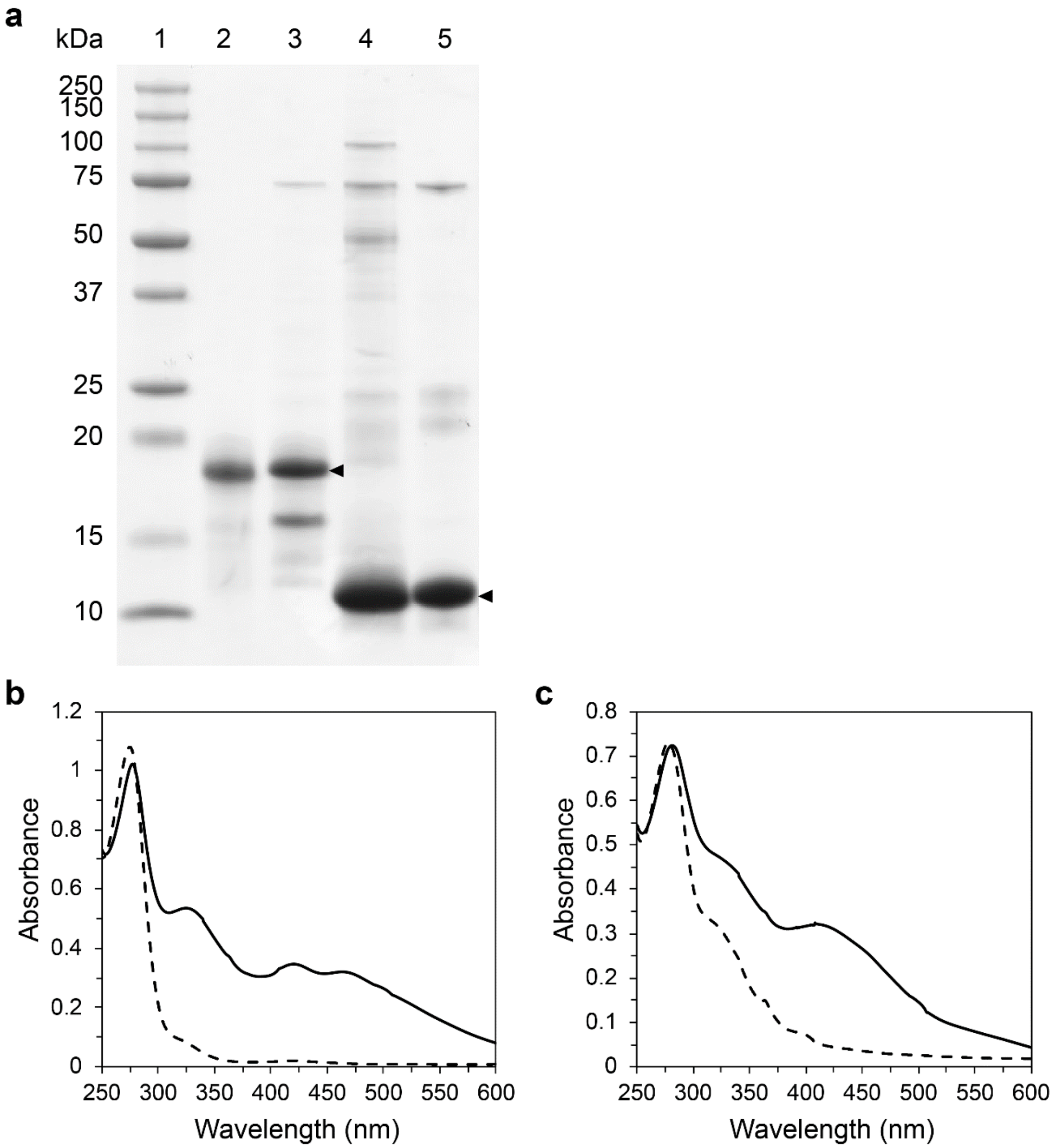

Supplementary Fig. 24 | Protein purification of apo-spinach Fd and apo-MirFd mutants from *E. coli*. **a**, 15% SDS-PAGE analysis. Lane 1, protein molecular mass marker; lane 2, wild-type spinach Fd; lane 3, mutant spinach Fd C40A; lane 4, wild-type MirFd; lane 5, mutant MirFd C19A. **b**, Absorption spectra of wild-type spinach Fd (holo-form, solid line) and mutant spinach Fd C40A (apo-form, dotted line). **c**, Absorption spectra of wild-type MirFd (holo-form, solid line) and mutant MirFd C19A (apo-form, dotted line). Source data are provided as a Source Data file.

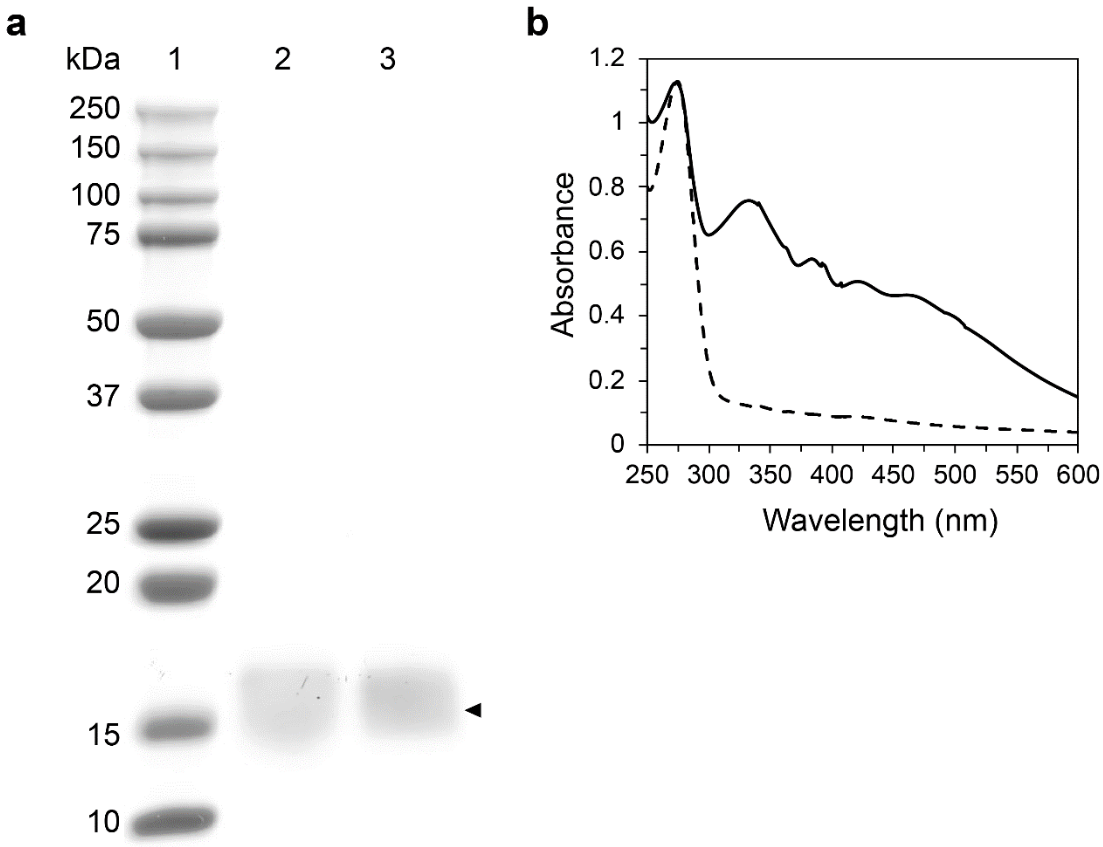

Supplementary Fig. 25 | Preparation of *Synechocystis* Fd. **a**, 15% SDS-PAGE analysis. Lane 1, protein molecular mass marker; lane 2, *Synechocystis* sp. PCC6803 wild-type Fd (SynFd) purified from *E. coli*; lane 3, gallium-substituted SynFd (GaFd). **b**, Absorption spectra of SynFd (solid line) and GaFd (dotted line). Source data are provided as a Source Data file.

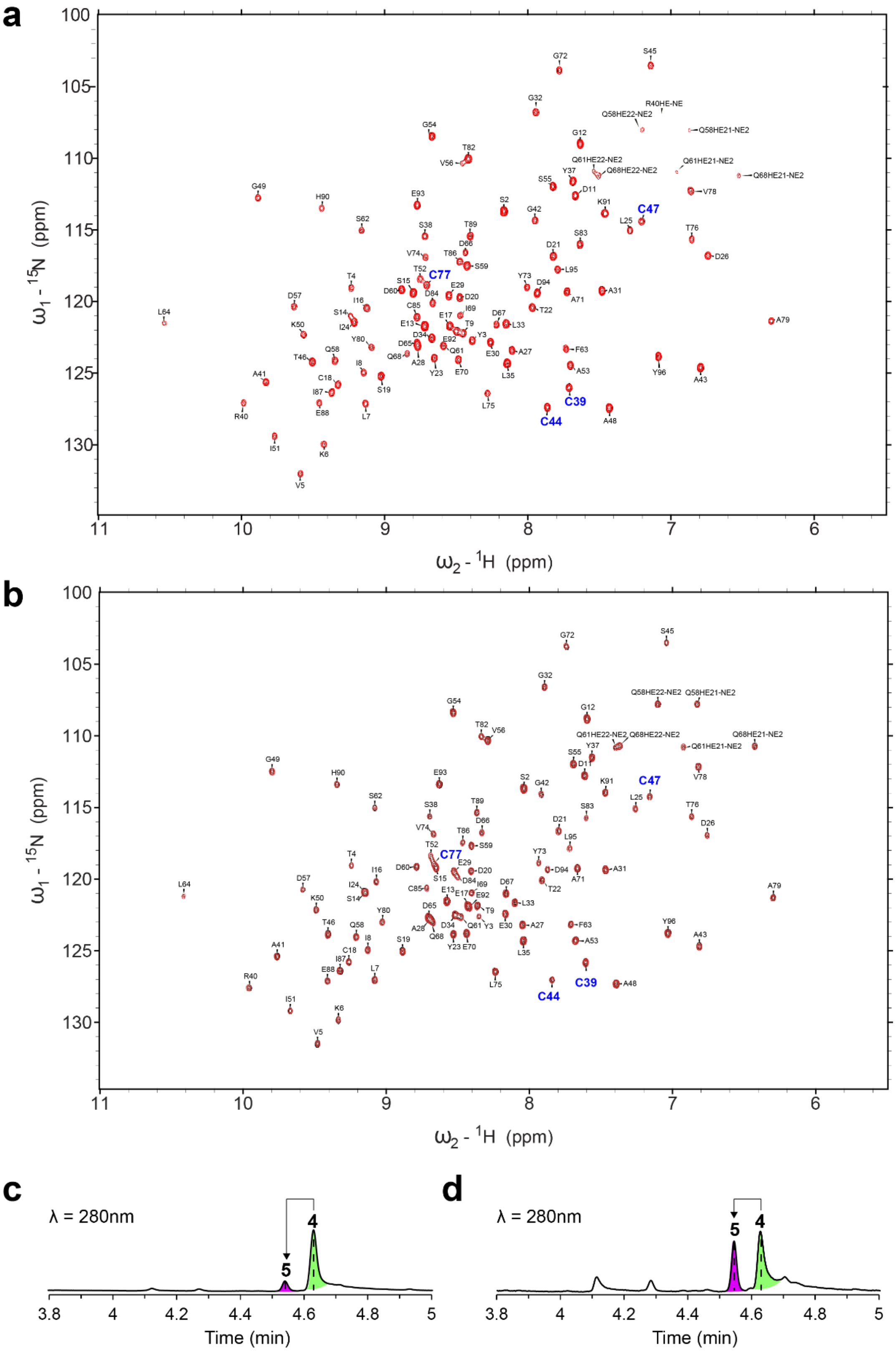
a
ω1 - 15N (ppm)
ω2 - 1H (ppm)
C39
C44
C47
C77
b
ω1 - 15N (ppm)
ω2 - 1H (ppm)
C39
C44
C47
C77
c
λ = 280nm
5
4
Time (min)
d
λ = 280nm
5
4
Time (min)

Supplementary Fig. 26 | NMR analysis of [^{15}N]-labelled GaFd. The superposition of ^{1}H-^{15}N HSQC spectra of 10 μM [^{15}N]-labelled GaFd alone (black) and with 10 μM substrate **4** (red) obtained at 277 K (**a**) and 298 K (**b**), respectively. Positions of the [2Ga-2S] cluster coordinating Cys residues are coloured by blue. No detectable chemical shifts were observed upon the titration of substrate **4** with variable moral ratios. UPLC-MS monitoring of the [^{15}N]-labelled GaFd catalysed [4+2] cycloaddition of **4** after NMR analysis at 277 K (**c**) and 298 K (**d**), respectively.

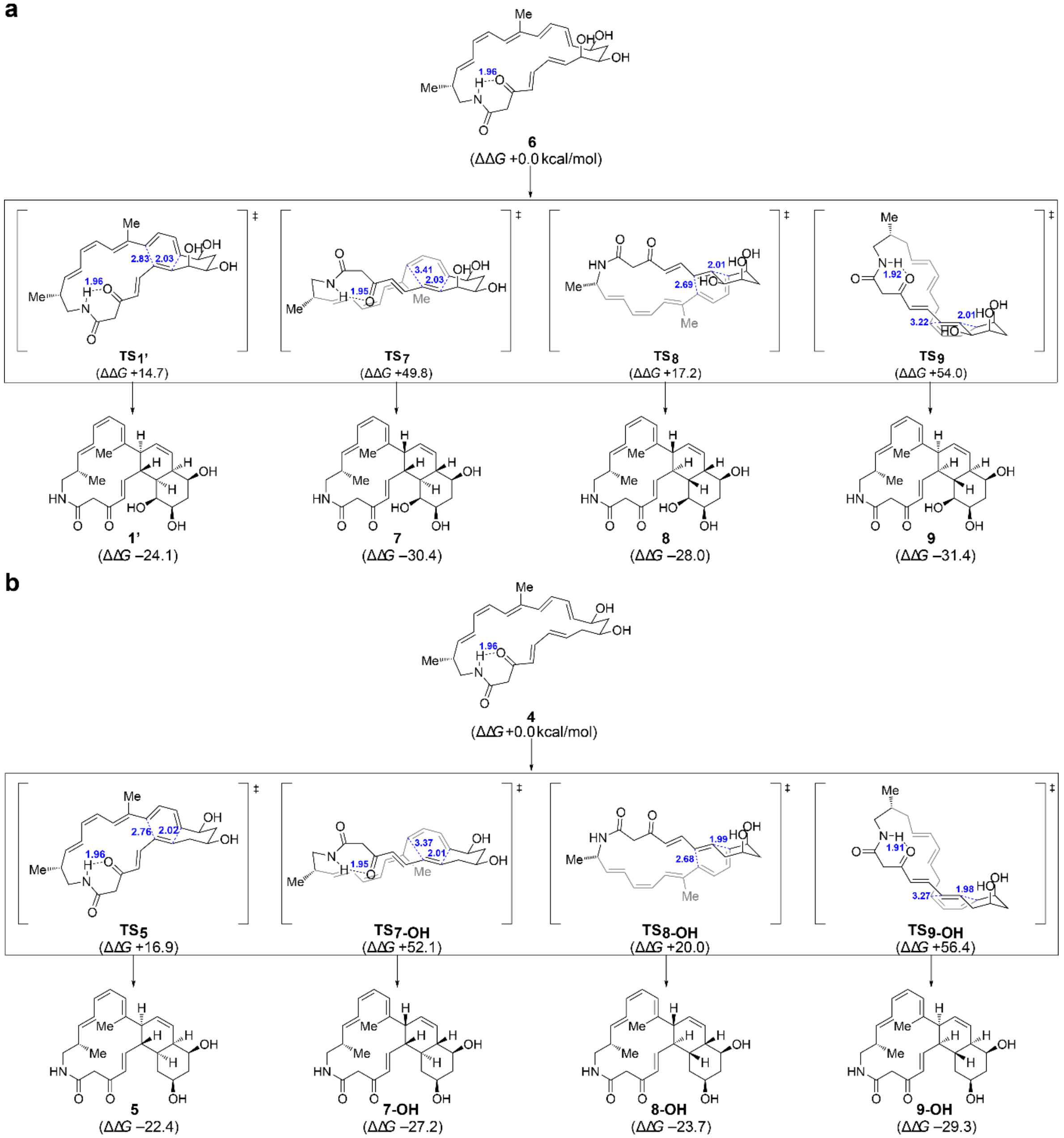

Supplementary Fig. 27 | The DFT calculations for the nonenzymatic [4+2] cycloadditions. Four transition-state structures and associated activation barriers for forming a decalin skeleton from **6** (**a**) and **4** (**b**). Activation-free energies ($\Delta G^{\ddagger}$) calculated at the M06-2X/6–311+G**(scrf=CPCM, water) level of theory are given in kcal mol^{-1} and distances in Å.

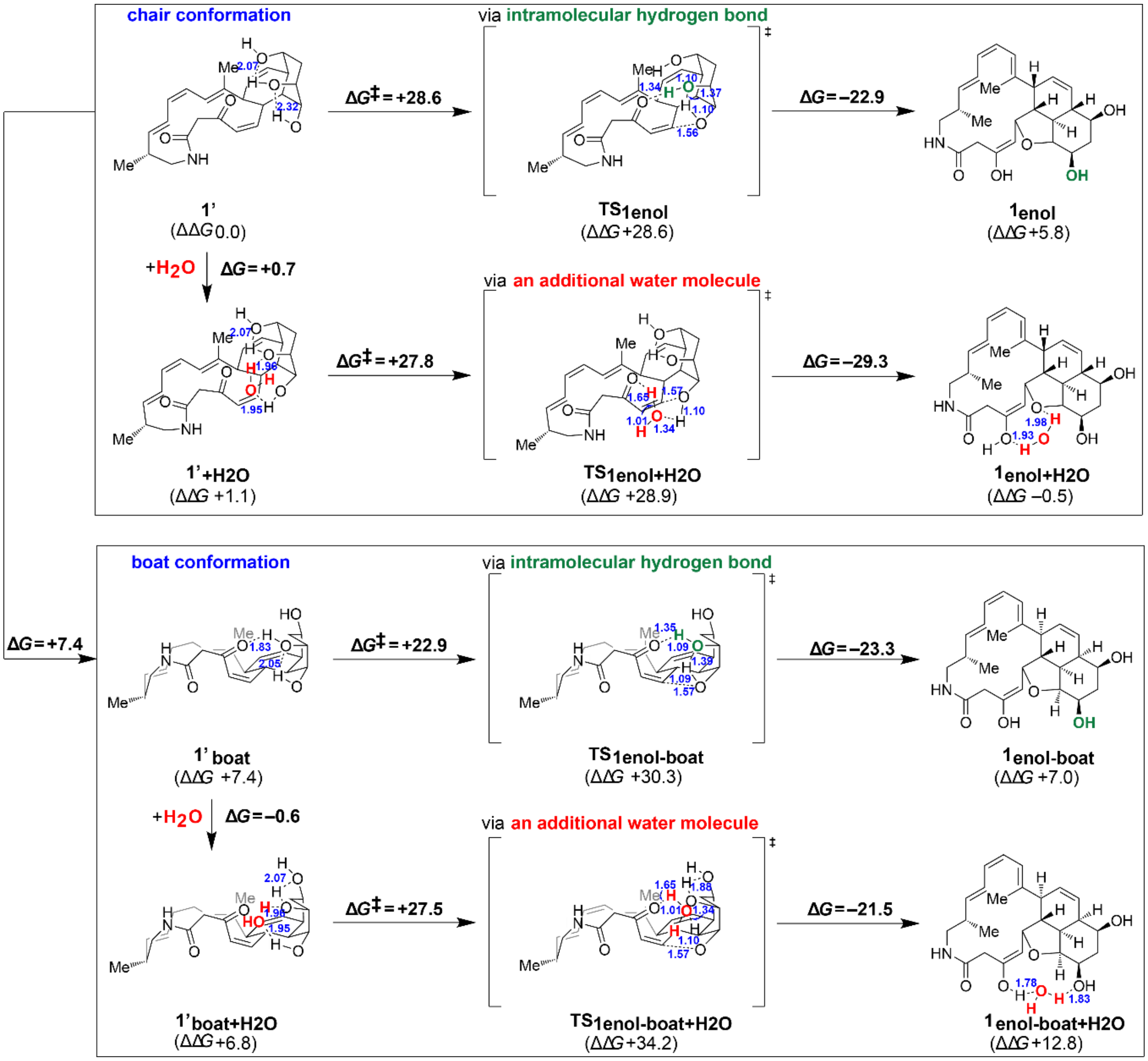

Supplementary Fig. 28 | The DFT calculation for the nonenzymatic Michael additions of **1'**. Four reaction pathways for the Michael addition of **1'** with a chair or boat conformation via an intramolecular hydrogen bond or an additional water molecule. Activation-free energies ($\Delta G^{\ddagger}$) calculated at the M06-2X/6–311+G**(scrf=CPCM, water) level of theory are in kcal mol^{-1} and distances in Å.

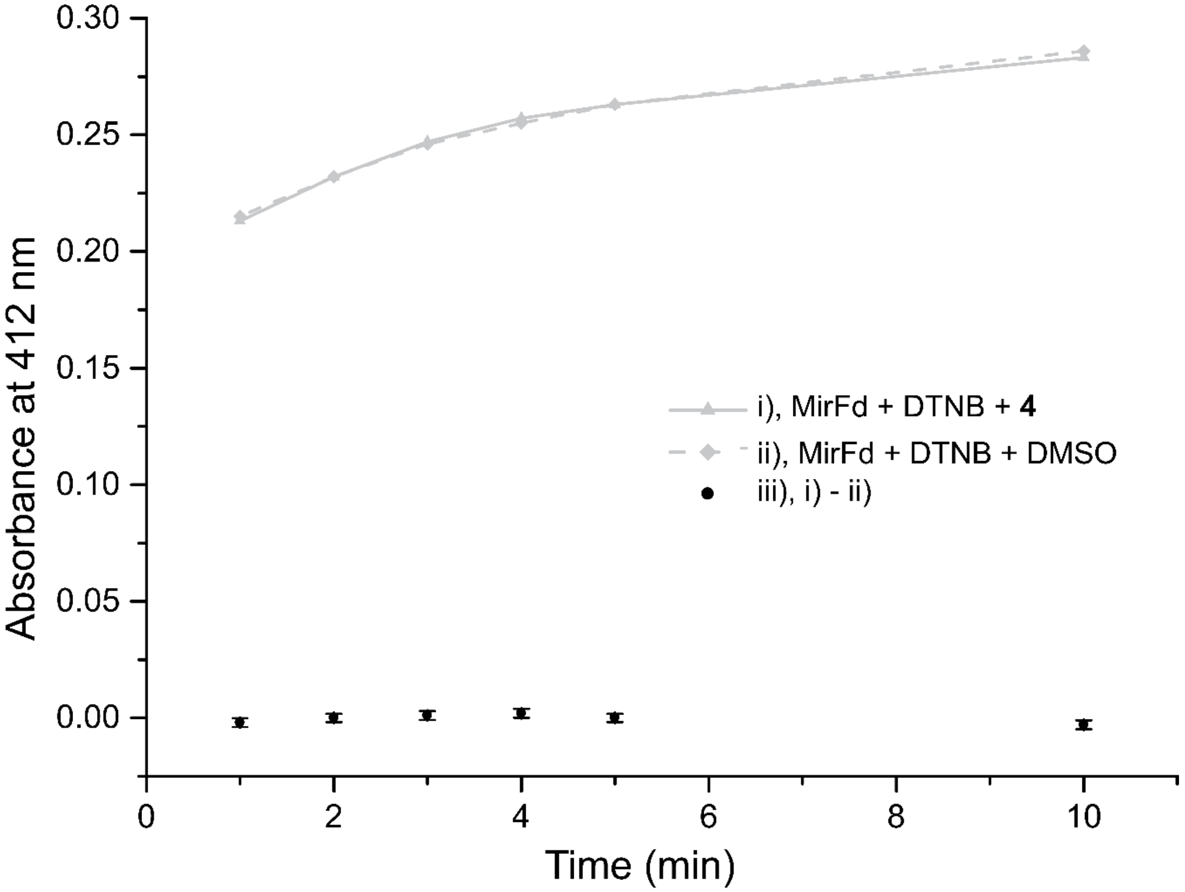

Supplementary Fig. 29 | Time-dependent *in vitro* assay of 0.1 mM DTNB, 20 μM [3Fe-4S](Cys)$_3$ MirFd, and i) 24 μM substrate **4** (grey solid line) or ii) DMSO as control (grey dashed line) at room temperature (24 °C). Absorbances were monitored at 412 nm. iii), the cleavage of Cys residues coordinating the [3Fe-4S] cluster was not detected by calculating with i) – ii) (black dots). Error bars represent standard deviation (s.d.) obtained from five independent replicates (n=3).

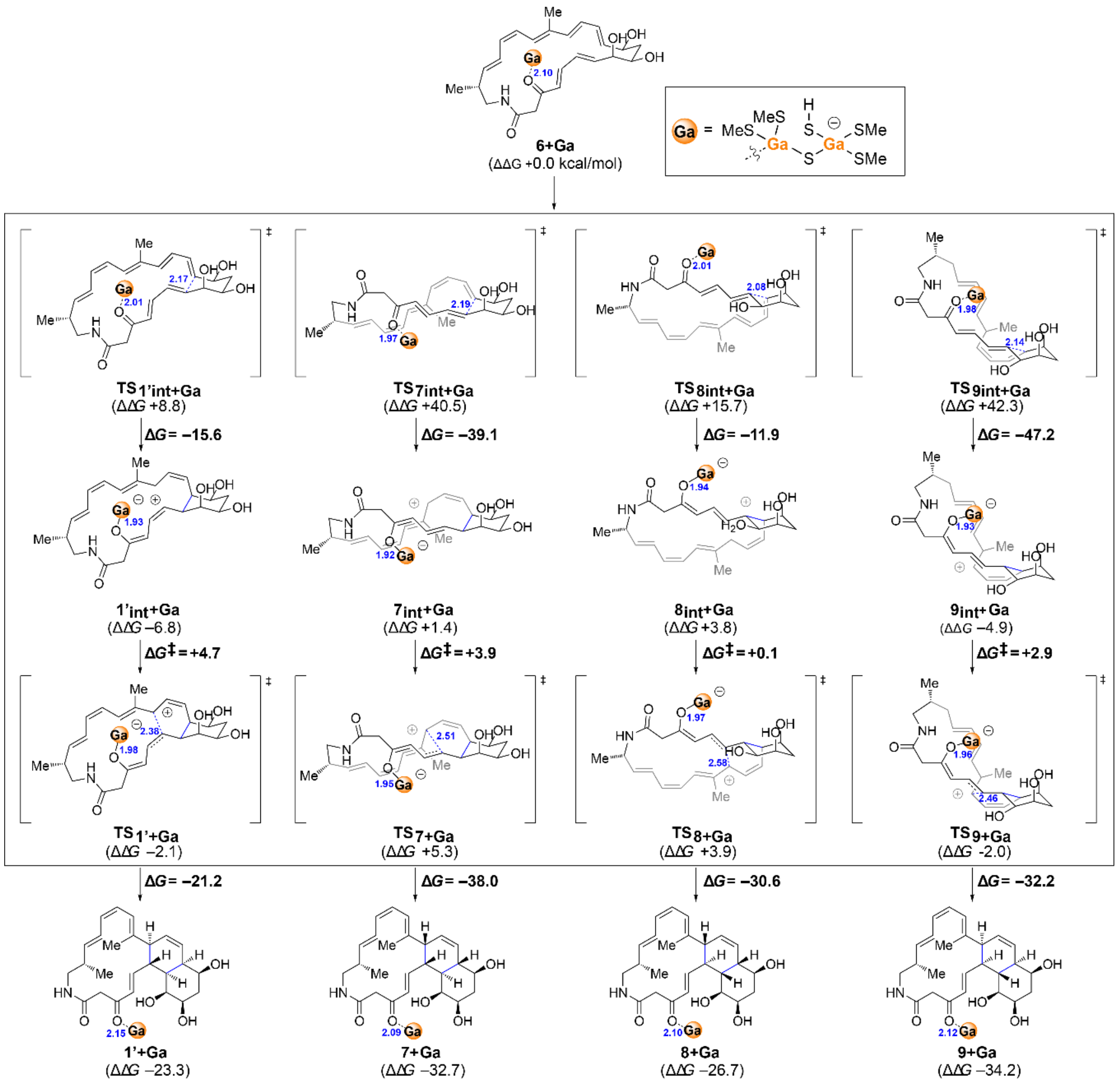

Supplementary Fig. 30 | The DFT calculations for the [4+2] cycloadditions of **6** with a [2Ga-2S] cluster. Four reaction pathways and associated activation barriers for forming a decline skeleton from **6+Ga**. Activation-free energies ($\Delta G^{\ddagger}$) calculated at the M06-2X/SDD and 6–311+G** (scrf=CPCM, water) level of theory are given in kcal mol^{-1} and distances in Å.

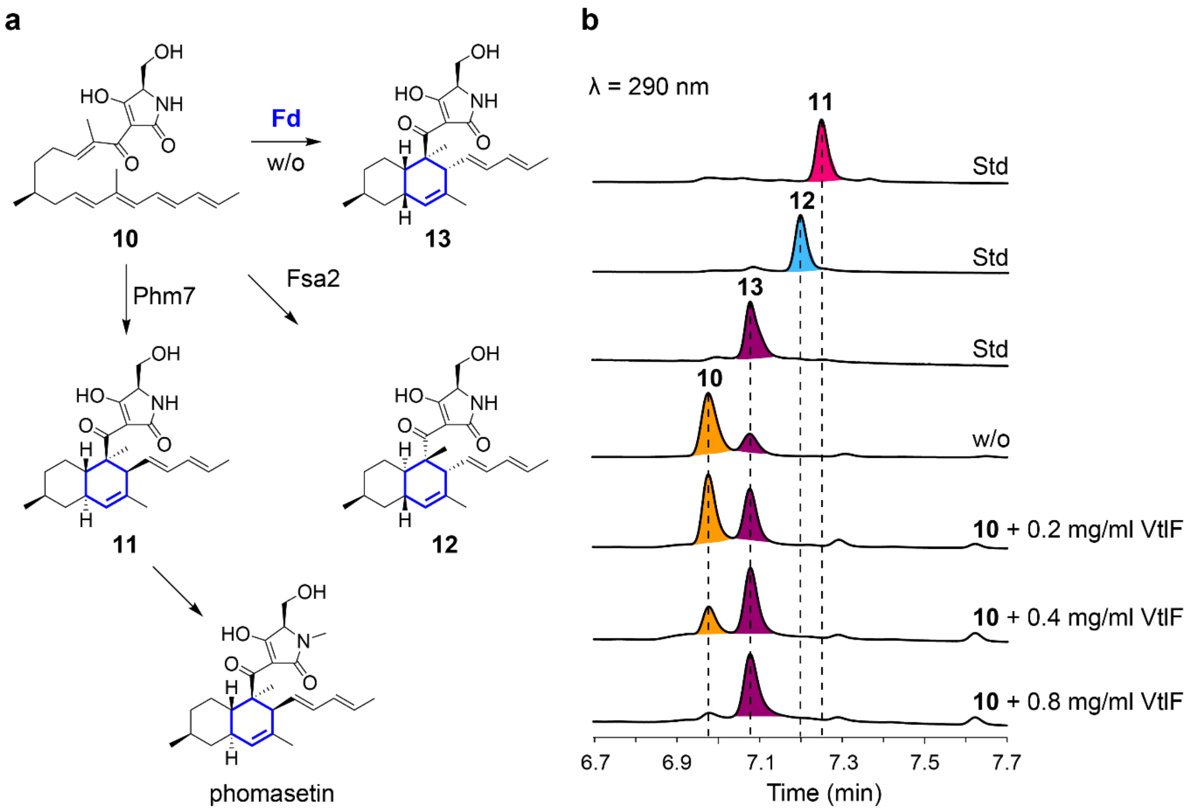

Supplementary Fig. 31 | Application of Fds as versatile biocatalysts for [4+2] cycloaddition. **a**, Stereospecific [4+2] cycloadditions of the linear polyenoyl tetramic acid **10**. **b**, The *in vitro* reaction of **10** with VtlF were conducted on ice in 50 mM Tris-HCl (pH 7.5) for 5 min. w/o, without enzyme. The reaction products were monitored by UPLC-MS.

Supplementary Tables

Supplementary Table 1 | ^{1}H and ^{13}C NMR chemical shifts of verticilactam S (**5**) in acetonitrile-d_3.

Position	δ_C	δ_H (*multi*, *J* in Hz)	Position	δ_C	δ_H (*multi*, *J* in Hz)
2	165.9	–	15	130.8	5.74 (br d, 10.3)
3	46.8	3.17 (d, 15.5)	16	52.8	2.70 (m)
		3.74 (d, 15.5)	17	139.3	–
4	197.2	–	18	124.9*	6.03 (d, 10.9)
5	134.1	5.77 (d, 16.1)	19	125.2*	6.01 (dd, 10.9, 10.9)
6	155.7	6.70 (dd, 16.1, 10.4)	20	128.6	5.82 (br dd, 10.9, 10.9)
7	46.2	2.89 (br d, 10.4)	21	127.7	6.24 (dd, 14.9, 10.9)
8	35.5	2.06 (m)	22	137.6	5.29 (dd, 14.9, 8.6)
9	35.9	1.66 (ddd, 14.3, 6.9, 2.9)	23	38.6	2.35 (m)
		1.88 (br d, 14.3)	24	45.2	2.83 (m)
10	68.0	3.94 (br s)			3.35 (ddd, 13.2, 5.7, 3.4)
11	38.4	1.74 (ddd, 14.3, 2.9, 2.9)	25	13.2	1.61 (3H, s)
		2.02 (br d, 14.3)	26	18.2	0.99 (3H, d, 6.8)
12	69.8	3.98 (br s)	1–NH	–	6.92 (br s)
13	41.0	2.30 (m)	10–OH	–	3.42 (br d, 4.0)
14	131.2	5.81 (m)	12–OH	–	3.45 (br d, 5.8)

*Interchangeable.

Supplementary Table 2 | Bacterial strains and vectors/plasmids.

**Plasmids/vectors**	**Descriptions**	**Sources**
pKU503	BAC vector containing neomycin resistant gene, *aph(3')II*, and ampicillin resistance gene, for heterologous gene expression	[15]
pKU503*vtl*	BAC vector pKU503 containing the entire verticilactam biosynthetic gene cluster for heterologous expression	[16]
pKU503*vtl*::Δ*vtlG*	pKU503*vtl*, which contains an in-frame deletion of the *vtlG* gene	This study
pKD13	Template plasmid containing the *FRT-aph(3')II-FRT* gene cassette	[17]
pKD13::*aac(3)IV*	Recombinant pKD13 with *aph(3')II* gene replaced by the *aac(3)IV* gene	This study
pKU492*aac(3)IV*	Integrating vector pKU492Acos containing apramycin resistant gene, *aac(3)IV*, for heterologous gene expression	[18]
pKU492*aac(3)IV* *-sav2794p-vtlR*	Integrating vector pKU492*aac(3)IV* containing *sav2794* promoter and LuxR-family transcriptional regulator gene, *vtlR*	[16]
pET-28b(+)	*E. coli* expression containing N-terminal His-tag for protein purification	Novagen
pET-28b(+)::*vtlG*	Recombinant pET-28b(+) with the *vtlG* gene (1206 bp) was inserted into *Nde*I and *Xho*I sites	This study
pET-28b(+)::*spiFd*	Recombinant pET-28b(+) with vector the synthetic spinach Fd gene (294 bp) inserted into *Nde*I and *Xho*I sites	This study
pET-28b(+)::*vtlF*	Recombinant pET-28b(+) with the synthetic *vtlF* gene (234 bp) inserted into *Nde*I and *Xho*I sites	This study
pET-28b(+)::*mirFd*	Recombinant pET-28b(+) with the synthetic *mirFd* gene (243 bp) inserted into *Nde*I and *Xho*I sites	This study
pET-28b(+)::*triM*	Recombinant pET-28b(+) with the synthetic *triM* gene (222 bp) inserted into *Nde*I and *Xho*I sites	This study
pET-28b(+)::*sceC*	Recombinant pET-28b(+) with the synthetic *sceC* gene (222 bp) inserted into *Nde*I and *Xho*I sites	This study
pRKSUF017	A recombinant plasmid containing the *sufABCDSE* operon for iron-sulphur (Fe-S) cluster assembly	[3]

Supplementary Table 2 Continued | Bacterial strains and vectors/plasmids.

**Bacterial strains**	**Descriptions**	**Sources**
S. avermitilis SUKA17 (SUKA17)	Clean host for heterologous gene expression: SUKA13 Δ(2633682–2641994 nt)*::mut-loxP*	[15]
SUKA17/ pKU503*vtl*	SUKA17 transformed with pKU503*vtl*	[16]
SUKA17/pKU503*vtl* /pKU492*aac(3)IV-sav2794p-vtlR*	SUKA17/pKU503*vtl* transformed with pKU492*aac(3)IV-sav2794p-vtlR*	[16]
SUKA17/pKU503*vtl*::Δ*vtlG* /pKU492*aac(3)IV-sav2794p-vtlR*	SUKA17/pKU503*vtl*::Δ*vtlG* transformed with pKU492*aac(3)IV-sav2794p-vtlR*	This study
E. coli DH5α	Host for routine cloning	Takara
E. coli BW25113/pKD46	For λ Red-mediated recombination	[17]
E. coli XL1-Blue MRF'	For FLP/*FRT*-based gene deletion	Agilent Technologies
E. coli GM2929 *hsdS*::*Tn10*	For DNA demethylation	[15]
E. coli BL21 Star™ (DE3)	For protein expression	Invitrogen
E. coli C41 OverExpress™ (DE3)	For protein expression	Biosearch Technologies

Supplementary Table 3 | PCR primers used in this study.

**For the exchange of antibiotics resistance marker gene in pKD13**
pKU492-Apr-Fwd:<br>5’-CAGAGCGCTTTTGAAGCTCACGCTGCCGCAAGCACTCAGGATCGGTATAAGACCCCGAA-3’
pKU492-Apr-Rev:<br>5’-ATAGGAACTTCGGAATAGGAACTTCAAGATCCCCTTATTATCATGAGCTCAGCCAATCGA-3’
**For in-frame deletion of *vtlG* gene in pKU503*vtl***
pKD13-Apr-Fwd:<br>5’-ATACGCGCCTATCCGTTCAGCGAACCCACCGGCTCGCACATTCCGGGGATCCGTCGACC-3’
pKD13-Apr-Rev:<br>5’-CGTCGGGAAGCGCTGTACGAGCGTGGACAGCACCGTGCTGCTGTAGGCTGGAGCTGCTTC-3’
**For PCR-confirmation of *vtlG* disruption in pKU503*vtl*::Δ*vtlG* gene**
pKU503-vtlG-Fwd: 5’-CCCGGACGAACTCACACCATTGAT-3’
pKU503-vtlG-Rev: 5’-TCACCAGGCCACCTTCAGTACCTT-3’
**For gene cloning and expression of the *vtlG* gene**
Nde-vtlG-Fwd: 5’-GGGAATTC*CATATG*GTGACCACCTCCCAGGAGA-3’
Xho-vtlG-Rev: 5’-CCG*CTCGAG*TCACCAGGCCACCTTCAGTA-3’
**For site-directed mutagenesis of spinach Fd**
SpiFd_C40A_Fwd: 5’- CCCGTATAGCgcaCGCGCAGGATC-3’
SpiFd_C40A_Rev: 5’- AGATCAATGCCTTCTTCTTC-3’
**For site-directed mutagenesis of MirFd**
MirFd_C19A_Fwd: 5’- CCGCGCGGCCgcaATCGGCACCG-3’
MirFd_C19A_Rev: 5’- TCGACGGCGACCGACCAG-3’

Homologous arms for λ-red recombination are underlined. Recognition sites of DNA restriction enzymes are indicated in italics. Lowercase letters correspond to mutated nucleotides.

Supplementary Table 4 | DNA sequences for the ferredoxin genes.

**Gene (accession #)**	**Sequence**	**Expected molecular mass (kDa)**
SpiFd<br>(M35660.1) *	*CATATG*GCCGCGTACAAAGTGACCTTAGTGACCCCTACTGGCAA<br>TGTCGAGTTCCAATGCCCAGATGACGTGTACATCCTTGATGCTG<br>CGGAAGAAGAAGGCATTGATCTCCCGTATAGCTGTCGCGCAGG<br>ATCATGCTCTAGTTGCGCTGGGAAACTGAAAACCGGTAGCTTG<br>AACCAGGATGATCAGTCCTTTCTGGATGACGACCAGATTGACG<br>AAGGTTGGGTTCTGACATGTGCGGCATATCCGGTTTCGGATGT<br>AACGATCGAAACGCATAAGAAAGAGGAACTGACCGCCTGA*CT*<br>*CGAG*	12.42
vtlF<br>(LC523631)	*CATATG*GTGAGCAACGCCGGCACCTGGGCCCTCGTCGTGGACC<br>GGGGTGCCTGCATCGGCGCCTCGGTGTGCGCGAACTACGCACC<br>CGGCCACTTCGAGCTGCGTGCCGGCAAGTCTCACGCGCTGACC<br>GGTTCGGTCGAGCCGGACCCGGCACTGCTGGACGCCGCGGAGA<br>CTTGCCCGGTGAGCGCGATCACGGTGCAGGACGCGGACACCGG<br>GGCGCTGCTGGCGCCCGAGGAATGA*CTCGAG*	9.65
mirFd<br>(CP001630.1)	*CATATG*GTGAACCACACCACCGAGGCCACCTCCTGGTCGGTCGC<br>CGTCGACCGCGCGGCCTGCATCGGCACCGGCGTGTGCGTGAGC<br>ACCTCGCCGGAGCACCTGGAGATCCGCGACGGCAAGGCCGCCG<br>CCCGCGCGACCGAGACCGGTCCCGCGCAGTACCTGCTCGACGC<br>GGCGGACATGTGCCCCATGGCCGCGATCACCGTCACCGAGACG<br>GCCACGGGCCGGGTGCTCGCACCCGAGGAGTAA*CTCGAG*	10.19
sceC<br>(KX230849.1)	*CATATG*GTGACCGGCTCCGGAGAGAGGTTCCCCGCCATGCGCGT<br>CAGCGTGGAGACCCCGCGGTGCGTGGGGTCCGGTCAGTGCGCG<br>ATGCTCACCCCGGAGGTCTTCGACCAGGACGAGGACGGGATCG<br>TCGTCCTCCTCCAGGAGGCGCCGCCGGAGGAGGTGTACGAGGA<br>CGTCCGCCAGGCGGCCATGACGTGCCCGGTCCAGGTCATCAGC<br>CTCGACGAATAG*CTCGAG*	9.76
trim<br>(MN305323.1)	*CATATG*GTGACGGCCGACCCGGACGGAGTCCCCCTCATGCGTGT<br>CACCGCCGACCGCGACCGGTGCGTGGGCTCCGGCCAGTGCGCG<br>ATGCTCAGTCCCGAGGTGTTCGACCAGGACGACGACGGCCTCG<br>TCCTGGTCCTGCGGGAGGTGCCGGCCGCGGATCTCCACGAGGA<br>GGTGCACCGGGCCGCCGACCTGTGCCCGGCGCGCTCGATCCAG<br>GTGCAGGACTGA*CTCGAG*	10.19

Recognize sites of DNA restriction enzymes are indicated in italics.

*Codon of *spiFd* gene was optimized for expression in *E. coli*.

Supplementary References

1 Takahashi, S. *et al.* Structure-function analyses of cytochrome P450revI involved in reveromycin A biosynthesis and evaluation of the biological activity of its substrate, reveromycin T. *J. Biol. Chem.* **289**, 32446-32458 (2014).

2 Omura, T. & Sato, R. A new cytochrome in liver microsomes. *J. Biol. Chem.* **237**, 1375-1376 (1962).

3 Takahashi, Y. & Tokumoto, U. A third bacterial system for the assembly of iron-sulfur clusters with homologs in archaea and plastids. *J. Biol. Chem.* **277**, 28380-28383 (2002).

4 Mutoh, R. *et al.* X-ray Structure and Nuclear Magnetic Resonance Analysis of the Interaction Sites of the Ga-Substituted Cyanobacterial Ferredoxin. *Biochemistry* **54**, 6052-6061 (2015).

5 Kim, H. J., Ruszczycky, M. W., Choi, S. H., Liu, Y. N. & Liu, H. W. Enzyme-catalysed [4+2] cycloaddition is a key step in the biosynthesis of spinosyn A. *Nature* **473**, 109-112 (2011).

6 Tian, Z. *et al.* An enzymatic [4+2] cyclization cascade creates the pentacyclic core of pyrroindomycins. *Nat. Chem. Biol.* **11**, 259-265 (2015).

7 Kato, N. *et al.* A new enzyme involved in the control of the stereochemistry in the decalin formation during equisetin biosynthesis. *Biochem. Biophys. Res. Commun.* **460**, 210-215 (2015).

8 Drulyte, I. *et al.* Crystal structure of the putative cyclase IdmH from the indanomycin nonribosomal peptide synthase/polyketide synthase. *IUCrJ* **6**, 1120-1133 (2019).

9 Hwang, S. *et al.* Structure Revision and the Biosynthetic Pathway of Tripartilactam. *J. Nat. Prod.* **83**, 578-583 (2020).

10 Shen, J. *et al.* Polycyclic Macrolactams Generated via Intramolecular Diels-Alder Reactions from an Antarctic *Streptomyces* Species. *Org. Lett.* **21**, 4816-4820 (2019).

11 Hoshino, S. *et al.* Mirilactams C-E, Novel Polycyclic Macrolactams Isolated from Combined-Culture of *Actinosynnema mirum* NBRC 14064 and Mycolic Acid-Containing Bacterium. *Chem. Pharm. Bull.* **66**, 660-667 (2018).

12 Beemelmanns, C. *et al.* Macrotermycins A-D, Glycosylated Macrolactams from a Termite-Associated *Amycolatopsis* sp. M39. *Org. Lett.* **19**, 1000-1003 (2017).

13 Wang, J. *et al.* Genome-Guided Discovery of Pretilactam from *Actinosynnema pretiosum* ATCC 31565. *Molecules* **24**, 2281 (2019).

14 Low, Z. J. *et al.* Identification of a biosynthetic gene cluster for the polyene macrolactam sceliphrolactam in a *Streptomyces* strain isolated from mangrove sediment. *Sci. Rep.* **8**, 1594 (2018).

15 Komatsu, M., Uchiyama, T., Omura, S., Cane, D. E. & Ikeda, H. Genome-minimized *Streptomyces* host for the heterologous expression of secondary metabolism. *Proc. Natl. Acad. Sci. U.S.A.* **107**, 2646-2651 (2010).

16 Nogawa, T. *et al.* Heterologous Expression of the Biosynthetic Gene Cluster for Verticilactam and Identification of Analogues. *J. Nat. Prod.* **83**, 3598–3605 (2020).

17 Datsenko, K. A. & Wanner, B. L. One-step inactivation of chromosomal genes in Escherichia coli K-12 using PCR products. *Proc. Natl. Acad. Sci. U.S.A.* **97**, 6640-6645 (2000).

18 Komatsu, M. *et al.* Engineered *Streptomyces avermitilis* host for heterologous expression of biosynthetic gene cluster for secondary metabolites. *ACS Synth. Biol.* **2**, 384-396 (2013).
